# Potent, Selective, and Drug‐Like G Protein‐Coupled Receptor Kinase 5 and 6 Inhibitors: Design, Synthesis, and X‐Ray Structural Studies

**DOI:** 10.1002/cmdc.202500257

**Published:** 2025-09-29

**Authors:** Arun K. Ghosh, Ranjith Kumar Gadi, Yueyi Chen, Sandali Piladuwa Gamage, Kathryn P. McCauley, John J. G. Tesmer

**Affiliations:** ^1^ Department of Chemistry Purdue University West Lafayette IN 47907 USA; ^2^ Department of Medicinal Chemistry and Molecular Pharmacology Purdue University West Lafayette IN 47907 USA; ^3^ Department of Biological Sciences Purdue University West Lafayette IN 47907 USA

**Keywords:** GRK2, GRK5 inhibitors, GRK6 inhibitors, heterocyclic, selectivity, sunitinib, X‐ray crystal structures

## Abstract

Herein, the design, synthesis, and evaluation of small molecules, drug‐like G protein‐coupled receptor kinase 5 (GRK5) inhibitors are reported. GRK5 has become an important drug development target against heart failure and cancer. GRK6, a close homolog of GRK5, is considered as a possible therapeutic target for multiple myeloma. A series of drug‐like GRK5 inhibitors that form noncovalent interactions in the GRK5 active site are designed. In the design of these molecules, pyrroloindoline basic scaffold of sunitinib, an FDA‐approved drug, is utilized and various *N*‐heterocyclic carboxamides in the active site are incorporated. Several inhibitors exhibit low nanomolar GRK5 inhibitory activity and high selectivity over GRK2. Several compounds also display very potent activity and selectivity for GRK6. A high‐resolution X‐ray crystal structure of one of these small molecule inhibitors in complex with GRK5 is determined. The structure provides important molecular insights regarding ligand‐binding site interactions, GRK5 inhibition, and selectivity against GRK2.

## Graphical abstract

Herein, X‐ray structure‐based design, synthesis, and evaluation of a series of potent and selective small molecules G protein‐coupled receptor kinase 5 (GRK5) and GRK6 inhibitors that exhibit high selectivity against GRK2 are reported. A high‐resolution X‐ray crystal structure of GRK5 and inhibitor **6t** complex provides important molecular insight for further optimization of inhibitor properties.

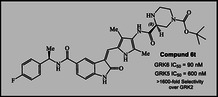

## 1 Introduction

Due to their widespread involvement in regulating a diverse range of physiological processes, G protein‐coupled receptors (GPCRs) are considered one of the most important families of cell surface receptors in humans.^[^ ^1^, ^2^ ^]^ Ligand‐associated GPCRs trigger a series of cellular events by activating heterotrimeric G proteins in the cytoplasmic domain setting up intracellular signaling responses. G protein receptor kinases (GRKs) are then recruited to phosphorylate the agonist‐bound receptor on its cytoplasmic loops, leading to binding of β‐arrestin to the receptor, which can block recoupling of the dissociated G‐protein subunit to the GPCR and also target the complex to clathrin‐coated pits. This ultimately leads to internalization and desensitization of GPCR signal transduction and blocks hyperactivation of GPCR second‐messenger cascades.^[^ ^3^, ^4^ ^]^ The key role of GRKs in the regulation of GPCR signaling and maintenance of homeostatic cell functions has been extensively studied over the years. The imbalance or abnormal GRK activity has been associated with diverse disease processes including heart failure and malignancies.^[^ ^5^, ^6^, ^7^ ^]^ The GRKs are a family of seven serine/threonine kinases and are classified into three subfamilies (GRK1/7, GRK2/3, and GRK4,5,6) based upon homology.^[^ ^8^, ^9^ ^]^ They display both tissue specific expression and selectivity for GPCRs, with GRK1/7 in the retina, GRK4 in the testes, and GRK6 in neuronal and immune cells.^[^ ^4^, ^10^, ^11^ ^]^ Both GRK5 and GRK6 are expressed ubiquitously.

GRK activity and expression are featured prominently in many human disease pathologies, including cardiovascular diseases which are major public health problems leading to significant morbidity and mortality around the globe. In particular, heart failure (HF) affects an estimated 64 million patients worldwide.^[^ ^12^, ^13^ ^]^ Despite the availability of treatment options, the mortality for patients with HF is quite high.^[^ ^14^ ^]^ GRK5 is often overexpressed in hearts undergoing hypertrophy.^[^ ^15^ ^]^ It shows noncanonical activity in the nucleus of cardiomyocytes by acting as a regulator of pathological cardiac hypertrophic gene transcription.^[^ ^16^, ^17^ ^]^ Thus GRK5 has emerged as an important drug development target for HF treatment. GRK5 is also a modulator of cancer biology through regulation of chemokine receptor and nonreceptor substrates including tumor suppressor 53 and moesin which contribute to many types of cancers.^[^ ^18^, ^19^ ^]^ Interestingly, GRK6 a close homolog of GRK5, is highly expressed in lymphoid cells where it regulates CXC motif chemokine receptor 4, a GPCR responsible for neutrophil mobility and cancer metastasis.^[^ ^20^, ^21^ ^]^ GRK6 is overexpressed in multiple myeloma (MM) cells and knock‐down studies of GRK6 have been shown to cause apoptosis in six of the seven MM cells but was tolerated in seven of seven human cell lines.^[^ ^11^, ^22^ ^]^ These studies suggested GRK6 as a unique drug target and selective GRK6 inhibition may be effective in the treatment of human MM.^[^ ^23^, ^24^ ^]^

**Figure 1:**
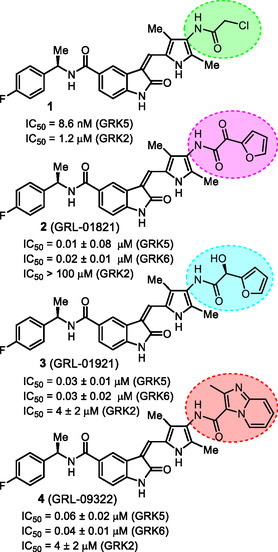
Structures and activity of recent pyrroloindoline‐derived GRK5 inhibitors **1–4**.

The development of selective GRK5 and GRK6 inhibitors has become a significant recent interest in academic and pharmaceutical laboratories.^[^ ^25^, ^26^ ^]^ However, the close homology and function of GRKs make the development of selective inhibitors particularly challenging. Recently, a number of strategies for GRK5 inhibitors, including allosteric inhibitors and covalent inhibitors have been reported.^[^ ^27^, ^28^, ^29^, ^30^, ^31^ ^]^ For GRK6 inhibitors, there is only one recent report of small molecule GRK6 inhibitors by Uehling and co‐workers.^[^ ^32^ ^]^ Our laboratories are involved in the design and synthesis of both GRK5 and GRK6 inhibitors. We reported the design of covalent GRK5 inhibitors containing a haloacetamide warhead on a pyrroloindoline scaffold which is inherent to sunitinib, an FDA‐approved drug that blocks receptor tyrosine kinases.^[^ ^33^, ^34^ ^]^ These inhibitors were designed to form a covalent bond with the cysteine residue in GRK5 active sites.^[^ ^35^ ^]^ Representative chloroacetamide inhibitor **1** (**Figure** **1**) displayed a GRK5 IC_50_ value of 8.6 nM and a GRK2 activity of 1.2 μM, showing over 1400‐fold selectivity over GRK2. Our intact mass spectroscopic studies revealed that chloroacetic derivative **1** formed a covalent bond with GRK5, likely with Cys474, a GRK5 subfamily‐specific residue located within the flexible loop adjacent to active site.^[^ ^35^ ^]^ Unfortunately, inhibitors with such reactive warheads may form irreversible adducts with off‐target proteins with exposed cysteines or reactive nucleophiles, leading to side effects and toxicities. Therefore, the design of reversible covalent inhibitors which generally prevents accumulation of off‐target adducts or design of noncovalent inhibitors are generally preferred to limit side effects.^[^ ^36^ ^]^

We recently reported the design of a class of selective GRK5 inhibitors by incorporating a ketoamide functionality as the warhead that would form a covalent bond with Cys474 in the GRK5 active site.^[^ ^37^ ^]^ We speculated that the formation of such hemithioacetal intermediate would be reversible in nature. A representative inhibitor **2** exhibited a GRK5 IC_50_ value of 10 nM and GRK2 activity of 1 mM, showing a selectivity over 100,000‐fold against GRK2. A high‐resolution X‐ray structure of compound **2**‐bound to GRK5 showed the formation of the hemithioacetal intermediate.^[^ ^37^ ^]^ Compound **2** displayed a GRK6 IC_50_ value of 20 nM, which is not surprising because GRK6 maintains a close homology with GRK5.^[^ ^11^, ^22^ ^]^ Based upon these X‐ray structural insights, we then speculated that a stereochemically defined *α*‐hydroxyamide functionality can bind to the active site of GRK5 and may be able to inhibit GRK5 in a noncovalent manner. Indeed, compound **3** with (*S*)‐*α*‐hydroxyamide exhibited GRK5 IC_50_ value of 33 nM. This compound showed good selectivity over GRK2 (over 120‐fold) but significantly less than inhibitor **2**. Our high‐resolution X‐ray structural studies of compound **3**‐bound GRK5 revealed a noncovalent interaction with the active site tether (AST loop).^[^ ^38^ ^]^ The corresponding (*R*)‐isomer was significantly less potent and less selective over GRK2. Compound **3** showed a similar GRK6 IC_50_ value as GRK5. Subsequently, we pursued structure‐based design of noncovalent GRK5 inhibitors by incorporating heterocyclic carboxamide derivatives to interact with the GRK5 AST loop. These studies resulted in a series of potent and selective small molecule GRK5/6 inhibitors as potential leads. For example, compound **4** with an imidazo‐pyridine heterocycle, exhibited GRK5 and GRK2 IC_50_ value of 60 nM, and 50 μM, respectively,^[^ ^38^ ^]^ showing over 800‐fold selectivity over GRK2. Compound **4** displayed a similar GRK6 IC_50_ value (40 nM) as GRK5. The X‐ray structure of compound **4**‐bound GRK5 and comparison with structures of GRK2 revealed potential steric clashes with the GRK2 AST loop that may be responsible for excellent selectivity of compound **4** against GRK2. We have now expanded our search for small molecule heterocyclic scaffolds that show additional selectivity, particularly against both GRK2 and GRK5. Herein, we report a new set of noncovalent GRK5 inhibitors containing a pyrroloindoline basic scaffold and *N*‐heterocyclic carboxamides in the active site to interact with the AST loop. A number of these derivatives potently inhibited both GRK5 and GRK6. The X‐ray structure of one of these inhibitors in complex with GRK5 provided molecular insights into the ligand‐binding site interactions responsible for this high activity. The results of these current studies may enable further optimization of potency and selectivity of GRK inhibitors.

## 2 Results and Discussion

**Figure 2:**
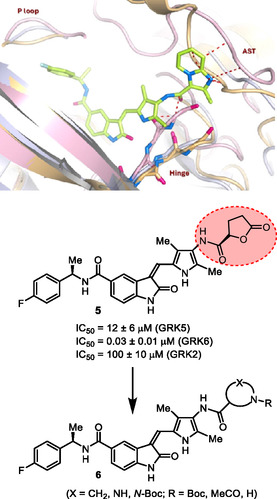
X‐ray structure of compound **4**‐GRK5 complex, PDB entry 9BRJ. Compound **4** does not form covalent bond with AST and red dashes represent steric clashes with AST. Recent design of a noncovalent inhibitor **5** containing a lactone carboxamide ligand (magenta color) is highlighted. Current design of *N*‐cyclic amine carboxamide derivatives is shown in structure **6**.

Our recent structure‐based design studies provided a number of important molecular insights for optimization of the potency and selectivity of GRK5/6 inhibitors. As mentioned, compound **4** with an imidazopyridine carboxamide in the active site exhibited a potent GRK5 IC_50_ value of 60 nM and relatively good >800‐fold selectivity against GRK2 (IC_50_ 50 μM). Compound **4** is quite active against GRK6 and did not show any selectivity against GRK5. To understand this intriguing selectivity against GRK2, as shown in **Figure** **2**, we compared the AST loop conformation of GRK2 with that of compound **4**‐bound GRK5 X‐ray structure from our previous studies.^[^ ^38^ ^]^ As it turns out, the imidazo‐pyridine group makes favorable van der Waals interaction with the AST of GRK5, but clashes with the modeled AST loop of GRK2.

Our recent studies of noncovalent inhibitors also provided GRK6 selective compound **5,** with a 3‐(*R*)‐tetrahydrofuranyl heterocycle.^[^ ^38^ ^]^ Compound **5** displayed GRK6 IC_50_ value of 32 nM and GRK5 IC_50_ value of 12 μM, thus exhibiting over 400‐fold selectivity against GRK5. However, it showed only eightfold selectivity against GRK2. Interestingly, the compound with a 3(*S*)‐isomer also displayed potent activity against GRK6 (IC_50_ 20 nM), however, less selective (35‐fold) against GRK5. Based upon these molecular and structural insights, we planned to investigate the effect of various *N*‐cyclic amine carboxamide derivatives as reported in general structure **6**. We speculated that ring stereochemistry as well as the substituent on the nitrogen may confer favorable interaction with GRK5/GRK6 but set up steric clashes with the GRK2 AST loop.

### 2.1 Syntheses of Proposed GRK Inhibitors 6a–v

**Scheme 1:**
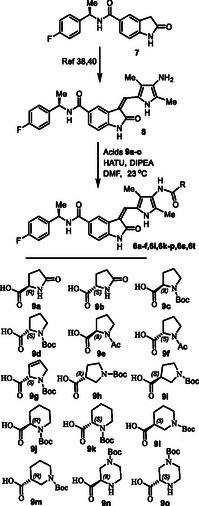
Synthesis of GRK inhibitors containing *N*‐cyclic carboxamide derivatives.

**Scheme 2:**
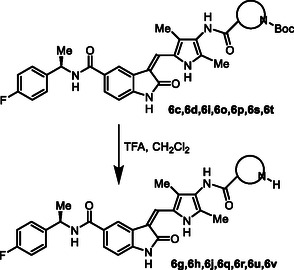
Synthesis of GRK inhibitors containing *N*‐cyclic amine and *N*‐cyclic acetamide derivatives.

The synthesis of various *N*‐cyclic amine derived GRK5/GRK6 inhibitors is shown in **Scheme** **1**. Pyrroloindoline amine derivative **8** was prepared from the known indoline derivative **7** as described previously.^[^ ^37^, ^39^ ^]^ Selected optically active carboxylic acids **9a–o** with *N*‐cyclic amine functionalities are available commercially. Coupling these carboxylic acids with amine **8** using hexafluorophosphate azabenzotriazole tetramethyl uronium (HATU) in DMF in the presence of excess diisopropylethylamine (DIPEA) at 0–23 °C for 12 h afforded carboxamide derivatives in good to excellent yields (52–75%). As shown in **Scheme** **2** for the synthesis of amine derivatives, the corresponding Boc‐derivatives **6c, 6d, 6i, 6o, 6p,** **6s** and **6t** were treated with trifluoroacetic acid in CH_2_Cl_2_ at 0–23 °C for 2 h to provide inhibitors **6g, 6h, 6j, 6q, 6r, 6u,** and **6v** in excellent yield (81–92%). Full structures of these inhibitors are shown in **Table** **1**.

**Table 1:** Structure and activity of inhibitors of tubulin phosphorylation by GRKs.

Inhibitor structure	IC_50_ [μM]a) GRK5	IC_50_ [μM] GRK6	IC_50_ [μM] GRK2	Selectivity GRK2/GRK5b)	Selectivity GRK5/GRK6b)
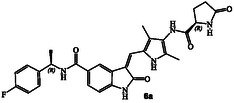	0.027 ± 0.006	0.020 ± 0.01	0.71 ± 0.4	26	1
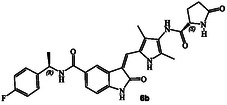	0.8 ± 0.4	0.028 ± 0.002	2.1 ± 0.2	3	30
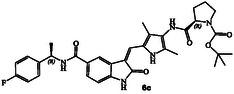	50 ± 30	1.4 ± 0.5	160 ± 100	>3	35
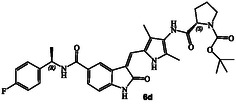	0.44 ± 0.16	9.9 ± 2c)	>1000	>2000	–
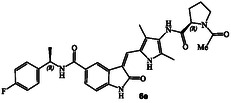	0.040 ± 0.006	0.06 ± 0.02	0.32 ± 0.09	8	1
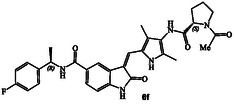	0.060 ± 0.02	0.031 ± 0.02	0.90 ± 0.3	15	2
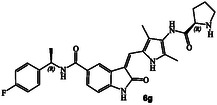	0.040 ± 0.02	0.030 ± 0.002	0.1 ± 0.01	3	1
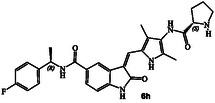	0.090 ± 0.04	0.034 ± 0.01	0.08 ± 0.01	1	3
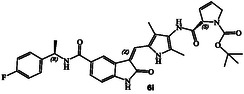	0.92 ± 0.5	0.5 ± 0.2	120 ± 50	130	2
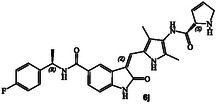	0.03 ± 0.004	0.03 ± 0.01	0.48 ± 0.4	16	1
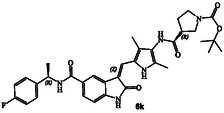	2.1 ± 0.9	1.4 ± 0.2	300 ± 100	140	1
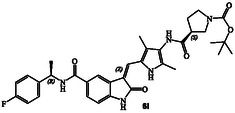	1.0 ± 0.6	0.88 ± 0.05	>1000	>1000	1
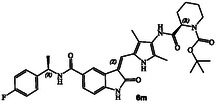	0.50 ± 0.1	2.5 ± 0.4	1.2 ± 0.4	2	–
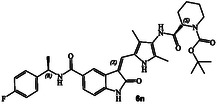	33 ± 6	0.90 ± 0.8	100 ± 70	3	37
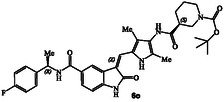	0.094 ± 0.01	0.15 ± 0.06	6.2 ± 3	66	1
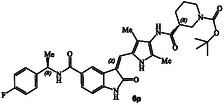	0.41 ± 0.1	0.34 ± 0.3	610 ± 450	1500	1
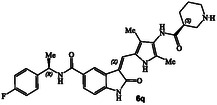	0.03 ± 0.02	0.016 ± 0.003	0.11 ± 0.02	4	2
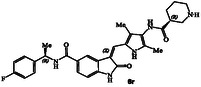	0.03 ± 0.002	0.06 ± 0.02	0.62 ± 0.3	20	1
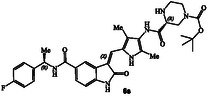	0.081 ± 0.01	0.06 ± 0.02	35 ± 9.4	430	1
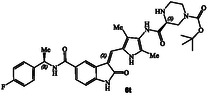	0.6 ± 0.1	0.09 ± 0.5	>1000	>1600	7
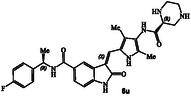	0.014 ± 0.001	0.05 ± 0.5	0.21 ± 0.1	15	–
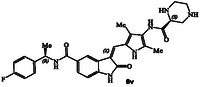	0.016 ± 0.0005	0.026 ± 0.02	0.19 ± 0.1	10	1

a) IC_50_ values for GRK5, GRK6, and GRK2 phosphorylation of the soluble substrate tubulin. Error bars are standard deviation from 3 to 4 independent experiments.

b) GRK2/GRK5 indicates fold selectivity for GRK5 over GRK2, and GRK5/GRK6 the fold selectivity for GRK6 over GRK5.

c) This compound exhibited nonsigmoidal behavior with GRK6, suggesting that other inhibitory processes were occurring other than equilibrium binding.

### 2.2 Structure–Activity Relationship Studies

The inhibitory potency of synthetic compounds containing the *N*‐heterocyclic amide functionalities was evaluated against human GRK2, GRK5 and GRK6 using our in vitro assays‐, as reported previously.^[^ ^35^, ^37^ ^]^ The structure and activity of these inhibitors are shown in Table 1. Compound **6a** with (*R*)‐pyrrolidone functionality showed very potent GRK5 inhibitory activity with IC_50_ value of 27 nM. In comparison, compound **6b** with (*S*)‐pyrrolidone functionality exhibited 30‐fold reduction of GRK5 activity, displaying GRK5 IC_50_ of 800 nM. Interestingly, both **6a** and **6b** showed very potent GRK6 activity (IC_50_ values 20 and 28 nM), showing no stereochemical preference by the GRK6‐binding site. Both compounds showed only a threefold difference in their activity against GRK2. However, compound **6a** exhibited 26‐fold selectivity over GRK2. We then investigated the effect of (*R*)‐ and (*S*)‐Boc pyrrolidine carboxamide derivatives. Compound **6d** with (*S*)‐Boc substituent exhibited over 100‐fold enhanced GRK5 inhibitory activity over the (*R*)‐Boc derivative **6c**. Interestingly, the corresponding *N*‐acetyl derivatives **6e** and **6f** showed very potent inhibitory IC_50_ values against GRK5 and GRK6, but selectivity among the GRKs was marginal. The removal of Boc groups from **6c** and **6d** resulted in pyrrolidine derivatives **6g** and **6h** with (*S*)‐ and (*R*)‐configuration. Both compounds showed very good inhibitory activity against GRKs but likewise displayed little to no selectivity. These improvements of GRK5 IC_50_ values over their Boc‐derivatives (**6c** and **6d**) indicated the bulky Boc group may not be able to fit as well in the catalytic site of these GRKs. On the other hand, sterically less demanding acetamide derivatives **6e** and **6f** were well accommodated consistent with their very good activity against GRK5, GRK6, and GRK2, but only with marginal selectivity against GRK2. The (*S*)‐Boc‐2,5‐dihydro‐pyrrole derivative **6i** exhibited sub‐micromolar GRK5 and GRK6 IC_50_ values and exhibited 130‐fold selectivity against GRK2. The removal of Boc group resulted in compound **6j** which exhibited GRK5 and GRK6 IC_50_ values of 30 nM and GRK2 activity of 480 nM, or over 15‐fold selectivity. We also examined the effect of 3(*S*)‐ and 3(*R*)‐Boc‐pyrrolidine derivatives. These derivatives **6k** and **6i** showed GRK5 and GRK6 inhibitory IC_50_ values in low micromolar range. Interestingly, these compounds showed little to no activity against GRK2, and thus compound **6i** showed excellent selectivity against GRK2.

We then investigated how a slightly larger ring cycle would interact with residues in the AST region. Compounds **6m** and **6n** with 2(*S*)‐ and 2(*R*)‐Boc‐piperidine core did not significantly improve activity against GRK5 and GRK6 compared to the corresponding pyrolidine derivatives **6c** and **6d**. The corresponding 3(*S*)‐ and 3(*R*)‐Boc‐piperidine derivatives **6o** and **6p** improved activity against GRK5, showing IC_50_ values of 94 and 410 nM, respectively. Particularly, 3(*S*)‐isomer **6o** showed stereochemical preference over 3(*R*)‐isomer **6p**, showing a 66‐fold selectivity against GRK2. The selectivity over GRK6 however, was marginal. Compound **6p** with 3(*R*)‐configuration showed comparable GRK5 and GRK6 IC_50_ values of 410 and 340 nM, respectively, but exhibited excellent selectivity against GRK2, over 1500‐fold. The removal of the Boc‐group resulted in compounds **6q** and **6r** showing GRK5 IC_50_ values of 30 nM. However, their selectivity against GRK2 was marginal. We then investigated the effect of Boc‐piperazine derivatives **6s** and **6t** with the incorporation of an additional ring nitrogen. Compound **6s** with 2(*R*)‐configuration displayed IC_50_ values of 81 and 60 nM against GRK5 and GRK6, respectively, over fivefold improvement over the corresponding 3(*R*)‐piperidine derivative **6p**. Compound **6s** with a basic amine showed over fivefold improvement of GRK5 and GRK6 activity over compound **6p** in which an NH group of **6s** is replaced by a CH2 group. The results indicate that the NH group of **6s** may be involved in hydrogen bonding interactions with GRK5. Compound **6t**, with 2‐(*S*)‐configuration is nearly tenfold less active than the compound **6s**, however it is highly selective, showing over 1600‐fold selectivity against GRK2. Interestingly, compound **6t** with a basic amine is less potent than compound **6o** in which an NH group of **6t** is replaced by a CH2 group with identical stereochemistry. Similar to what we have seen for other de‐Boc derivatives, the resulting compounds **6u** and **6v** after removal of Boc‐group exhibited improved potency against GRK5 and GRK6, but significantly reduced selectivity against GRK2. In essence, de‐Boc derivatives containing piperidine and piperazine heterocycles (**6q**, **6r**, **6u,** and **6v**) irrespective of the ring stereochemistry or number of basic amines on the ring, show similar affinity for GRK5 or GRK6. Also, these de‐Boc derivatives are significantly less selective against GRK2 compared to their Boc counterparts (**6o**, **6p**, **6s**, and **6t**). The six‐membered heterocyclic ring in **6q, 6r, 6u,** and **6v**, irrespective of ring stereochemistry, are suitably accommodated in the hydrophobic pocket adjacent to the AST loop. The X‐ray structural studies will shed light into these important ligand‐binding site interactions.

### 2.3 X‐Ray Structure of Compound 6t in Complex with GRK5

**Figure 3:**
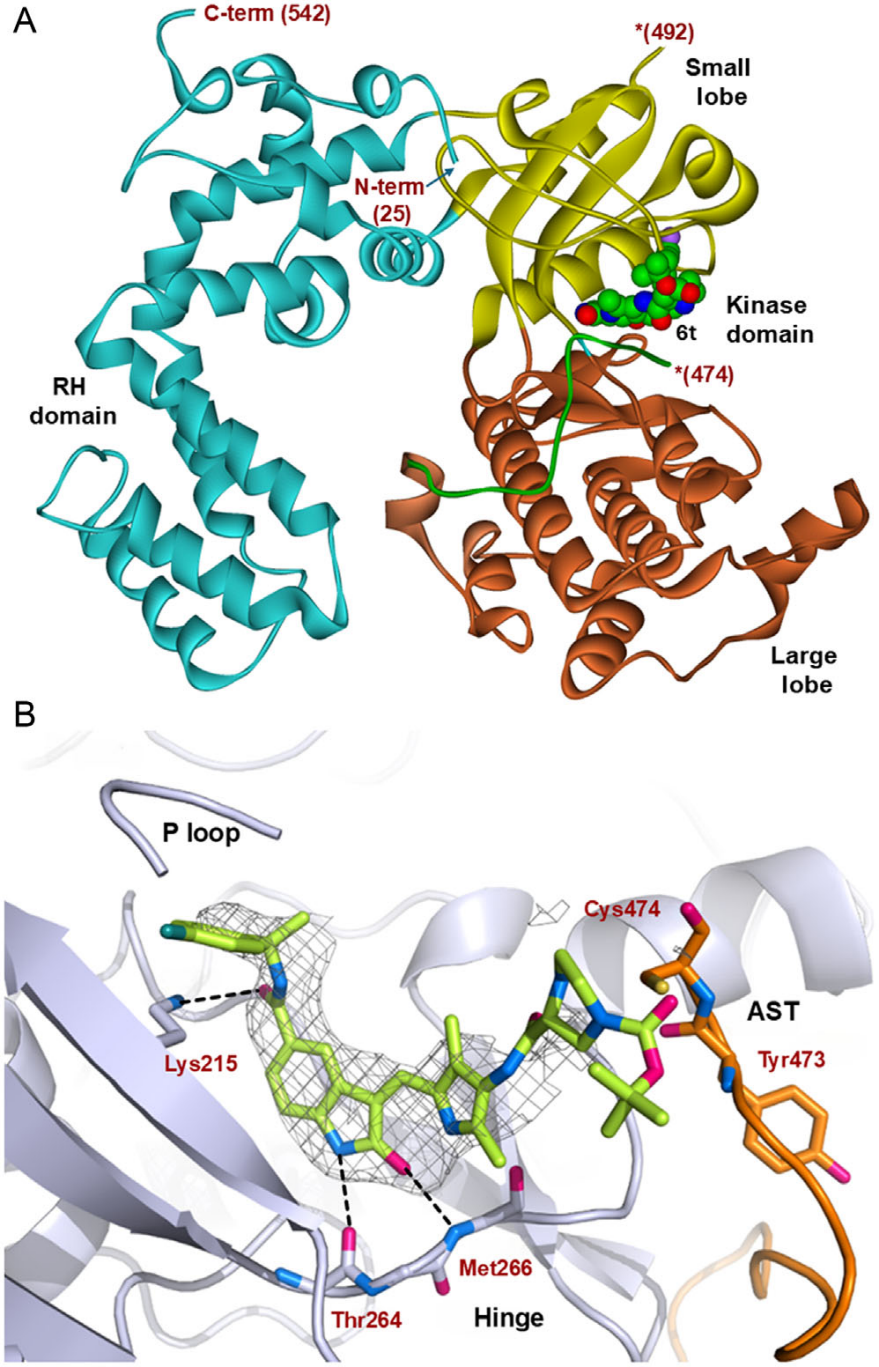
The X‐ray crystal structure of inhibitor **6t** in complex with GRK5 (PDB entry 9BRK). A) GRK5 kinase domain is in a more open and inactive conformation. RH domain (turquoise). Compound **6t** (cpk, green carbons) binds to the active site of GRK5 in the kinase domain. The AST loop (green) is ordered up to Cys474. Asterisks indicate the last residue at the end of disordered region. B) Inhibitor **6t** (green carbons) is bound in the GRK5 active site pocket (gray cartoon) which includes an ordered region of hinge region and P‐loop region. Polder omit map for the bound ligand is contoured at 2.5 *σ* cutoff (gray mesh). Hydrogen bonds are shown with black dashed lines.

**Figure 4:**
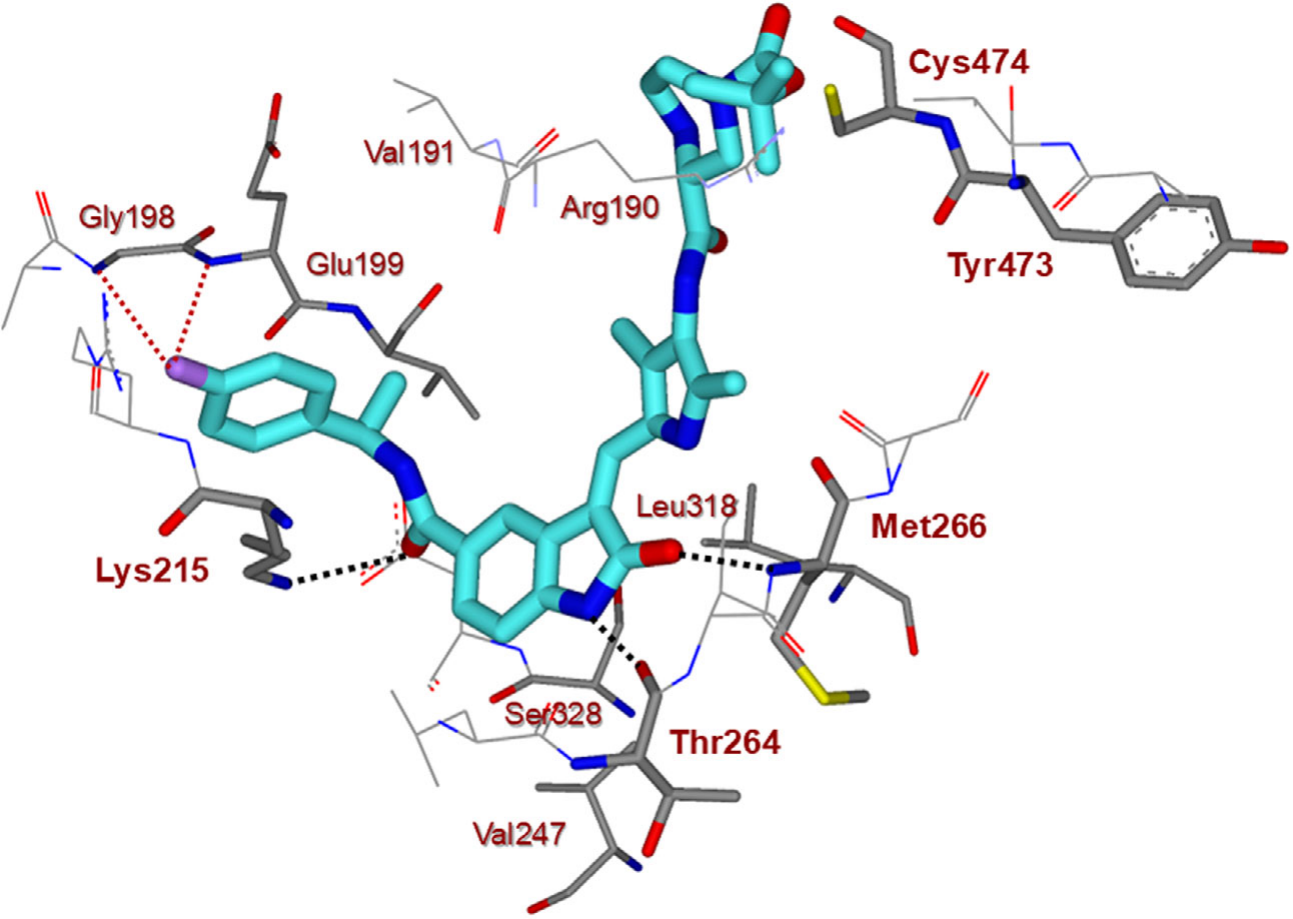
Inhibitor **6t**‐bound GRK5 X‐ray structure (PDB entry 9BRK) is shown. The major active site interactions of **6t** with GRK5 are depicted. The inhibitor carbon atoms are shown in turquoise fluorine is purple, and hydrogen bonds are indicated by black dotted lines. The van der Waals interactions of fluorine with Gly198 and Glu199 backbone amides are shown with red dotted lines.

Because wild‐type human GRK5 expressed in *E* *scherichia* *coli* undergoes heterogeneous hyperphosphorylation, a catalytically inactive mutant GRK5_D11N_, which retains stability of protein, was used for cocrystallization.^[^ ^40^ ^]^ For X‐ray structural studies, inhibitor **6t** was soaked into crystals of GRK5_D11N_ grown in the presence of sangivamycin (sgv) that were harvested under low salt conditions.^[^ ^41^ ^]^ The 2.78 Å crystal structure of **6t**‐bound GRK5 (**Figure** **3**A, Supplemental Table 1) exhibits an “open” conformation with the angle of opening between the kinase small lobe and the kinase large lobe around 11° more than the structure of sgv‐bound GRK5 (protein database (PDB):6PJX).^[^ ^42^ ^]^ Inhibitor **6t** binds to the catalytic site of GRK5. The key interactions of inhibitor **6t** with the GRK5 catalytic site are highlighted in **Figure** **4**. The overall structure is consistent with other inhibitor‐bound GRK5 structures where inhibitor **6t** occupies the ATP‐binding site. The indoline heterocyclic core binds in the adenine subsite forming two strong hydrogen bonds with the GRK5 hinge backbone amide NHs of Thr264 and Met266. The indoline ring occupies the hydrophobic pocket formed by Val247, Leu318, and Ser328.

The indoine amide carbonyl forms a hydrogen bond with the Lys215 side chain. The (*R*)‐fluorophenylmethyl side chain of **6t** nestles in the polyphosphate subsite, a hydrophobic pocket formed by Lys215, Glu199, Val200, and Leu 217 under the P‐loop. The *para*‐fluorine atom forms van der Waals interactions with the backbone atoms of Gly198 and Glu199. The (3*S*)‐Boc‐piperazine carboxamide packed the hydrophobic pocket adjacent to the AST loop. There is no hydrogen bond interaction with the Cys474 in the AST loop. The density of this ligand core is not fully resolved possibly due to the bulky *t*‐butyl group extending out of the binding pocket. It appears that the *tert*‐butyl group is exposed to the solvent.

We showed in this series that the position of the Boc‐group and the six‐membered ring size are important for selectivity. To understand the origin of this selectivity, we compared our X‐ray structure of **6t**‐bound GRK5 and a docked model of compound **6t** with GRK2, either with its kinase domain relatively closed (PDB entry 8JPB)^[^ ^43^ ^]^ or open (PDB entry 5UKM). The Boc‐piperazine heterocycle can be accommodated by GRK5, but the ligand would have a steric clash with the GRK2 AST in either representative structure (Figure S1, Supporting Information). The removal of the bulky Boc groups in compounds **6o**, **6p**, **6s**, and **6t** provided de‐Boc derivatives **6q**, **6r**, **6u**, and **6v** with better potency against GRK5, GRK6, and GRK2, but with significantly lower selectivity against GRK2, consistent with the steric clash seen between the bulky Boc‐group and the GRK2 AST region. Several compounds in this series were considerably more potent against GRK6 than GRK5 (e.g., **6c**), possibly because the AST region of GRK6 adopts distinct configuration (PDB entry 2ACX)^[^ ^44^ ^]^ where its Cys474 and Tyr473 are about 15 Å away from the position of these GRK5 residues in the current X‐ray structure.

## 3 Conclusion

GRK5 is a potential target for the treatment of heart failure because of its involvement as a regulator of pathological cardiac hypertrophy. GRK6, a close homolog of GRK5, is a promising target for human multiple myeloma. In our current study, we designed a series of very potent GRK5 inhibitors, including compounds **6s** and **6t,** that exhibited high selectivity against GRK2. These inhibitors have *N*‐heterocyclic carboxamide functionalities on a pyrroloindoline basic scaffold. We have also identified a number of potent and relatively selective GRK6 inhibitors, including **6b** and **6n** that showed high selectivity against GRK2 and good selectivity against GRK5. Compounds **6s** and **6t** also showed very potent GRK6 inhibitory activity and selectivity against GRK2. However, their selectivity against GRK5 was limited. Interestingly, the corresponding compounds **6u** and **6v**, lacking a bulky Boc‐group, showed very potent GRK5, GRK6 inhibitory activity, but their selectivity against GRK2 was marginal. To assess the origin of potency and selectivity, we cocrystallized inhibitor **6t**‐bound GRK5 and determined X‐ray structure at 3.7 Å resolution. The X‐ray structure provided insights into the ligand‐binding site interactions responsible for potency and selectivity against GRK2. Among compounds examined, the piperazine carboxamide with the *tert*‐butyloxycarbonyl group appears to fill in the hydrophobic pocket under the AST loop. However, this bulky alkyl group would impose steric clashes with the AST loop of GRK2 and raise selectivity against GRK2 (Figure S1, Supporting Information). Both stereoisomers **6u** and **6v** without the Boc‐group, exhibited very potent GRK5 and GRK6 activity but little selectivity against GRK2. Further structural analysis of the molecular basis for selectivity and the design of more potent and selective inhibitors are in progress.

## 4 Experimental Section

4.1

4.1.1

##### General

All reactions were carried out under an argon atmosphere in either flame‐ or oven‐dried (120 °C) glassware. All reagents and chemicals were purchased from commercial suppliers and used without further purification unless otherwise noted. Anhydrous solvents were obtained as follows: dichloromethane and diisopropylethylamine (DIPEA) were distilled over calcium hydride. All purification procedures were carried out with reagent‐grade solvents (purchased form VWR) in air. Thin‐layer‐chromatography was conducted using glass‐backed thin‐layer silica gel chromatography plates (60 Å, 250 μm thickness, F‐254 indicator). Column chromatography was performed using 230–400 mesh, 60 Å pore diameter silica gel. ^1^H, ^13^C NMR spectra were recorded at room temperature on a Bruker AV800, DRX‐500, ARX‐400. Chemical shifts (*δ* values) are reported in parts per million, and are referenced to the deuterated residual solvent peak. NMR data are reported as: *δ* value (chemical shift, *J*‐value (Hz), integration, where s = singlet, d = doublet, t = triplet, q = quartet, brs = broad singlet). Low‐resolution mass spectrometry and high‐resolution mass spectrometry (HRMS) spectra were recorded at the Purdue University, Department of Chemistry Mass Spectrometry Center.

##### (Z)‐3‐((3,5‐Dimethyl‐4‐((S)‐5‐Oxopyrrolidine‐2‐Carboxamido)‐1H‐Pyrrol‐2‐Yl)Methylene)‐N‐((R)‐1‐(4‐Fluorophenyl)Ethyl)‐2‐Oxoindoline‐5‐Carboxamide (6a)

To a stirred solution of amine **8** (20 mg, 0.05 mmol) in dry DMF (1.5 mL), commercially available (*S*)‐5‐oxopyrrolidine‐2‐carboxylic acid **9a** (8 mg, 0.06 mmol), HATU (21.8 mg, 0.06 mmol) and DIPEA (42 μL, 0.24 mmol) were added under inert atmosphere at 0 °C. The resulting mixture was stirred at 0–23 °C for 12 h. After completion of the reaction, the solvent was evaporated under reduced pressure and the crude mixture was diluted with water and extracted with CH_2_Cl_2_ (3 times). The combined organic layer was dried over Na_2_SO_4_ and concentrated under reduced pressure. The resulting crude mixture was purified by column chromatography (2–5% MeOH in CH_2_Cl_2_) to yield compound **6a** as an amorphous orange solid (16 mg, 62%).): 1H NMR (500 MHz, DMSO) *δ* 11.04 (s, 1H), 9.27 (s, 1H), 8.59 (d, *J* = 7.9 Hz, 1H), 8.18 (d, *J* = 1.7 Hz, 1H), 7.91 (s, 1H), 7.68–7.60 (m, 2H), 7.46–7.39 (m, 2H), 7.16–7.09 (m, 2H), 6.92 (d, *J* = 8.1Hz, 1H), 5.16 (p, *J* = 7.3 Hz, 1H), 4.20 (dd, *J* = 8.7, 4.5 Hz, 1H), 2.44–2.32 (m, 1H), 2.27–2.09 (m, 8H), 2.00 (ddt, *J* = 15.0, 9.7, 5.2 Hz, 1H), 1.48 (d, *J* = 7.0 Hz, 3H); ^13^C NMR (126 MHz, DMSO) *δ* 178.0, 172.4, 170.2, 166.4, 141.7, 140.8, 132.4, 128.5 (d, *J* = 8.0 Hz, C_F‐C_), 127.9, 127.6, 126.2, 125.9, 124.9, 124.7, 121.7, 117.7, 115.3 (d, *J* = 20.9 Hz, C_F‐C_), 113.1, 109.1, 56.3, 48.3, 29.8, 26.2, 22.7, 12.1, 9.7; HRMS (ESI): *m*/*z* calcd for C_29_H_29_FN_5_O_4_ [M + H]^+^ 530.2204 found 530.2186.

##### (Z)‐3‐((3,5‐Dimethyl‐4‐((R)‐5‐Oxopyrrolidine‐2‐Carboxamido)‐1H‐Pyrrol‐2‐Yl)Methylene)‐N‐((R)‐1‐(4‐Fluorophenyl)Ethyl)‐2‐Oxoindoline‐5‐Carboxamide (6b)

Following the procedure as described for compound **6a**, amine **8** (20 mg, 0.05 mmol), and (*R*)‐5‐oxopyrrolidine‐2‐carboxylic acid **9b** (8 mg, 0.06 mmol) provided compound **6b** as an orange solid (16 mg, 65%); *R*_*f*_  = 0.20 (MeOH : CH_2_Cl_2_ = 1:9);^1^H NMR (400 MHz, DMSO) *δ* 11.08 (s, 1H), 9.31 (s, 1H), 8.63 (d, *J* = 8.0 Hz, 1H), 8.21 (d, *J* = 1.7 Hz, 1H), 7.94 (s, 1H), 7.72–7.57 (m, 2H), 7.42 (ddd, *J* = 10.1, 6.1, 3.3 Hz, 2H), 7.28–7.05 (m, 2H), 6.91 (d, *J* = 8.1 Hz, 1H), 5.16 (p, *J* = 7.1 Hz, 1H), 4.19 (dd, *J* = 8.7, 4.5 Hz, 1H), 2.45–2.30 (m, 1H), 2.30–2.08 (m, 8H), 1.99 (ddt, *J* = 14.0, 9.7, 5.1 Hz, 1H), 1.47 (d, *J* = 7.0 Hz, 3H);^13^C NMR (126 MHz, DMSO) *δ* 177.8, 172.3, 170.2, 166.3, 161.4 (d, *J* = 241.8 Hz, C_F‐C_), 141.7, 140.8, 132.4, 128.7, 128.5 (d, *J* = 8.0 Hz, C_F‐C_), 128.0, 127.6, 126.20, 125.9, 124.9, 124.7, 121.8, 120.5, 117.8, 115.3 (d, *J* = 21.0 Hz, C_F‐C_), 113.2, 109.1, 56.3, 48.3, 29.8, 26.3, 22.7, 12.1, 9.7; HRMS (ESI): *m*/*z* calcd for C_29_H_29_FN_5_O_4_ [M + H]^+^ 530.2204 found 530.2195.

##### Tert‐Butyl(R)‐2‐((5‐(((Z)‐5‐(((R)‐1‐(4‐Fluorophenyl)Ethyl)Carbamoyl)‐2‐Oxoindolin‐3‐Ylidene)Methyl)‐2,4‐Dimethyl‐1H‐Pyrrol‐3‐Yl)Carbamoyl)Pyrrolidine‐1‐Carboxylate(6c)

Following the procedure as described for compound **6a**, amine **8** (20 mg, 0.05 mmol), and (*tert*‐butoxycarbonyl)‐*D*‐proline **9c** (13 mg, 0.06 mmol) provided compound **6c** as a brick red solid (17 mg, 58%); *R*_*f*_  = 0.50 (MeOH : CH_2_Cl_2_ = 0.5:9.5); ^1^H NMR (500 MHz, DMSO) *δ* 11.04 (s, 1H), 9.15 (s, 1H), 8.58 (d, *J* = 8.0 Hz, 1H), 8.18 (s, 1H), 7.83–7.58 (m, 2H), 7.53–7.35 (m, 2 H), 7.13 (t, *J* = 8.9 Hz, 2H), 6.91 (d, *J* = 8.1 Hz, 1H), 5.17 (p, *J* = 7.2 Hz, 1H), 4.23 (ddd, *J* = 18.7, 8.5, 3.1 Hz, 1H), 3.43 (t, *J* = 9.0 Hz, 1H), 2.29–2.15 (m, 7H), 1.85 (dq, *J* = 30.0, 7.4 Hz, 3H), 1.48 (d, *J* = 7.1 Hz, 3H), 1.39 (d, *J* = 15.9 Hz, 9H); ^13^C NMR (126 MHz, DMSO) *δ* 172.2, 172.1, 170.1, 166.4, 161.4 (d, *J* = 241.7 Hz, C_F‐C_), 154.1, 153.8, 141.7, 140.7, 132.7, 132.3, 128.5 (d, *J* = 7.9 Hz, C_F‐C_), 128.0, 127.8, 127.5, 126.1, 125.9, 124.9, 124.6, 122.2, 122.1, 117.8, 115.3 (d, *J* = 21.1 Hz, C_F‐C_), 113.0, 112.9, 109.1, 79.1, 79.0, 60.2, 48.3, 47.1, 47.0, 32.2, 30.8, 28.6, 24.5, 23.6, 22.7, 12.2, 12.0, 9.7, 9.6; HRMS (ESI): *m*/*z* calcd for C_34_H_39_FN_5_O_5_ [M + H]^+^ 616.2935 found 616.2921.

##### Tert‐Butyl(S)‐2‐((5‐(((Z)‐5‐(((R)‐1‐(4‐Fluorophenyl)Ethyl)Carbamoyl)‐2‐Oxoindolin‐3‐Ylidene)Methyl)‐2,4‐Dimethyl‐1H‐Pyrrol‐3‐Yl)Carbamoyl)Pyrrolidine‐1‐Carboxylate(6d)

Following the procedure as described for compound **6a**, amine **8** (20 mg, 0.05 mmol) and (*tert*‐butoxycarbonyl)‐*L*‐proline **9d** (13 mg, 0.06 mmol) provided compound **6d** as a red solid (21 mg, 72%); *R*_*f*_  = 0.5 (MeOH : CH_2_Cl_2_ = 0.5:9.5);^1^H NMR (500 MHz, DMSO) *δ* 11.05 (s, 1 H), 9.13 (d, *J* = 34.0 Hz, 1H), 8.59 (d, *J* = 8.0 Hz, 1H), 8.18 (s, 1 H), 7.93–7.51 (m, 2 H), 7.54–7.34 (m, 2H), 7.13 (dd, *J* = 10.1, 7.7 Hz, 2H), 6.91 (d, *J* = 8.1 Hz, 1H), 5.16 (h, *J* = 7.0 Hz, 1H), 4.23 (ddd, *J* = 18.9, 8.4, 3.1 Hz, 1H), 3.43 (t, *J* = 8.9 Hz, 1H), 2.33–2.13 (m, 7 H), 1.97–1.74 (m, 4H), 1.48 (d, *J* = 7.1 Hz, 3H), 1.39 (d, *J* = 16.1 Hz, 9H);^13^C NMR (126 MHz, DMSO) *δ* 172.2, 172.1, 170.1, 166.4, 161.4 (d, *J* = 241.8 Hz, C_F‐C_), 154.1, 153.8, 141.7, 140.7, 132.7, 132.3, 128.3 (d, *J* = 8.1 Hz, C_F‐C_), 128.0, 127.8, 127.5, 126.1, 125.9, 124.9, 124.6, 122.2, 122.1, 117.8, 115.3 (d, *J* = 21.1 Hz, C_F‐C_), 113.0, 112.9, 109.1, 79.1, 79.0, 60.2, 48.3, 47.1, 47.0, 32.2, 30.8, 28.6, 24.5, 23.6, 22.7, 12.2, 12.0, 9.7, 9.6; HRMS (ESI): *m*/*z* calcd for C_34_H_39_FN_5_O_5_ [M + H]^+^ 616.2935 found 616.2914.

##### (Z)‐3‐((4‐((R)‐1‐Acetylpyrrolidine‐2‐Carboxamido)‐3,5‐Dimethyl‐1H‐Pyrrol‐2‐Yl)Methylene)‐N‐((R)‐1‐(4‐Fluorophenyl)Ethyl)‐2‐Oxoindoline‐5‐Carboxamide (6e)

Following the procedure as described for compound **6a**, amine **8** (20 mg, 0.05 mmol) and acetyl‐*D*‐proline **9e** (9 mg, 0.06 mmol) provided compound **6e** as an golden yellow solid (14 mg, 52%); *R*_*f*_  = 0.30 (MeOH : CH_2_Cl_2_ = 1:9);^1^H NMR (500 MHz, DMSO) *δ* 11.04 (s, 1 H), 9.02 (s, 1H), 8.59 (d, *J* = 7.9 Hz, ^1^H), 8.18 (dd, *J* = 5.4, 1.6 Hz, 1H), 7.97–7.55 (m, 2H), 7.55–7.36 (m, 2H), 7.31–7.08 (m, 2H), 6.91 (dd, *J* = 8.2, 1.8 Hz, 1H), 5.17 (p, *J* = 7.2 Hz, 1H), 4.42 (ddd, *J* = 77.5, 8.5, 3.0 Hz, ^1^H), 3.82–3.59 (m, ^1^H), 3.48 (dq, *J* = 11.9, 7.4 Hz, 1H), 3.44–3.33 (m, 1H), 2.15 (d, *J* = 5.1 Hz, 6H), 2.00 (s, 3 H), 1.96–1.77 (m, 3H), 1.48 (d, *J* = 7.0 Hz, 3 H);^13^C NMR (126 MHz, DMSO) *δ* 171.9, 171.8, 170.2, 169.1, 166.4, 160.5, 141.7, 132.9, 128.5 (d, *J* = 7.9 Hz, C_F‐C_), 128.0, 126.1, 125.9, 124.9, 124.7, 122.2, 117.9, 117.7, 115.3 (d, *J* = 21.1 Hz, C_F‐C_), 112.9, 109.1, 61.0, 60.0, 48.3, 48.0, 46.8, 32.8, 30.5, 24.9, 23.1, 22.9, 22.7, 22.6, 12.0, 9.6; HRMS (ESI): *m*/*z* calcd for C_31_H_33_FN_5_O_4_ [M + H]^+^ 558.2517 found 558.2495.

##### ((Z)‐3‐((4‐((S)‐1‐Acetylpyrrolidine‐2‐Carboxamido)‐3,5‐Dimethyl‐1H‐Pyrrol‐2‐Yl)Methylene)‐N‐((R)‐1‐(4‐Fluorophenyl)Ethyl)‐2‐Oxoindoline‐5‐Carboxamide (6f)

Following the procedure as described for compound **6a**, amine **8** (20 mg, 0.05 mmol) and acetyl‐*L*‐proline **9f** (9 mg, 0.06 mmol) provided compound **6f** as golden yellow solid (15 mg, 58%); *R*_*f*_  = 0.30 (MeOH : CH_2_Cl_2_ = 1:9); ^1^H NMR (500 MHz, DMSO) *δ* 11.04 (s, ^1^H), 9.02 (s, 1H), 8.59 (d, *J* = 7.9 Hz, 1H), 8.18 (dd, *J* = 5.4, 1.7 Hz, ^1^H), 7.83–7.56 (m, 2H), 7.55–7.34 (m, 2H), 7.13 (t, *J* = 8.9 Hz, 2H), 6.91 (dd, *J* = 8.1, 1.9 Hz, 1H), 5.17 (p, *J* = 7.2 Hz, 1H), 4.49–4.22 (m, 1H), 3.62 (td, *J* = 8.6, 4.2 Hz, ^1^H), 3.52–3.34 (m, 2H), 2.25–2.06 (m, 6H), 2.00 (s, 3H), 1.95–1.81 (m, 3H), 1.48 (d, *J* = 7.0 Hz, 3H); ^13^C NMR (126 MHz, DMSO) *δ* 171.8, 170.2, 169.1, 166.4, 161.4 (d, *J* = 241.6 Hz, C_F‐C_), 141.7, 140.7, 132.9, 128.5 (d, *J* = 8.2 Hz, C_F‐C_), 127.9, 127.6, 126.1, 125.9, 124.9, 124.7, 122.1, 121.7, 117.9, 117.7, 115.3 (d, *J* = 21.1 Hz, C_F‐C_), 112.9, 109.1, 61.0, 60.0, 48.3, 48.0, 32.8, 30.5, 24.9, 23.1, 22.9, 22.7, 22.6, 12.0, 9.6; HRMS (ESI): *m*/*z* calcd for C_31_H_33_FN_5_O_4_ [M + H]^+^ 558.2517 found 558.2502.

##### (Z)‐3‐((3,5‐Dimethyl‐4‐((R)‐Pyrrolidine‐2‐Carboxamido)‐1H‐Pyrrol‐2‐yl)Methylene)‐N‐((R)‐1‐(4‐Fluorophenyl)Ethyl)‐2‐Oxoindoline‐5‐Carboxamide (6g)

To a stirred solution of **6c** (15 mg, 0.024 mmol) in dry DCM (1.5 mL), added TFA (0.048 mmol) under inert atmosphere at 0 °C. The resulting mixture was stirred at 0–23 °C for 2 h. After completion of the reaction, the solvent was evaporated under reduced pressure and washed with diethyl ether several times. The solid was dried in vacuum to yield compound **6g** as an amorphous brick red solid (12 mg, 92%).^1^H NMR (500 MHz, DMSO) *δ* 11.08 (s, 1H), 9.78 (s, 1H), 9.36 (s, 1H), 8.94–8.36 (m, 2H), 8.20 (s, 1H), 7.92–7.57 (m, 2H), 7.42 (dd, *J* = 8.5, 5.5 Hz, 2H), 7.13 (t, *J* = 8.7 Hz, 2H), 6.92 (d, *J* = 8.1 Hz, 1H), 5.17 (t, *J* = 7.2 Hz, 1H), 4.38 (s, 1H), 3.26 (d, *J* = 13.7 Hz, 2H), 2.43 (t, *J* = 7.0 Hz, 1H), 2.20 (d, *J* = 12.4 Hz, 6H), 1.98 (ddt, *J* = 19.5, 12.9, 6.8 Hz, 3H), 1.48 (d, *J* = 7.0 Hz, 3H), 1.24 (q, *J* = 7.0 Hz, 1H); ^13^C NMR (126 MHz, DMSO) *δ* 170.2, 168.0, 166.3, 161.4 (d, *J* = 241.8 Hz, C_F‐C_), 141.7, 140.9, 131.8, 128.5 (d, *J* = 8.1 Hz, C_F‐C_), 128.1, 127.1, 126.3, 125.8, 125.0, 124.7, 120.7, 118.0, 115.3 (d, *J* = 21.1 Hz, C_F‐C_), 113.8, 109.1, 59.8, 48.3, 46.1, 30.4, 24.0, 22.7, 12.1, 9.6; HRMS (ESI): *m*/*z* calcd for C_29_H_31_FN_5_O_3_ [M + H]^+^ 516.2411 found 516.2402.

##### (Z)‐3‐((3,5‐Dimethyl‐4‐((S)‐pyrrolidine‐2‐carboxamido)‐1H‐pyrrol‐2‐yl)methylene)‐N‐((R)‐1‐(4‐fluorophenyl)ethyl)‐2‐oxoindoline‐5‐carboxamide (6 h)

Following the general procedure for compound **6g**, compound **6d** (15 mg, 0.024 mmol) provided compound **6h** as a red solid (11 mg, 90%); *R*_*f*_  = 0.2 (MeOH : CH_2_Cl_2_ = 1:9); ^1^H NMR (500 MHz, DMSO) *δ* 11.09 (s, 1H), 9.80 (s, 1H), 9.39 (d, *J* = 37.0 Hz, 1H), 8.61 (d, *J* = 8.0 Hz, 1H), 8.20 (s, 1H), 7.87–7.61 (m, 2 H), 7.54–7.35 (m, 2H), 7.13 (t, *J* = 8.7 Hz, 2H), 6.92 (d, *J* = 8.1 Hz, 1H), 5.41–4.78 (m, 1H), 4.39 (t, *J* = 7.5 Hz, 1H), 3.33–3.20 (m, 2H), 2.44 (d, *J* = 12.3 Hz, 1H), 2.20 (d, *J* = 12.4 Hz, 6H), 1.98 (ddt, *J* = 19.5, 12.9, 6.9 Hz, 3H), 1.48 (d, *J* = 7.0 Hz, 3H); ^13^C NMR (126 MHz, DMSO) *δ* 170.2, 168.0, 166.3, 161.4 (d, *J* = 241.8 Hz, C_F‐C_), 141.7, 140.9, 131.8, 128.5 (d, *J* = 8.0 Hz, C_F‐C_), 128.1, 127.1, 126.3, 125.8, 125.0, 124.7, 120.7, 118.0, 115.3 (d, *J* = 21.1 Hz, C_F‐C_), 113.8, 109.1, 59.8, 48.3, 46.1, 30.4, 24.0, 22.7, 12.1, 9.6; HRMS (ESI): *m*/*z* calcd for C_29_H_31_FN_5_O_3_ [M + H]^+^ 516.2411 found 516.2395.

##### Tert‐Butyl(S)‐2‐((5‐(((Z)‐5‐(((R)‐1‐(4‐Fluorophenyl)Ethyl)Carbamoyl)‐2‐Oxoindolin‐3‐yl‐ Idene)Methyl)‐2,4‐Dimethyl‐1H‐Pyrrol‐3‐Yl)Carbamoyl)‐2,5‐Dihydro‐1H‐Pyrrole‐1‐Carboxylate (6i)

Following the procedure as described for compound **6a**, amine **8** (20 mg, 0.05 mmol) and (*S*)‐1‐(*tert*‐butoxycarbonyl)‐2,5‐dihydro‐1 H‐pyrrole‐2‐carboxylic acid **9g** (13 mg, 0.06 mmol) provided compound **6i** as an amorphous an orange solid (21 mg, 74%); *R*_*f*_  = 0.45 (MeOH:CH_2_Cl_2_ = 0.5:9.5); ^1^H NMR (500 MHz, DMSO) *δ* 11.04 (s, 1H), 9.22 (d, *J* = 43.7 Hz, 1H), 8.58 (d, *J* = 7.9 Hz, 1H), 8.18 (s, 1H), 7.88–7.58 (m, 2H), 7.51–7.33 (m, 2H), 7.25–7.07 (m, 2H), 6.91 (d, *J* = 8.1 Hz, 1H), 6.04 (ddt, *J* = 12.2, 6.4, 3.2 Hz, 1H), 5.87 (ddd, *J* = 6.5, 3.9, 2.0 Hz, 1H), 5.17 (p, *J* = 7.2 Hz, 1H), 5.09–4.89 (m, 1H), 4.57–4.01 (m, 2H), 2.17 (dd, *J* = 10.8, 7.3 Hz, 6H), 1.48 (d, *J* = 7.0 Hz, 3H), 1.41 (d, *J* = 16.4 Hz, 9H); ^13^C NMR (126 MHz, DMSO) *δ* 170.2, 169.4, 166.4, 161.4 (d, *J* = 242.3 Hz, C_F‐C_), 153.6, 153.5, 141.7, 140.8, 132.7, 132.2, 129.1, 128.7, 128.5 (d, *J* = 8.1 Hz, C_F‐C_), 128.0, 127.4, 127.1, 126.6, 126.1, 125.9, 124.9, 124.6, 121.9, 117.8, 115.3 (d, *J* = 21.1 Hz, C_F‐C_), 113.2, 113.0, 109.1, 79.4, 68.2, 68.1, 54.1, 48.3, 28.5, 28.5, 22.7, 12.2, 12.0, 9.7, 9.6; HRMS (ESI): *m*/*z* calcd for C_34_H_37_FN_5_O_5_ [M + H]^+^ 614.2779 found 614.2757.

##### (Z)‐3‐((4‐((S)‐2,5‐Dihydro‐1H‐Pyrrole‐2‐Carboxamido)‐3,5‐Dimethyl‐1H‐Pyrrol‐2‐Yl)Methylene)‐N‐((R)‐1‐(4‐Fluorophenyl)Ethyl)‐2‐Oxoindoline‐5‐Carboxamide (6j)

Following the general procedure for compound **6g**, compound **6i** (15 mg, 0.024 mmol) provided compound **6j** as an orange solid (11 mg, 88%); *R*_*f*_  = 0.2 (MeOH : CH_2_Cl_2_ = 1:9); ^1^H NMR (500 MHz, DMSO) *δ* 11.09 (s, 1H), 9.94 (s, 1 H), 8.98 (s, 1H), 8.60 (d, *J* = 7.9 Hz, 1H), 8.19 (d, *J* = 1.7 Hz, 1H), 7.80–7.59 (m, 2H), 7.59–7.25 (m, 2H), 7.22–7.06 (m, 2H), 6.92 (d, *J* = 8.1 Hz, 1H), 6.11 (d, *J* = 2.2 Hz, 2H), 5.36–5.02 (m, 2H), 4.28–3.95 (m, 2H), 2.20 (d, *J* = 13.2 Hz, 6H), 1.47 (d, *J* = 7.1 Hz, 3H); ^13^C NMR (126 MHz, DMSO) *δ* 170.2, 166.3, 166.0, 161.4 (d, *J* = 242.2 Hz, C_F‐C_), 141.7, 140.9, 131.8, 128.5 (d, *J* = 8.1 Hz, C_F‐C_), 128.0, 127.0, 126.3, 125.8, 125.8, 125.0, 124.6, 120.6, 118.0, 115.3 (d, *J* = 21.1 Hz, C_F‐C_), 113.9, 109.2, 66.9, 53.1, 48., 22.73, 12.0, 9.6; HRMS (ESI): *m*/*z* calcd for C_29_H_29_FN_5_O_3_ [M + H]^+^ 514.2254 found 514.2245.

##### Tert‐Butyl(R)‐3‐((5‐(((Z)‐5‐(((R)‐1‐(4‐Fluorophenyl)Ethyl)Carbamoyl)‐2‐Oxoindolin‐3‐Ylidene)Methyl)‐2,4‐Dimethyl‐1H‐Pyrrol‐3‐Yl)Carbamoyl)Pyrrolidine‐1‐Carboxylate(6k)

Following the procedure as described for compound **6a**, amine **8** (20 mg, 0.05 mmol) and (*R*)‐1‐(*tert*‐butoxycarbonyl)pyrrolidine‐3‐carboxylic acid **9h** (13 mg, 0.06 mmol) provided compound **6k** as a brick red solid (19 mg, 65%); *R*_*f*_  = 0.53 (MeOH : CH_2_Cl_2_ = 0.5:9.5); ^1^H NMR (500 MHz, DMSO) *δ* 11.07 (s, 1H), 9.32 (s, 1H), 8.69 (d, *J* = 8.0 Hz, 1H), 8.30 (s, 1H), 7.84–7.57 (m, 2H), 7.44 (dd, *J* = 8.4, 5.5 Hz, 2H), 7.12 (t, *J* = 8.8 Hz, 2H), 6.91 (d, *J* = 8.1 Hz, 1H), 5.17 (p, *J* = 7.3 Hz, 1H), 3.51 (dd, *J* = 10.6, 7.9 Hz, 1H), 3.37 (m, 2H), 3.24 (m, 1H), 3.20–3.12 (m, 1H), 2.17 (d, *J* = 2.3 Hz, 6H), 2.12 (q, *J* = 5.5 Hz, 1H), 2.01 (d, *J* = 5.9 Hz, 1H), 1.49 (d, *J* = 7.1 Hz, 3H), 1.39 (s, 9 H); ^13^C NMR (126 MHz, DMSO) *δ* 170.2, 166.4, 161.4 (d, *J* = 241.8 Hz, C_F‐C_), 153.9, 141.7, 140.7, 132.4, 128.5 (d, *J* = 8.0 Hz, C_F‐C_), 127.9, 127.5, 126.1, 125.9, 124.9, 124.7, 122.1, 117.7, 115.3 (d, *J* = 21.1 Hz, C_F‐C_), 113.0, 109.1, 78.8, 48.9, 48.3, 45.9, 45.7, 43.8, 42.9, 29.8, 29.1, 28.6, 22.7, 12.1, 9.7; HRMS (ESI): *m*/*z* calcd for C_34_H_39_FN_5_O_5_ [M + H]^+^ 616.2935 found 616.2893.

##### Tert‐Butyl(S)‐3‐((5‐(((Z)‐5‐(((R)‐1‐(4‐Fluorophenyl)Ethyl)Carbamoyl)‐2‐Oxoindolin‐3‐Ylidene)Methyl)‐2,4‐Dimethyl‐1H‐Pyrrol‐3‐Yl)Carbamoyl)Pyrrolidine‐1‐Carboxylate(6l)

Following the procedure as described for compound **6a**, amine **8** (20 mg, 0.05 mmol) and (*S*)‐1‐(*tert*‐butoxycarbonyl)pyrrolidine‐3‐carboxylic acid **9i** (13 mg, 0.06 mmol) provided compound **6l** as a brick red solid (21 mg, 71%); *R*_*f*_  = 0.53 (MeOH : CH_2_Cl_2_ = 0.5:9.5); ^1^H NMR (500 MHz, DMSO) *δ* 11.04 (s, 1H), 9.22 (s, 1H), 8.58 (d, *J* = 7.9 Hz, 1H), 8.30–8.08 (m, 1H), 7.75–7.60 (m, 2H), 7.52–7.37 (m, 2H), 7.27–7.09 (m, 2H), 6.91 (d, *J* = 8.1 Hz, 1H), 5.17 (p, *J* = 7.2 Hz, 1H), 3.52 (dd, *J* = 10.6, 7.9 Hz, 1H), 3.48–3.34 (m, 2H), 3.25 (t, *J* = 9.5 Hz, 1H), 3.13 (dt, *J* = 15.1, 7.5 Hz, 1H), 2.17 (d, *J* = 7.5 Hz, 6H), 2.10 (td, *J* = 7.4, 5.1 Hz, 1H), 2.05–1.96 (m, 1H), 1.48 (d, *J* = 7.1 Hz, 3H), 1.39 (s, 9H); ^13^C NMR (126 MHz, DMSO) *δ* 171.9, 170.2, 166.4, 161.4 (d, *J* = 241.4 Hz, C_F‐C_), 153.8, 141.7, 140.7, 132.4, 128.5 (d, *J* = 7.9 Hz, C_F‐C_), 128.0, 127.5, 126.1, 125.9, 124.9, 124.6, 122.1, 117.8, 115.3 (d, *J* = 21.1 Hz, C_F‐C_), 113.1, 109.1, 78.7, 48.9, 48.3, 45.9, 45.7, 43.8, 42.9, 29.8, 29.1, 28.6, 22.7, 12.1, 9.7; HRMS (ESI): *m*/*z* calcd for C_34_H_39_FN_5_O_5_ [M + H]^+^ 616.2935 found 616.2927.

##### Tert‐Butyl(R)‐2‐((5‐(((Z)‐5‐(((R)‐1‐(4‐Fluorophenyl)Ethyl)Carbamoyl)‐2‐Oxoindolin‐3‐Ylidene)Methyl)‐2,4‐Dimethyl‐1H‐Pyrrol‐3‐Yl)Carbamoyl)Piperidine‐1‐Carboxylate(6m)

Following the procedure as described for compound **6a**, amine **8** (20 mg, 0.05 mmol) and (*R*)‐1‐(*tert*‐butoxycarbonyl)piperidine‐2‐carboxylic acid **9j** (14 mg, 0.06 mmol) provided compound **6m** as a dark red solid (19 mg, 65%); *R*_*f*_  = 0.5 (MeOH : CH_2_Cl_2_ = 0.5:9.5); ^1^H NMR (500 MHz, DMSO) *δ* 11.04 (s, 1H), 9.11 (s, 1H), 8.58 (d, *J* = 8.0 Hz, 1H), 8.18 (d, *J* = 1.7 Hz, 1H), 7.90–7.58 (m, 2H), 7.53–7.34 (m, 2H), 7.13 (dd, *J* = 10.0, 7.8 Hz, 2H), 6.91 (d, *J* = 8.1 Hz, 1H), 5.17 (p, *J* = 7.2 Hz, 1H), 4.69 (d, *J* = 25.4Hz, 1 H), 3.81 (d, *J* = 12.8 Hz, 1H), 3.16 (d, *J* = 51.9 Hz, 1H), 2.17 (d, *J* = 7.5 Hz, 6H), 1.62 (d, *J* = 10.4 Hz, 3H), 1.48 (d, *J* = 7.0 Hz, 3H), 1.39 (s, 9H), 1.34–1.17 (m, 2H); ^13^C NMR (126 MHz, DMSO) *δ* 170.2, 166.4, 161.4 (d, *J* = 242.9 Hz, C_F‐C_), 141.7, 140.8, 132.5, 128.5 (d, *J* = 8.1 Hz, C_F‐C_), 128.0, 127.7, 126.1, 125.9, 124.9, 124.7, 122.2, 117.8, 115.3 (d, *J* = 21.1 Hz, C_F‐C_), 113.1, 109.1, 79.2, 48.3, 41.5, 28.5, 28.2, 24.7, 22.7, 20.2, 12.1, 9.7; HRMS (ESI): *m*/*z* calcd for C_35_H_41_FN_5_O_5_ [M + H]^+^ 630.3092 found 630.3074.

##### Tert‐Butyl(S)‐2‐((5‐(((Z)‐5‐(((R)‐1‐(4‐Fluorophenyl)Ethyl)Carbamoyl)‐2‐Oxoindolin‐3‐Ylidene)Methyl)‐2,4‐Dimethyl‐1H‐Pyrrol‐3‐Yl)Carbamoyl)Piperidine‐1‐Carboxylate(6n)

Following the procedure as described for compound **6a**, amine **8** (20 mg, 0.05 mmol) and (*S*)‐1‐(*tert*‐butoxycarbonyl)piperidine‐2‐carboxylic acid **9k** (14 mg, 0.06 mmol) provided compound **6n** as a red solid (18 mg, 60%); *R*_*f*_  = 0.6 (MeOH : CH_2_Cl_2_ = 0.5:9.5); ^1^H NMR (500 MHz, DMSO) *δ* 11.04 (s, 1H), 9.11 (s, 1H), 8.59 (d, *J* = 7.9 Hz, 1H), 8.18 (d, *J* = 1.7 Hz, 1H), 7.84–7.56 (m, 2H), 7.52–7.39 (m, 2H), 7.29–7.08 (m, 2H), 6.91 (d, *J* = 8.1 Hz, 1H), 5.17 (p, *J* = 7.2 Hz, 1H), 4.69 (d, *J* = 25.5 Hz, 1H), 3.81 (d, *J* = 12.9 Hz, 1H), 3.16 (d, *J* = 51.0 Hz, 1H), 2.17 (d, *J* = 7.4 Hz, 6H), 1.62 (d, *J* = 10.4 Hz, 3H), 1.48 (d, *J* = 7.0 Hz, 3H), 1.39 (s, 9H), 1.36–1.26 (m, 2H); ^13^C NMR (126 MHz, DMSO) *δ* 170.2, 166.4, 161.4 (d, *J* = 241.8 Hz, C_F‐C_), 141.7, 140.8, 132.5, 128.5 (d, *J* = 8.1 Hz, C_F‐C_), 128.0, 127.7, 126.1, 125.9, 124.9, 124.7, 122.2, 117.8, 115.3 (d, *J* = 21.1 Hz, C_F‐C_), 113.0, 109.1, 79.2, 48.3, 28.5, 24.7, 22.7, 20.2, 12.1, 9.7; HRMS (ESI): *m*/*z* calcd for C_35_H_41_FN_5_O_5_ [M + H]^+^ 630.3092 found 630.3072.

##### Tert‐Butyl(S)‐3‐((5‐(((Z)‐5‐(((R)‐1‐(4‐Fluorophenyl)Ethyl)Carbamoyl)‐2‐Oxoindolin‐3‐Ylidene)Methyl)‐2,4‐Dimethyl‐1H‐Pyrrol‐3‐Yl)Carbamoyl)Piperidine‐1‐Carboxylate(6o)

Following the procedure as described for compound **6a**, amine **8** (20 mg, 0.05 mmol) and (*S*)‐1‐(*tert*‐butoxycarbonyl)piperidine‐3‐carboxylic acid **9L** (14 mg, 0.06 mmol) provided compound **6o** as brick red solid (21 mg, 72%); *R*_*f*_  = 0.32 (MeOH : CH_2_Cl_2_ = 0.5:9.5); ^1^H NMR (500 MHz, DMSO) *δ* 11.04 (s, 1H), 9.16 (s, 1H), 8.58 (d, *J* = 8.0 Hz, 1H), 8.18 (d, *J* = 2.0 Hz, 1H), 7.80–7.57 (m, 2H), 7.42 (dd, *J* = 8.6, 5.7 Hz, 2H), 7.13 (t, *J* = 8.9 Hz, 2H), 6.91 (d, *J* = 8.1 Hz, 1H), 5.17 (p, *J* = 7.2 Hz, 1H), 4.01 (q, *J* = 7.1 Hz, 1H), 3.87 (d, *J* = 13.1 Hz, 1H), 3.01–2.53 (m, 5H), 2.16 (d, *J* = 7.6 Hz, 6H), 1.89–1.55 (m, 2H), 1.48 (d, *J* = 7.1 Hz, 3H), 1.40 (s, 9H); ^13^C NMR (126 MHz, DMSO) *δ* 172.7, 171.7, 170.2, 166.4, 161.4 (d, *J* = 241.3 Hz, C_F‐C_), 154.3, 141.7, 140.7, 132.4, 128.5 (d, *J* = 8.1 Hz, C_F‐C_), 127.9, 127.5, 126.1, 125.9, 124.9, 124.6, 122.1, 117.7, 115.3 (d, *J* = 21.0 Hz, C_F‐C_), 113.0, 109.1, 79.2, 48.3, 43.4, 42.6, 28.5, 24.7, 22.7, 12.1, 9.6; HRMS (ESI): *m*/*z* calcd for C_35_H_41_FN_5_O_5_ [M + H]^+^ 630.3092 found 630.3075.

##### Tert‐Butyl(R)‐3‐((5‐(((Z)‐5‐(((R)‐1‐(4‐Fluorophenyl)Ethyl)Carbamoyl)‐2‐Oxoindolin‐3‐Ylidene)Methyl)‐2,4‐Dimethyl‐1H‐Pyrrol‐3‐Yl)Carbamoyl)Piperidine‐1‐Carboxylate(6p)

Following the procedure as described for compound **6a**, amine **8** (20 mg, 0.05 mmol) and (*R*)‐1‐(*tert*‐butoxycarbonyl)piperidine‐3‐carboxylic acid **9m** (14 mg, 0.06 mmol) provided compound **6p** as a brick red solid (22 mg, 75%); *R*_*f*_  = 0.43 (MeOH : CH_2_Cl_2_ = 0.5:9.5); ^1^H NMR (500 MHz, DMSO) *δ* 9.16 (s, 1H), 8.58 (d, *J* = 8.0 Hz, 1H), 8.18 (d, *J* = 1.7 Hz, 1H), 7.76–7.58 (m, 2H), 7.47–7.38 (m, 2H), 7.23–7.09 (m, 2H), 6.91 (d, *J* = 8.1 Hz, 1H), 5.17 (p, *J* = 7.4 Hz, 1H), 4.15–3.98 (m, 1 H), 3.87 (d, *J* = 13.2 Hz, 1H), 2.80 (d, *J* = 56.5 Hz, 2H), 2.44 (dq, *J* = 11.0, 3.5 Hz, 1H), 2.16 (d, *J* = 7.5 Hz, 6H), 1.94 (s, 1H), 1.78–1.55 (m, 2H), 1.48 (d, *J* = 7.1 Hz, 3H), 1.40 (s, 9H); ^13^C NMR (126 MHz, DMSO) *δ* 172.6, 170.1, 166.4, 161.4 (d, *J* = 241.9 Hz, C_F‐C_), 154.3, 141.7, 140.7, 132.4, 128.5 (d, *J* = 8.1 Hz, C_F‐C_), 128.0, 127.6, 126.1, 125.9, 124.9, 124.6, 122.1, 117.7, 115.3 (d, *J* = 21.1 Hz, C_F‐C_), 113.0, 109.1, 79.2, 48.3, 42.65, 28.5, 28.2, 24.7, 22.7, 12.1, 9.7; HRMS (ESI): *m*/*z* calcd for C_35_H_40_FN_5_O_5_Na [M + Na]^+^ 652.2911 found 652.2909.

##### (Z)‐3‐((3,5‐Dimethyl‐4‐((S)‐Piperidine‐3‐Carboxamido)‐1H‐Pyrrol‐2‐Yl)Methylene)‐N‐((R)‐1‐(4‐Fluorophenyl)Ethyl)‐2‐Oxoindoline‐5‐Carboxamide (6q)

Following the general procedure for compound **6g**, compound **6o** (15 mg, 0.023 mmol) provided compound **6q** as red solid (10 mg, 82%); *R*_*f*_  = 0.2 (MeOH : CH_2_Cl_2_ = 1:9); ^1^H NMR (400 MHz, DMSO) *δ* 11.05 (s, 1H), 9.36 (s, 1H), 8.59 (d, *J* = 7.9 Hz, 1H), 8.17 (d, *J* = 1.6 Hz, 1H), 7.77–7.56 (m, 2H), 7.52–7.37 (m, 2H), 7.26–7.07 (m, 2H), 6.91 (d, *J* = 8.1 Hz, 1H), 5.16 (p, *J* = 7.1 Hz, 1H), 3.02 (d, *J* = 61.7 Hz, 4H), 2.72–2.53 (m, 2H), 2.17 (d, *J* = 8.2 Hz, 6H), 1.77 (d, *J* = 48.3 Hz, 3H), 1.47 (d, *J* = 7.0 Hz, 3H); ^13^C NMR (126 MHz, DMSO) *δ* 171.8, 170.2, 166.4, 161.4 (d, *J* = 241.3 Hz, C_F‐C_), 141.7, 140.8, 132.2, 128.5 (d, *J* = 8.2 Hz, C_F‐C_), 128.0, 127.4, 126.1, 125.9, 124.9, 124.7, 121.6, 117.9, 115.3 (d, *J* = 21.0 Hz, C_F‐C_), 113.3, 109.1, 48.3, 44.9, 43.6, 39.1, 27.0, 22.7, 21.6, 12.1, 9.7; HRMS (ESI): *m*/*z* calcd for C_30_H_33_FN_5_O_3_ [M + H]^+^ 530.2567 found 530.2558.

##### (Z)‐3‐((3,5‐Dimethyl‐4‐((R)‐Piperidine‐3‐Carboxamido)‐1H‐Pyrrol‐2‐Yl)Methylene)‐N‐((R)‐1‐(4‐Fluorophenyl)Ethyl)‐2‐Oxoindoline‐5‐Carboxamide (6r)

Following the general procedure for compound **6g**, compound **6p** (15 mg, 0.023 mmol) provided compound **6r** as a brick red solid (11 mg, 86%); *R*_*f*_  = 0.2 (MeOH : CH_2_Cl_2_ = 1:9); ^1^H NMR (500 MHz, DMSO) *δ* 11.06 (s, 1H), 9.37 (s, 1H), 8.86–8.42 (m, 3H), 8.18 (s, 1H), 7.86–7.59 (m, 2H), 7.42 (dd, *J* = 8.5, 5.5 Hz, 2H), 7.13 (t, *J* = 8.7 Hz, 2H), 6.91 (d, *J* = 8.1 Hz, 1H), 5.17 (p, *J* = 7.3 Hz, 1H), 3.30 (d, *J* = 12.7 Hz, 1H), 3.22–3.02 (m, 2H), 2.88 (d, *J* = 48.3 Hz, 3H), 2.17 (d, *J* = 10.5 Hz, 6H), 2.06 (d, *J* = 9.3 Hz, 1H), 1.83 (s, 1H), 1.71 (t, *J* = 9.0 Hz, 2H), 1.48 (d, *J* = 7.1 Hz, 3H); ^13^C NMR (126 MHz, DMSO) *δ* 171.7, 170.2, 166.4, 161.4 (d, *J* = 241.8 Hz, C_F‐C_), 141.7, 140.8, 132.2, 128.5 (d, *J* = 8.2 Hz, C_F‐C_), 128.0, 127.4, 126.1, 125.9, 124.9, 124.7, 121.6, 117.9, 115.3 (d, *J* = 21.0 Hz, C_F‐C_), 113.3, 109.1, 48.3, 44.8, 43.5, 39.0, 27.0, 22.7, 21.5, 12.1, 9.6; HRMS (ESI): *m*/*z* calcd for C_30_H_33_FN_5_O_3_ [M + H]^+^ 530.2567 found 530.2561.

##### Tert‐Butyl(R)‐3‐((5‐(((Z)‐5‐(((R)‐1‐(4‐Fluorophenyl)Ethyl)Carbamoyl)‐2‐Oxoindolin‐3‐Ylidene)Methyl)‐2,4‐Dimethyl‐1H‐Pyrrol‐3‐Yl)Carbamoyl)Piperazine‐1‐Carboxylate(6s)

Following the procedure as described for compound **6a**, amine **8** (20 mg, 0.05 mmol) and (*R*)‐4‐(*tert*‐butoxycarbonyl)piperazine‐2‐carboxylic acid **9n** (14 mg, 0.06 mmol) provided compound **6s** as dark red solid (19 mg, 64%); *R*_*f*_  = 0.3 (MeOH : CH_2_Cl_2_ = 1:9); ^1^H NMR (500 MHz, DMSO) *δ* 11.04 (s, 1H), 9.13 (s, 1H), 8.58 (d, *J* = 7.9 Hz, 1H), 8.18 (d, *J* = 1.8 Hz, 1H), 7.94–7.57 (m, 2H), 7.53–7.36 (m, 2H), 7.22–7.04 (m, 2H), 6.91 (d, *J* = 8.1 Hz, 1H), 5.17 (p, *J* = 7.2 Hz, 1H), 3.96 (d, *J* = 63.8 Hz, 2H), 3.60 (d, *J* = 12.4 Hz, 1H), 3.34 (dd, *J* = 9.6, 3.9 Hz, 1H), 3.13–2.84 (m, 2H), 2.60 (t, *J* = 11.9 Hz, 1H), 2.17 (d, *J* = 8.7 Hz, 6H), 1.48 (d, *J* = 7.1 Hz, 3H), 1.40 (s, 9H); ^13^C NMR (126 MHz, DMSO) *δ* 170.5, 170.2, 166.4, 161.4 (d, *J* = 241.8 Hz, C_F‐C_), 154.3, 141.7, 140.7, 132.5, 128.5 (d, *J* = 8.1 Hz, C_F‐C_), 128.0, 127.7, 126.1, 125.9, 124.9, 124.7, 121.9, 117.8, 115.3 (d, *J* = 20.8 Hz, C_F‐C_), 113.0, 109.1, 79.3, 58.2, 48.3, 44.1, 28.5, 22.7, 12.1, 9.7; HRMS (ESI): *m*/*z* calcd for C_34_H_40_FN_6_O_5_ [M + H]^+^ 631.3044 found 631.3044.

##### Tert‐Butyl(S)‐3‐((5‐(((Z)‐5‐(((R)‐1‐(4‐Fluorophenyl)Ethyl)Carbamoyl)‐2‐Oxoindolin‐3‐Ylidene)Methyl)‐2,4‐Dimethyl‐1H‐Pyrrol‐3‐Yl)Carbamoyl)Piperazine‐1‐Carboxylate(6t)

Following the procedure as described for compound **6a**, amine **8** (20 mg, 0.05 mmol) and (*S*)‐4‐(*tert*‐butoxycarbonyl)piperazine‐2‐carboxylic acid **9o** (14 mg, 0.06 mmol) provided compound **6t** as a brick red solid (18 mg, 60%); *R*_*f*_  = 0.3 (MeOH : CH_2_Cl_2_ = 1:9); ^1^H NMR (500 MHz, DMSO) *δ* 11.04 (s, 1H), 9.14 (s, 1H), 8.58 (d, *J* = 7.9 Hz, 1H), 8.18 (s, 1H), 7.95–7.58 (m, 2H), 7.43 (dd, *J* = 8.4, 5.5 Hz, 2H), 7.13 (t, *J* = 8.7 Hz, 2H), 6.91 (d, *J* = 8.1 Hz, 1H), 5.17 (q, *J* = 7.5 Hz, 1H), 3.90 (s, 1H), 3.60 (d, *J* = 12.5 Hz, 1H), 3.53–3.32 (m, 2H), 3.16–2.76 (m, 3H), 2.73–2.55 (m, 1H), 2.17 (d, *J* = 8.6 Hz, 6H), 1.48 (d, *J* = 7.1 Hz, 3H), 1.40 (s, 9H); ^13^C NMR (126 MHz, DMSO) *δ* 170.5, 170.2, 166.4, 161.4 (d, *J* = 241.5 Hz, C_F‐C_), 154.3, 141.7, 140.8, 132.5, 128.5 (d, *J* = 8.0 Hz, C_F‐C_), 128.0, 127.7, 126.17, 125.9, 124.9, 124.6, 121.9, 117.8, 115.3 (d, *J* = 21.1 Hz, C_F‐C_), 113.0, 109.1, 79.4, 58.2, 48.3, 44.1, 28.5, 22.7, 12.1, 9.7; HRMS (ESI): *m*/*z* calcd for C_34_H_40_FN_6_O_5_ [M + H]^+^ 631.3044 found 631.3044.

##### (Z)‐3‐((3,5‐Dimethyl‐4‐((R)‐Piperazine‐2‐Carboxamido)‐1 H‐Pyrrol‐2‐Yl)Methylene)‐N‐((R)‐1‐(4‐Fluorophenyl)Ethyl)‐2‐Oxoindoline‐5‐Carboxamide (6u)

Following the general procedure for compound **6g**, compound **6s** (15 mg, 0.023 mmol) provided compound **6u** as red solid (10 mg, 81%); *R*_*f*_  = 0.1 (MeOH : CH_2_Cl_2_ = 1:9); ^1^H NMR (500 MHz, DMSO) *δ* 11.08 (s, 1H), 9.76 (s, 1H), 8.60 (d, *J* = 7.6 Hz, 1H), 8.19 (s, 1H), 7.93–7.56 (m, 2H), 7.42 (dd, *J* = 8.4, 5.5 Hz, 2H), 7.13 (t, *J* = 8.7 Hz, 2H), 6.92 (d, *J* = 8.1 Hz, 1H), 5.18 (q, *J* = 7.4 Hz, 1H), 4.15 (d, *J* = 42.3 Hz, 1H), 3.66 (s, 1H), 3.30 (d, *J* = 15.9 Hz, 2H), 3.12 (dd, *J* = 25.8, 13.4 Hz, 3H), 2.20 (d, *J* = 12.5 Hz, 6H), 1.48 (d, *J* = 7.1 Hz, 3H); ^13^C NMR (126 MHz, DMSO) *δ* 170.2, 166.3, 161.4 (d, *J* = 245.3 Hz, C_F‐C_), 141.7, 140.9, 132.0, 128.5 (d, *J* = 8.1 Hz, C_F‐C_), 128.1, 127.2, 126.3, 125.8, 125.0, 124.7, 120.7, 118.0, 115.3 (d, *J* = 20.8 Hz, C_F‐C_), 113.7, 109.1, 54.7, 48.3, 43.9, 22.7, 12.1, 9.7; HRMS (ESI): *m*/*z* calcd for C_29_H_32_FN_6_O_3_ [M + H]^+^ 531.2520 found 531.2511.

##### (Z)‐3‐((3,5‐Dimethyl‐4‐((S)‐Piperazine‐2‐Carboxamido)‐1H‐Pyrrol‐2‐Yl)Methylene)‐N‐((R)‐1‐(4‐Fluorophenyl)Ethyl)‐2‐Oxoindoline‐5‐Carboxamide (6v)

Following the general procedure for compound **6g**, compound **6t** (15 mg, 0.023 mmol) provided compound **6v** as a brick red solid (11 mg, 89%); *R*_*f*_  = 0.1 (MeOH : CH_2_Cl_2_ = 1:9); ^1^H NMR (500 MHz, DMSO) *δ* 11.09 (s, 1H), 9.92 (s, 1H), 9.25 (s, 1H), 8.60 (d, *J* = 7.9 Hz, 1H), 8.20 (s, 1H), 7.77–7.59 (m, 2H), 7.55–7.36 (m, 2H), 7.13 (t, *J* = 8.6 Hz, 2H), 6.92 (d, *J* = 8.1 Hz, 1H), 5.60–5.05 (m, 1H), 4.27 (s, 1H), 3.81 (d, *J* = 13.1 Hz, 1H), 3.41 (s, 2H), 3.18 (dt, *J* = 27.4, 13.2 Hz, 3H), 2.20 (d, *J* = 13.2 Hz, 6H), 1.48 (d, *J* = 7.1 Hz, 3H); ^13^C NMR (126 MHz, DMSO) *δ* 170.2, 166.3, 161.4 (d, *J* = 241.6 Hz, C_F‐C_), 141.7, 140.9, 131.9, 128.5 (d, *J* = 8.0 Hz, C_F‐C_), 128.1, 127.1, 126.3, 125.8, 125.0, 124.6, 120.4, 118.0, 115.3 (d, *J* = 21.1 Hz, C_F‐C_), 113.9, 109.1, 54.3, 48.3, 43.3, 42.1, 41.3, 22.7, 12.0, 9.6; HRMS (ESI): *m*/*z* calcd for C_29_H_32_FN_6_O_3_ [M + H]^+^ 531.2520 found 531.2511.

##### Determination of X‐Ray Structures of GRK5 and Inhibitor 6t Complex

Human GRK5 (residues 1‐590) D311N mutant were expressed in *E. coli* Rosetta (*DE3*) and purified as described previously.^[^ ^37^ ^]^ GRK5_D311N_ was mixed with MgCl_2_ and Sgv to achieve a final concentration of 118 μM GRK5, 354 μM Sgv, and 118 μM MgCl_2_. Crystals were obtained in a hanging drop vapor diffusion apparatus over a condition of 220 mM potassium citrate tribasic and 20% polyethylene glycol 3350 at 4 °C. Crystals were allowed to grow for one week until stable sizes were obtained and were transferred to a new hanging drop tray in a 4 μL suspended drop containing 20% PEG3350, 10% glycerol, and inhibitor at a final concentration of 1 mM (4% dimethylsulfoxide [DMSO[ in final mixture) as described previously.^[^ ^37^, ^38^ ^]^ Finally, individual crystals were directly frozen by flash freezing on nylon loops in liquid nitrogen.

Diffraction data were collected at the Brookhaven National Laboratory on NSLS‐II 17‐ID‐1(AMX) at a wavelength of 0.9201 Å, with 1° angle per frame for a total of 180 frames. Automated‐processed data from Fast Data Processing (Fast DP) was used to achieve molecular replacement in PHENIX Phaser‐MR, using as a search model the GRK5 structure from PDB entry 8UAP. Refinements were performed using phenix.refine alternating with manual building and fitting in COOT.^[^ ^45^ ^]^ The final models were validated with MolProbity prior to deposition along with structure factors in the Protein Data Bank.^[^ ^46^ ^]^ Atomic figures were created with Pymol.^[^ ^47^ ^]^ Crystallographic statistics are reported in Supplemental Table 1. Coordinates for inhibitor **6t** (GRL‐098‐22) were deposited in the Protein Data Bank^[^ ^46^ ^]^ with accession code 9BRK.

## Conflict of Interest

The authors declare no conflict of interest.

## Supporting information

Supplementary Material

## Data Availability

The data that support the findings of this study are available in the supplementary material of this article.
