# Supplementary material for: Potent, Selective, and Drug‐Like G Protein‐Coupled Receptor Kinase 5 and 6 Inhibitors: Design, Synthesis, and X‐Ray Structural Studies
Source: ChemMedChem. 2025 Sep 29;20(20):e202500257. doi: 10.1002/cmdc.202500257 (PMC12530842; doi:10.1002/cmdc.202500257)
Supplement: Supplementary file 1 — Supplementary Material [file CMDC-20-e202500257-s001.pdf]

Supporting Information

Potent, Selective, and Drug-like G Protein-coupled Receptor Kinase 5 and 6 Inhibitors: Design, Synthesis, and X-ray Structural Studies

Arun K. Ghosh, Ranjith Kumar Gadi, Yueyi Chen, Sandali Piladuwa Gamage, Kathryn P. McCauley, and John J. G. Tesmer

Table of Contents

1. General Methods……………………………………………………………………………..S1
2. Protein expression, purification and kinetics…………………………………………………..S2
3. Diffraction data and refinement statistics……….…………………………...……………………S3
4. Supplementary Figure S1. Docked Figure of **6t**-GRK2 and **6t**-GRK5 X-ray structure………S4
5. Copies of ^{1}H and ^{13}C NMR spectra of key intermediates and inhibitors……………..…..S5-S26

General Methods.

All reagents and solvents were purchased from commercial sources and used without purification. NMR spectra were recorded with a 300, 400 or 500 MHz spectrometer for ^{1}H NMR, 100 or 125 MHz for ^{13}C NMR spectroscopy. Chemical shifts are reported relative to the residual signals of tetramethyl silane in DMSO-*d6* for ^{1}H and ^{13}C NMR spectroscopy. Multiplicities are reported as follows: singlet (s), doublet (d), doublet of doublets (dd), doublet of triplets (dt), triplet (t), quartet (q), multiplet (m). double-doublet (dd), double-double-doublet (ddd), triple-doublet (td), triple-triplet (tt), double-quartet (dq) etc. HRMS were recorded by using TOF, Qt of, Orbitrap mass spectrometer. Column chromatography was performed with silica gel (100–200 mesh) as the stationary phase. All reactions were monitored by using TLC. Characterizations of new compounds were further established by using HRMS.

Protein expression and purification.

Human GRK5 (residues 1-590) wild-type and D311N mutant were expressed in *E. coli* Rosetta (*DE3*) and purified via nickel-nitrilotriacetic acid affinity chromatography, followed by tandem columns consisting of a HiTrap Q HP anion exchange chromatography column and a HiTrap SP HP cation exchange chromatography column, and finished by Superdex 200 Increase size-exclusion chromatography, as described previously [1]. Human GRK6 (with T10A and mutations that eliminate palmitoylation sites) was expressed in Sf9 insect cells [2] and was purified with the same protocol for GRK5 [1]. Human GRK2 was purified as described previously [3].

Kinase inhibition Assays.

Inhibition assays were conducted in a reaction mixture containing 20 mM HEPES pH 7.0, 2 mM $MgCl_2$, and 0.025% n-dodecyl-β-D-maltoside. Prior to reaction initiation, GRK5 or GRK6 at 50 nM was mixed with porcine brain tubulin (PurSolutions) at a concentration of 500 nM. The assay was started with a 30-second incubation of inhibitors, followed by the addition of 5 μM ATP supplemented with radioactive [γ-^{32}P]-ATP at 60 Ci/mmol (PerkinElmer Life Sciences). Reactions proceeded for 5 minutes at room temperature and were terminated by adding 4X SDS loading buffer. The reaction mixtures were then separated via SDS-PAGE, dried, and exposed using a phosphor imaging screen. Quantification was performed using a Personal Molecular Imager and Quantity One 1-D Analysis Software. Analogous inhibition assays were conducted on purified GRK2 with a tubulin concentration of 1 μM. Data analysis was performed using GraphPad Prism, and IC_{50} values were calculated based on three-parameter dose-dependent curves (with a Hill coefficient fixed at 1) plotting phosphate transfer against inhibitor concentration. At least three replicates were conducted to determine the mean and standard deviation of IC_{50} values as published previously [4,5].

Determination of X-ray structures of GRK5 and inhibitor 6t complex:

Human GRK5 (residues 1-590) D311N mutant were expressed in *E. coli* Rosetta (*DE3*) and purified as described previously [4]. $GRK5_{D311N}$ was mixed with $MgCl_2$ and Sgv to achieve a final concentration of 118 μM GRK5, 354 μM Sgv, and 118 μM $MgCl_2$. Crystals were obtained in a hanging drop vapor diffusion apparatus over a condition of 220 mM potassium citrate tribasic and 20% polyethylene glycol 3350 at 4 °C, allowed to grow for one week until stable sizes were obtained and were transferred to a new hanging drop tray in a 4 μl suspended drop containing 20% PEG3350, 10% glycerol, and inhibitor at a final concentration of 1 mM (4% DMSO in final mixture) as described previously [4,5]. Finally, individual crystals were directly frozen by flash freezing on nylon loops in liquid nitrogen.

Diffraction data were collected at the Brookhaven National Laboratory on NSLS-II 17-ID-1(AMX) at a wavelength of 0.9201 Å, with 1° angle per frame for a total of 180 frames. Automated-processed data from Fast Data Processing (Fast DP) was used to achieve molecular replacement in PHENIX Phaser-MR , using as a search model the GRK5 structure from PDB entry 8UAP. Refinements were performed using phenix.refine alternating with manual building and fitting in COOT. (6) The final models were validated with MolProbity prior to deposition along with structure factors in the Protein Data Bank. Atomic figures were created with Pymol (7). Crystallographic statistics are reported in Supplemental Table 1. Coordinates for inhibitor **6t** (GRL-098-22) were deposited in the Protein Data Bank, with accession code 9BRK.

References:

1. Y. Chen, A. Sonawane, R. Manda, R. K. Gadi, J. J. G. Tesmer, A. K. Ghosh. Development of a new class of potent and highly selective G protein-coupled receptor kinase 5 inhibitors and structural insight from crystal structures of inhibitor complexes. *Eur. J. Med. Chem.* **2024***, 264*, 115931.
2. D. T. Lodowski, V. M. Tesmer, J. L. Benovic, J. J. Tesmer. The structure of G protein-coupled receptor kinase (GRK)-6 defines a second lineage of GRKs. *J. Biol. Chem.* **2006**, *281*,16785-16793.

3. R. A. Bouley, Z. Y. Weinberg, H. V. Waldschmidt, Y. C. Yen, S. D. Larsen, M. A. Puthenveedu, J. J. G. Tesmer. A New Paroxetine-Based GRK2 Inhibitor Reduces Internalization of the μ-Opioid Receptor. *Mol Pharmacol.* **2020**, *97*, 392-401.
4. Y. Chen, A. Sonawane, R. Manda, R. K. Gadi, J. J. G. Tesmer, A. K. Ghosh, "Development of a new class of potent and highly selective G protein-coupled receptor kinase 5 inhibitors and structural insight from crystal structures of inhibitor complexes" *Eur. J. Med. Chem.* **2024**, *264*, 115931.
5. A. K. Ghosh, Y. Chen, R. K. Gadi, A. Sonawane, S. P. Gamage, J. J. G. Tesmer, "Design, synthesis, and X-ray structural studies of a series of highly potent, selective, and drug-like G protein-coupled receptor kinase 5 inhibitors" *Eur J Med Chem.* **2025**, *282,* 117024.
6. P. Emsley, K. Cowtan. Coot: model-building tools for molecular graphics." *Acta Crystallogr.* **2004**, *60*, 2126-2132.
7. L. Schrödinger, W. DeLano, "The PyMOL Molecular Graphics System", 2020, Version 2.0 Schrödinger, LLC.. Retrieved from http://www.pymol.org/pymol).

Supplemental Table 1. Data collection and refinement statistics for the GRK5·6t complex

**Data Collection**	**GRK5-6t (GRL098-22)**
X-ray source	NSLS-II 17-ID-1
Wavelength	0.9201
Resolution range	24.65 – 2.7 (2.77 – 2.7)
Space group	$P\,4_1\,2_1\,2$
Unit cell	137.52, 137.52, 70.75
Total reflections	236294 (18079)
Unique reflections	17757 (1324)
Redundancy	13.3 (13.7)
Completeness (%)	99.19 (100.0)
Mean $\langle I \rangle / \langle \sigma \rangle$	13.77 (2.95)
R_{merge}	0.1799 (0.946)
R_{meas}	0.1873 (0.9828)
R_{pim}	0.05155 (0.2635)
Reflections used in refinement	18997 (1438)
**Refinement**	
R-work	0.2175
R-free	0.2755
Protein atoms	4092
Ligand atoms	84
RMSD (bonds)*	0.37
RMSD (angles)*	4.08
Ramachandran favored (%)	94.39
Ramachandran outliers (%)	0.8
Clashscore	4.85
**B-factor**	
Average B-factor	80.48
macromolecules	80.16
ligands	129.13
**PDB Code**	9BRK

*RMSD, root mean square deviation

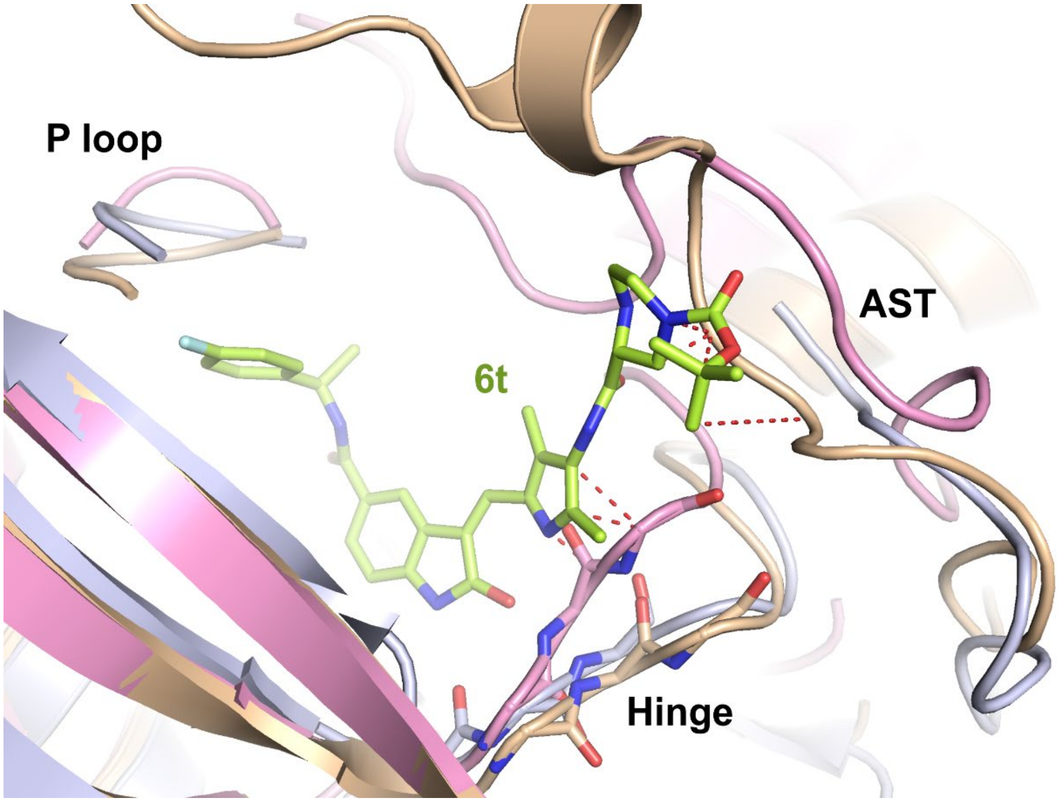

Figure S1. Crystal structure of **6t** in complex with GRK5 (PDB entry 9BRK): Overlay of the GRK2 kinase domain, closed conformation (8JPB) shown in yellow and open conformation (PDB entry 5UKM) shown in pink, with GRK5 structure (light blue) in complex with inhibitor **6t**. Steric clashes of the inhibitor with the GRK2 AST loop are indicated by red dashed lines.

IV. Copies of ^{1}H & ^{13}C NMR spectra of compounds 6a-v:

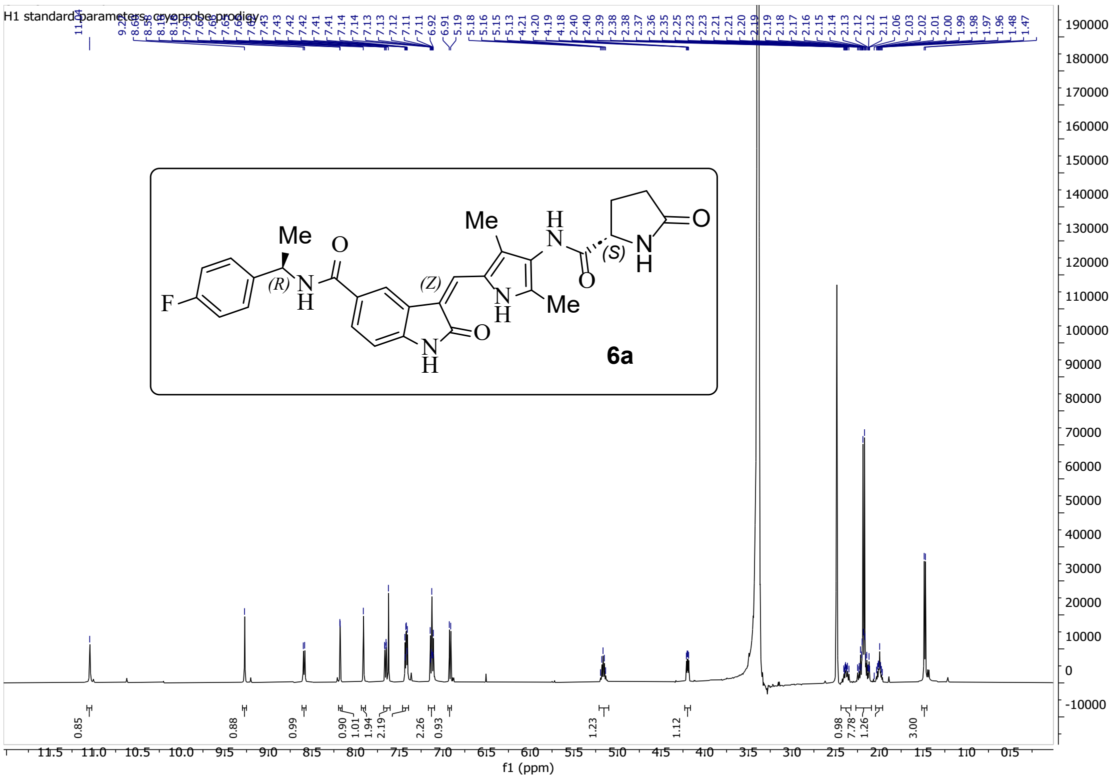

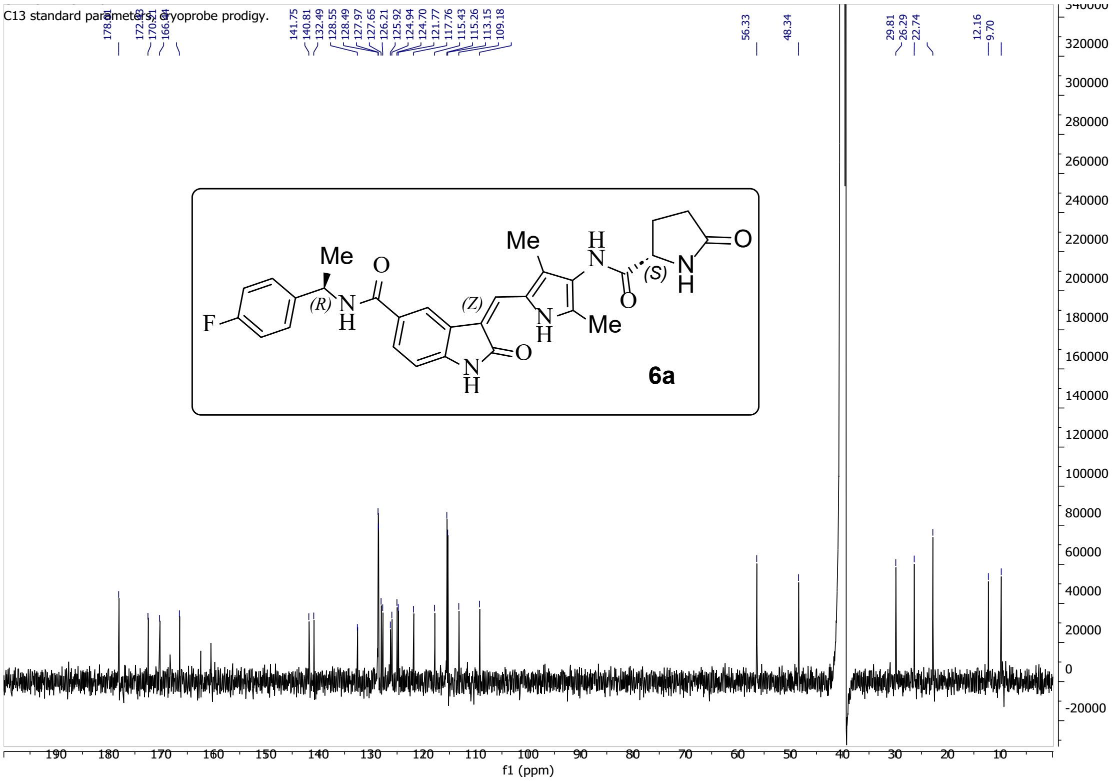

^{1}H & ^{13}C NMR Spectra of Compound 6a (DMSO-d_6)

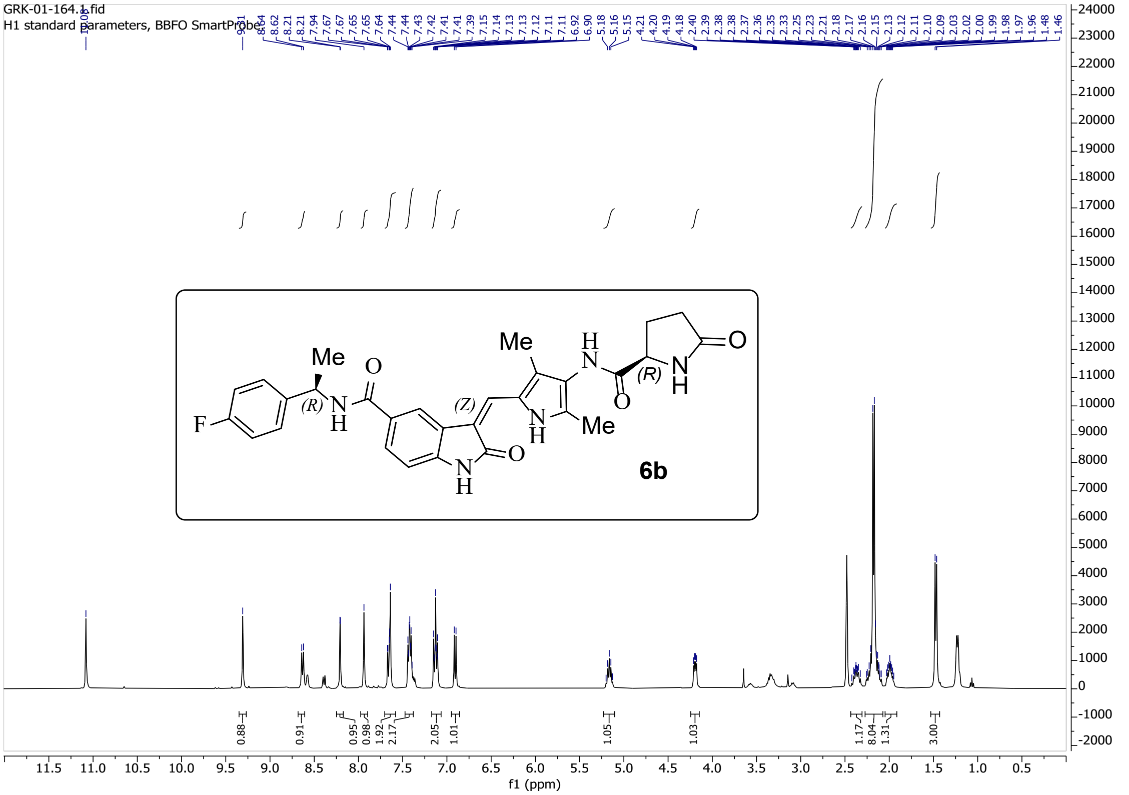

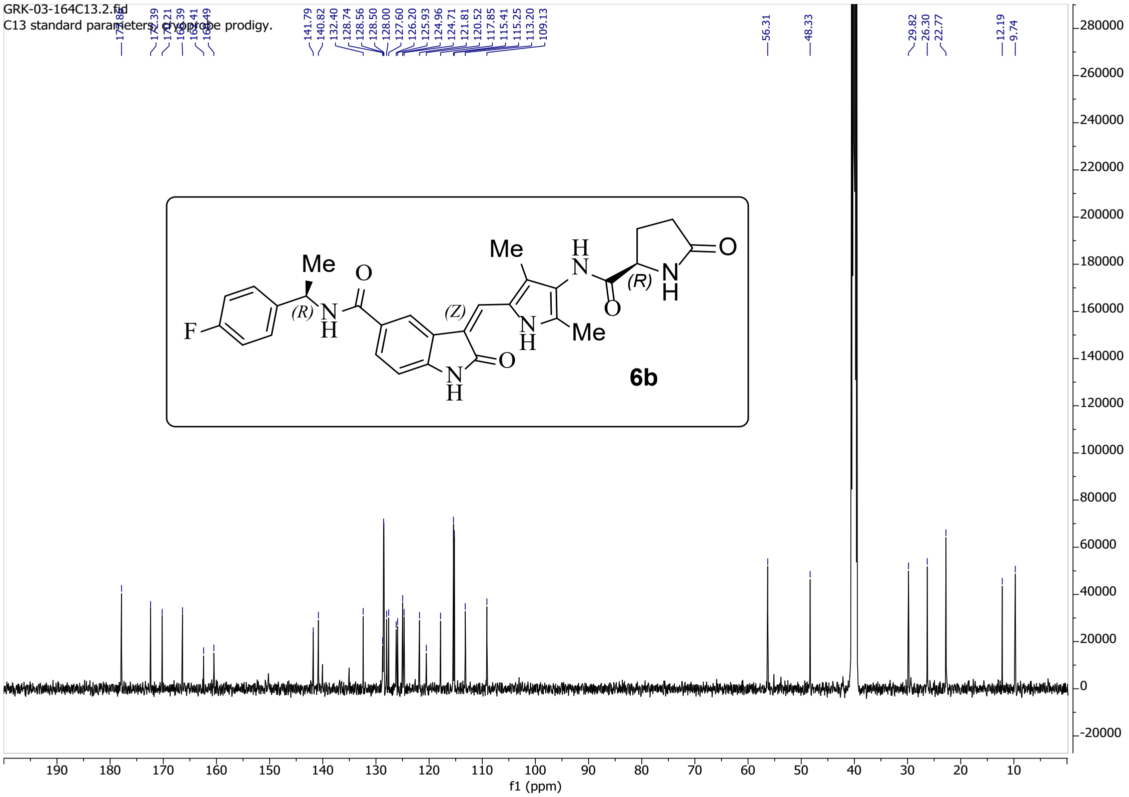

^{1}H & ^{13}C NMR Spectra of Compound 6b (DMSO-d_6)

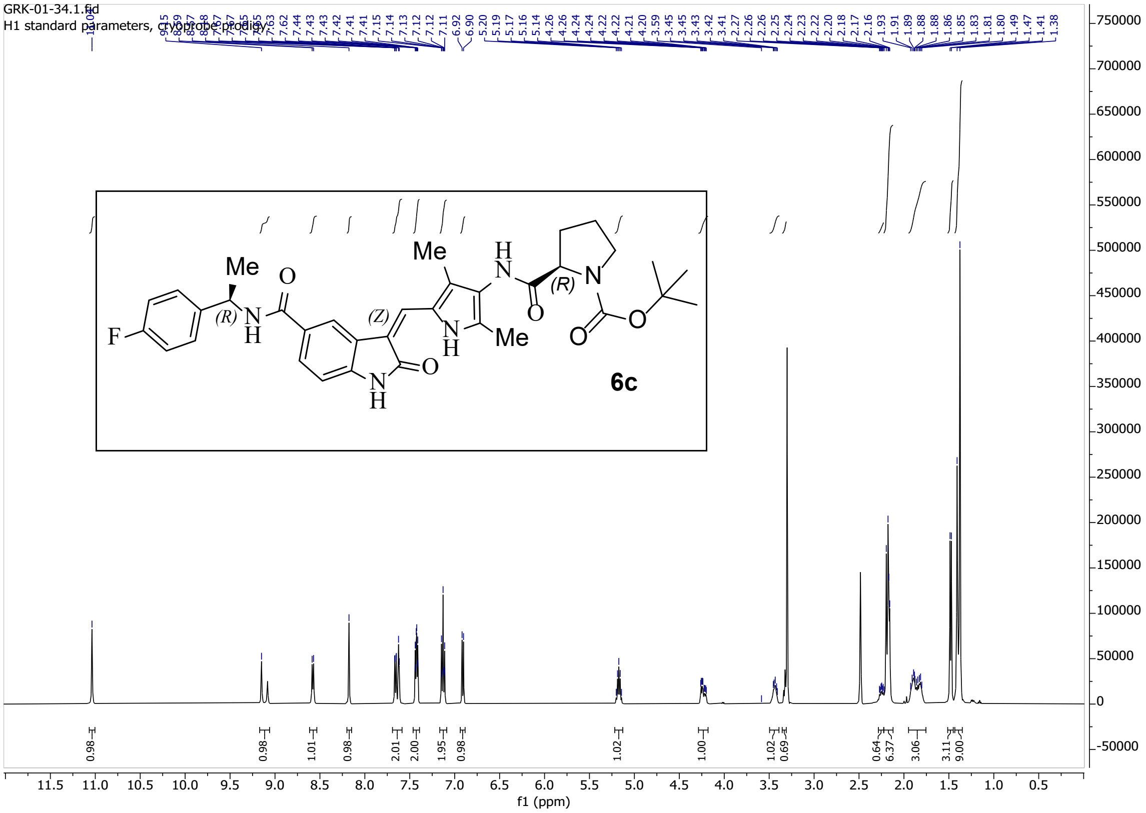

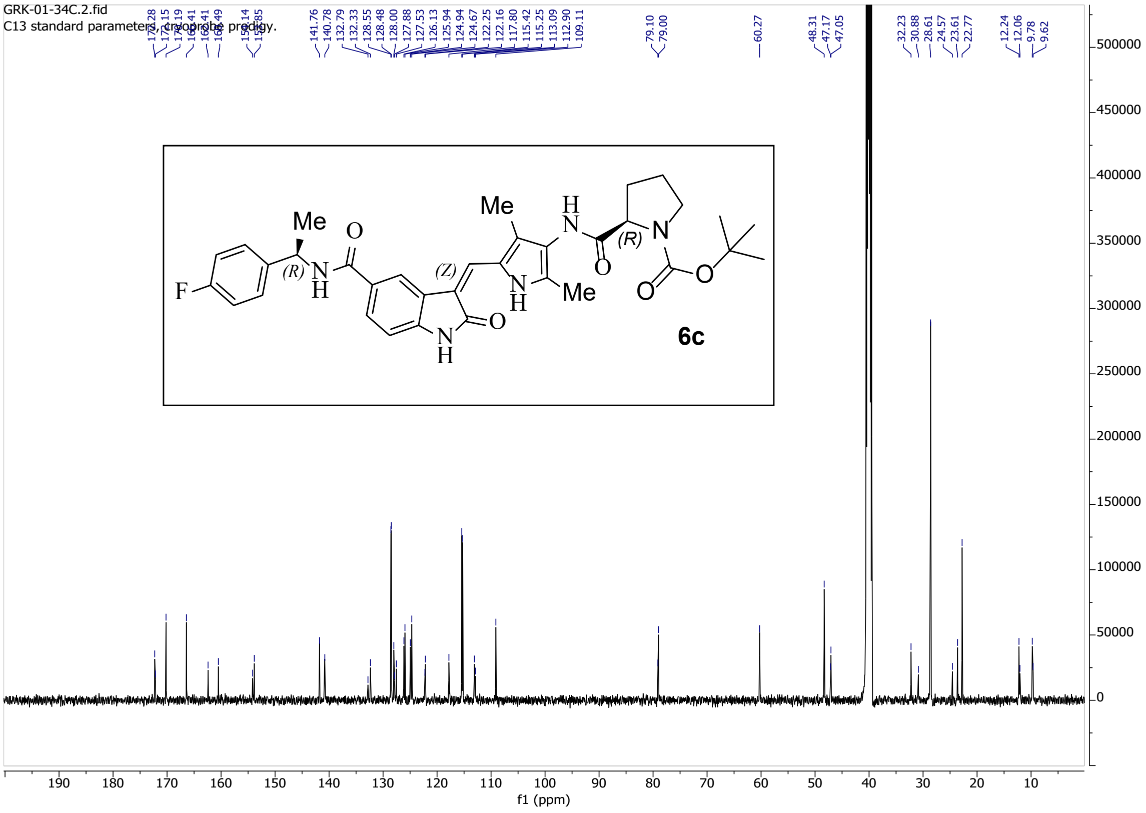

^{1}H & ^{13}C NMR Spectra of Compound 6c (DMSO-d_6)

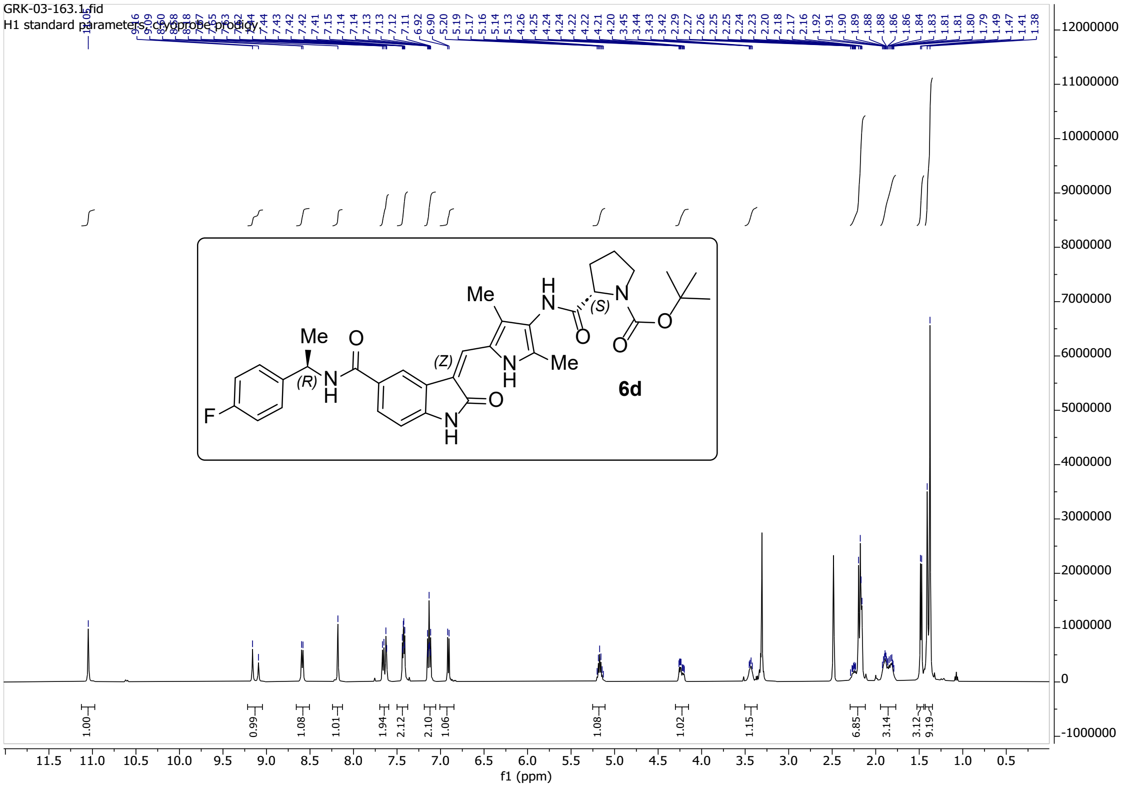

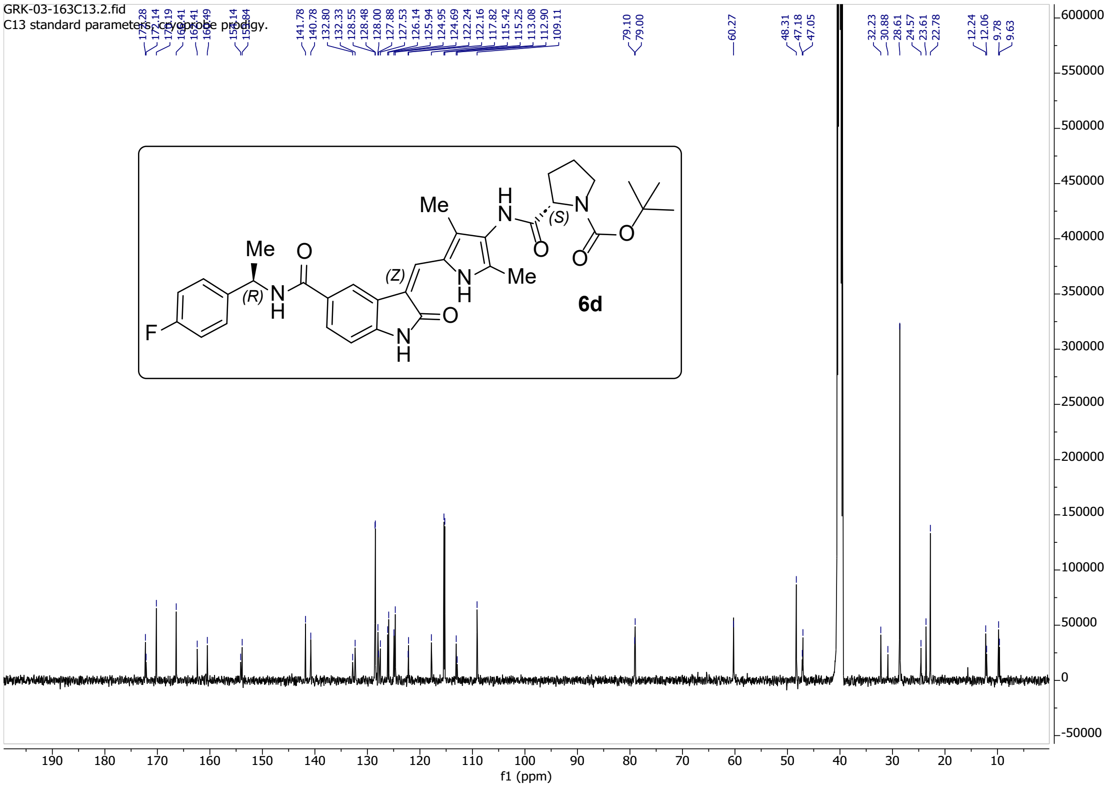

^{1}H & ^{13}C NMR Spectra of Compound 6d (DMSO-d$_6$)

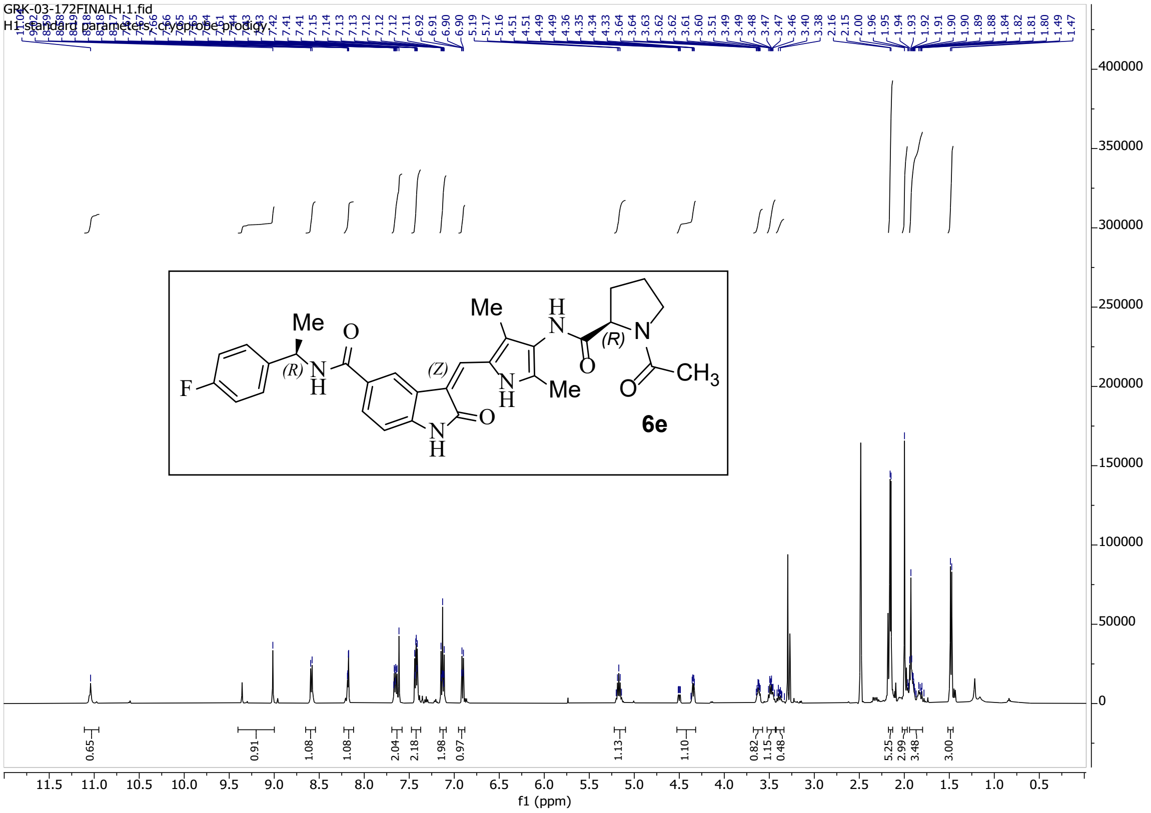

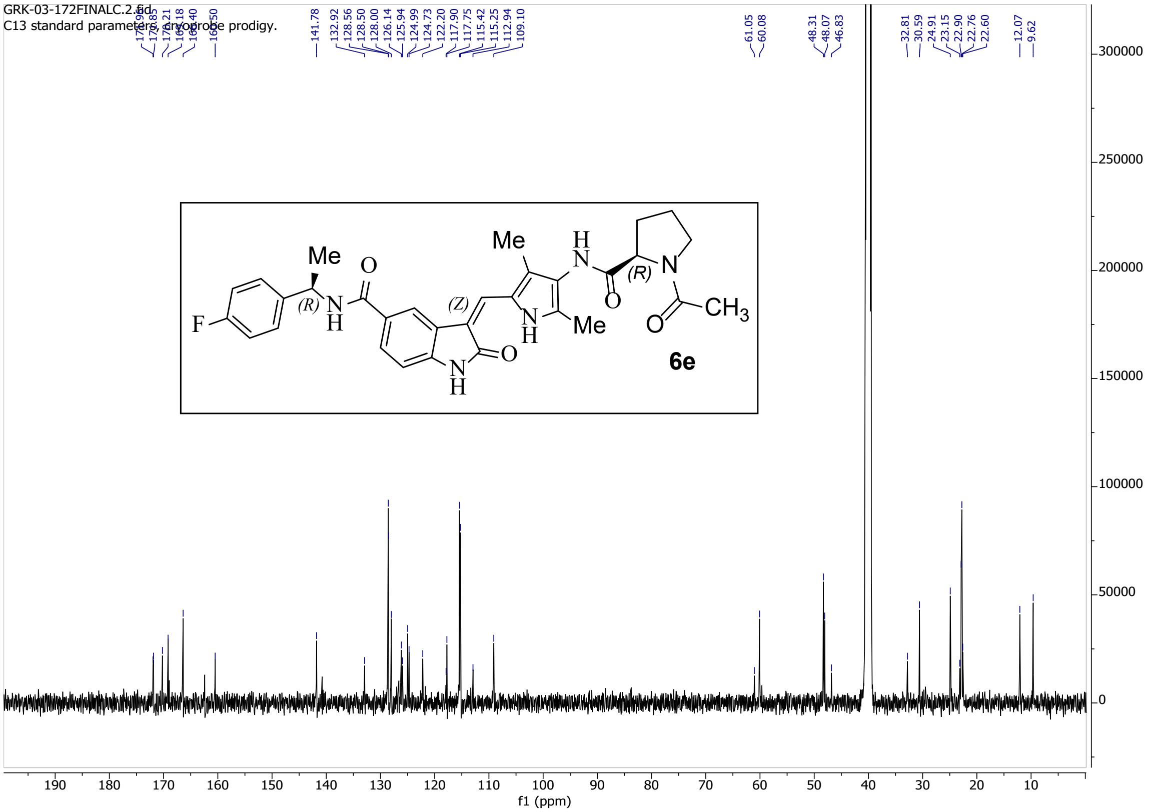

^{1}H & ^{13}C NMR Spectra of Compound 6e (DMSO-d_6)

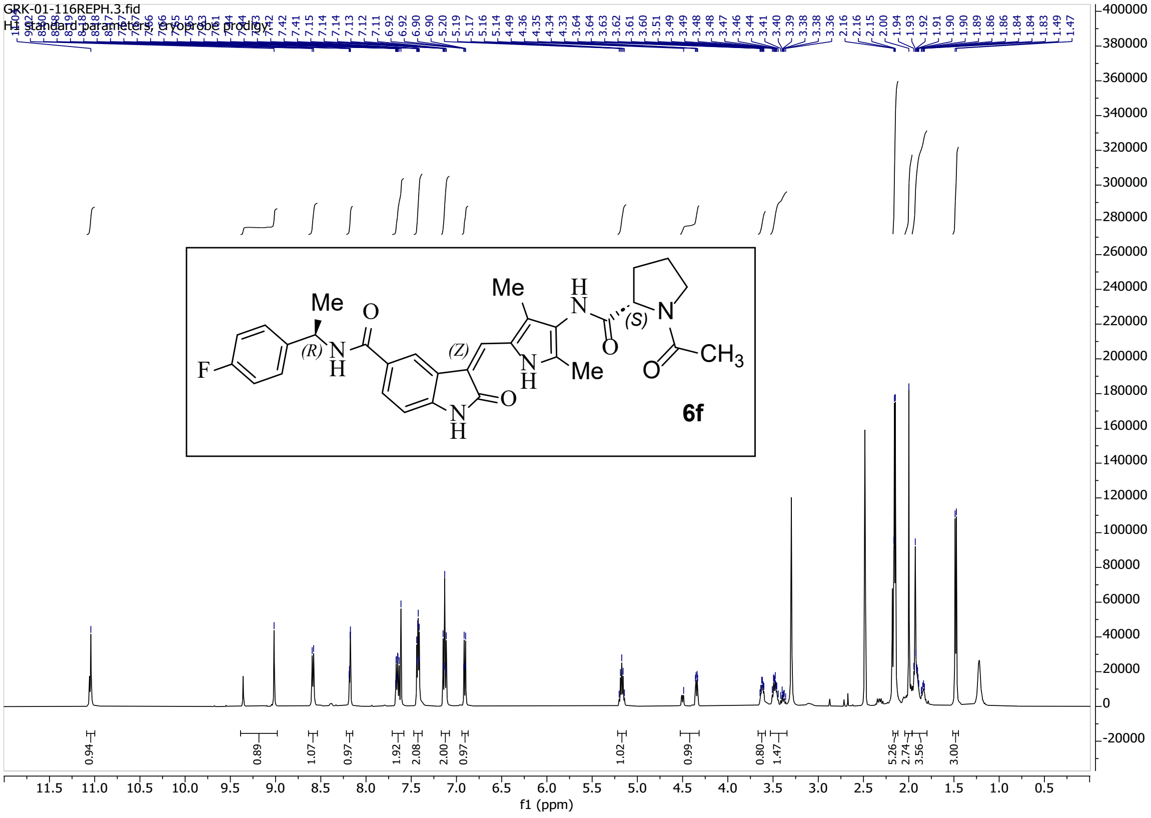

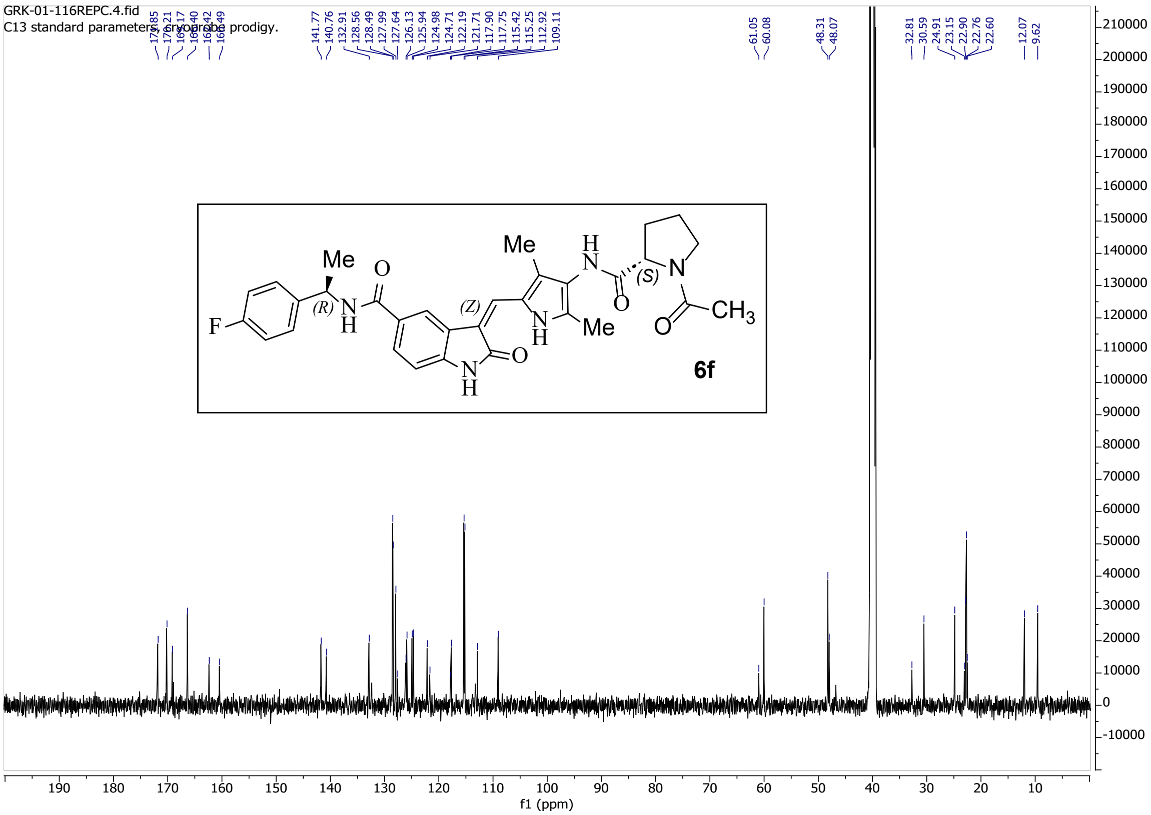

^{1}H & ^{13}C NMR Spectra of Compound 6f (DMSO-d_6)

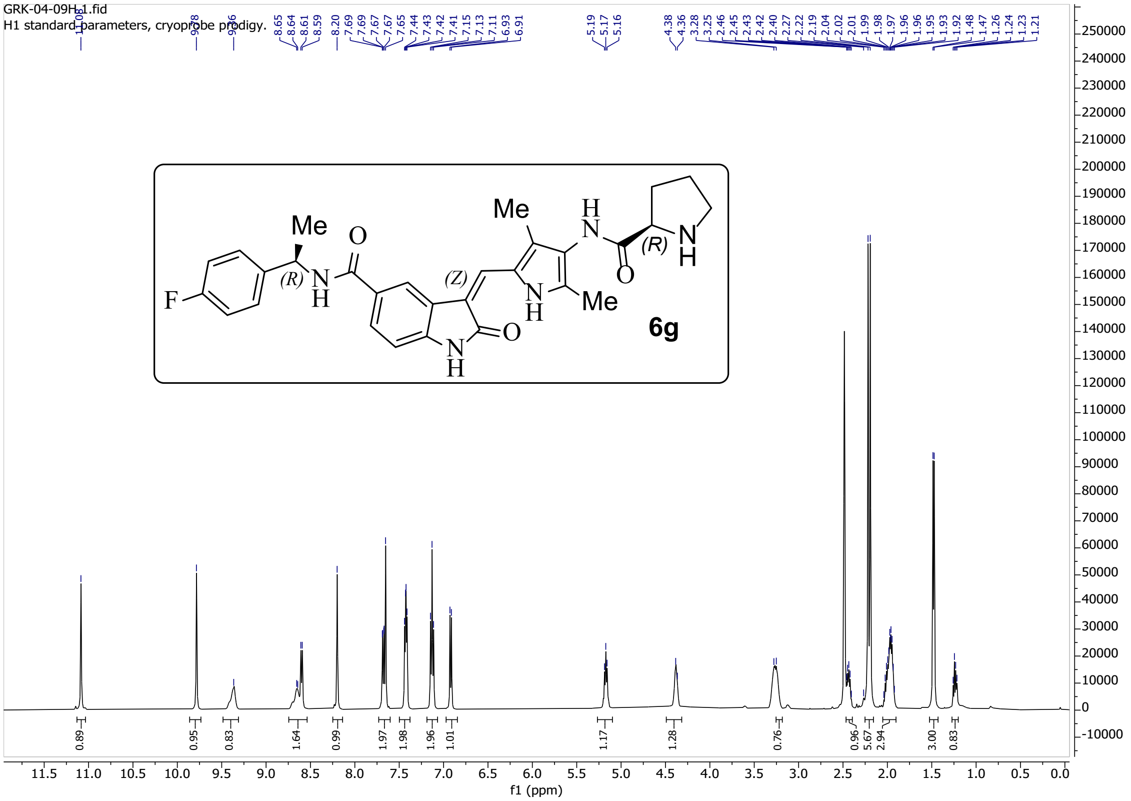

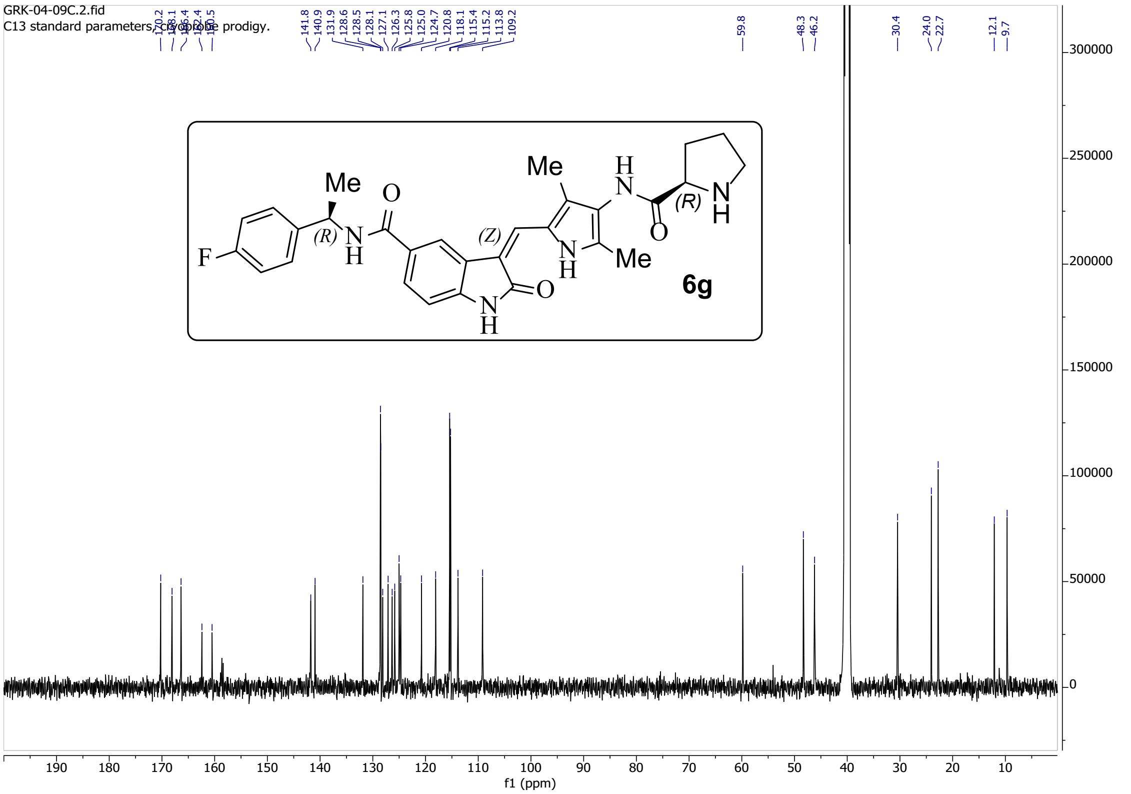

^{1}H & ^{13}C NMR Spectra of Compound 6g (DMSO-d_6)

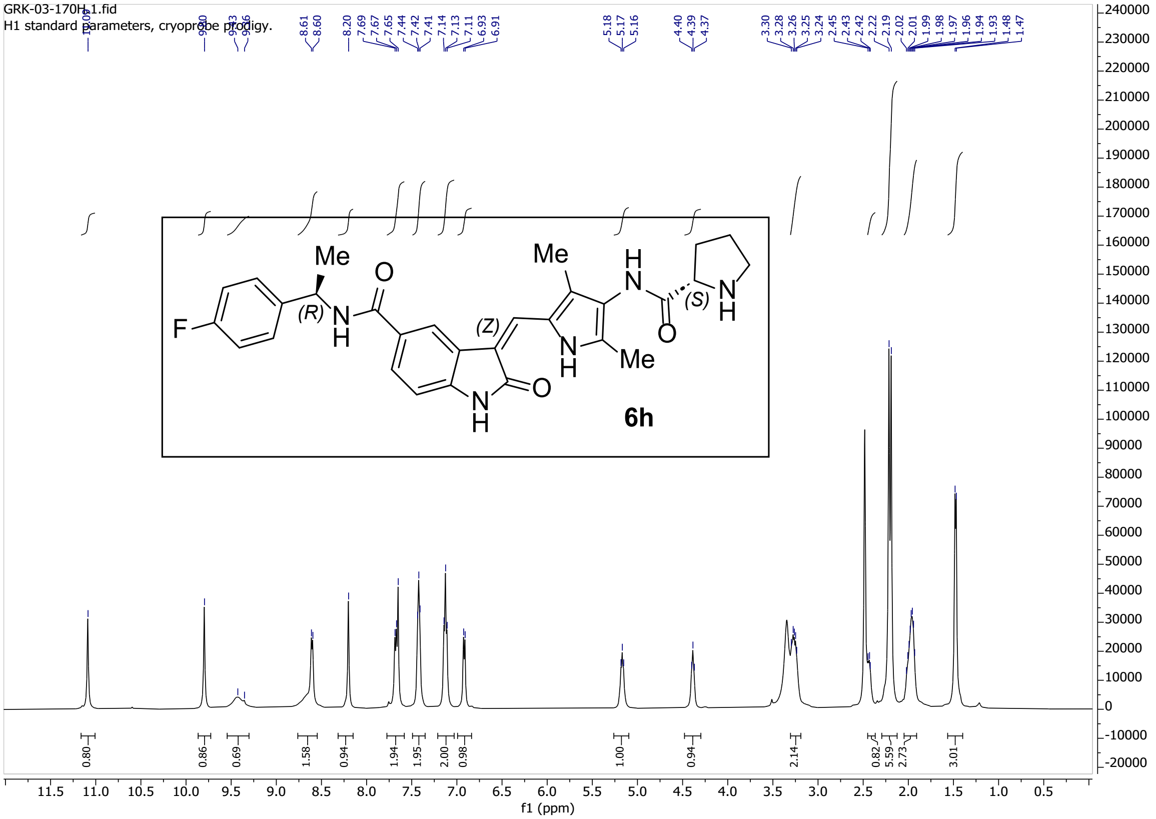

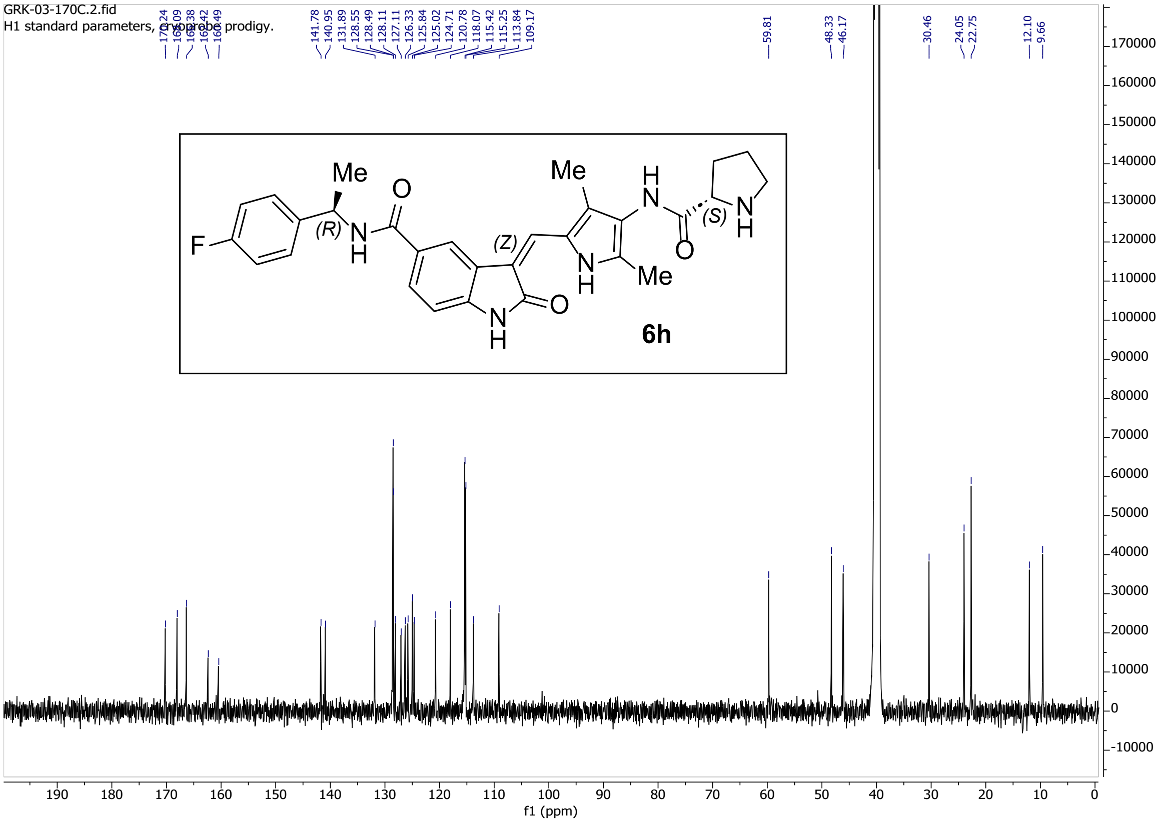

^{1}H & ^{13}C NMR Spectra of Compound 6h (DMSO- d_6)

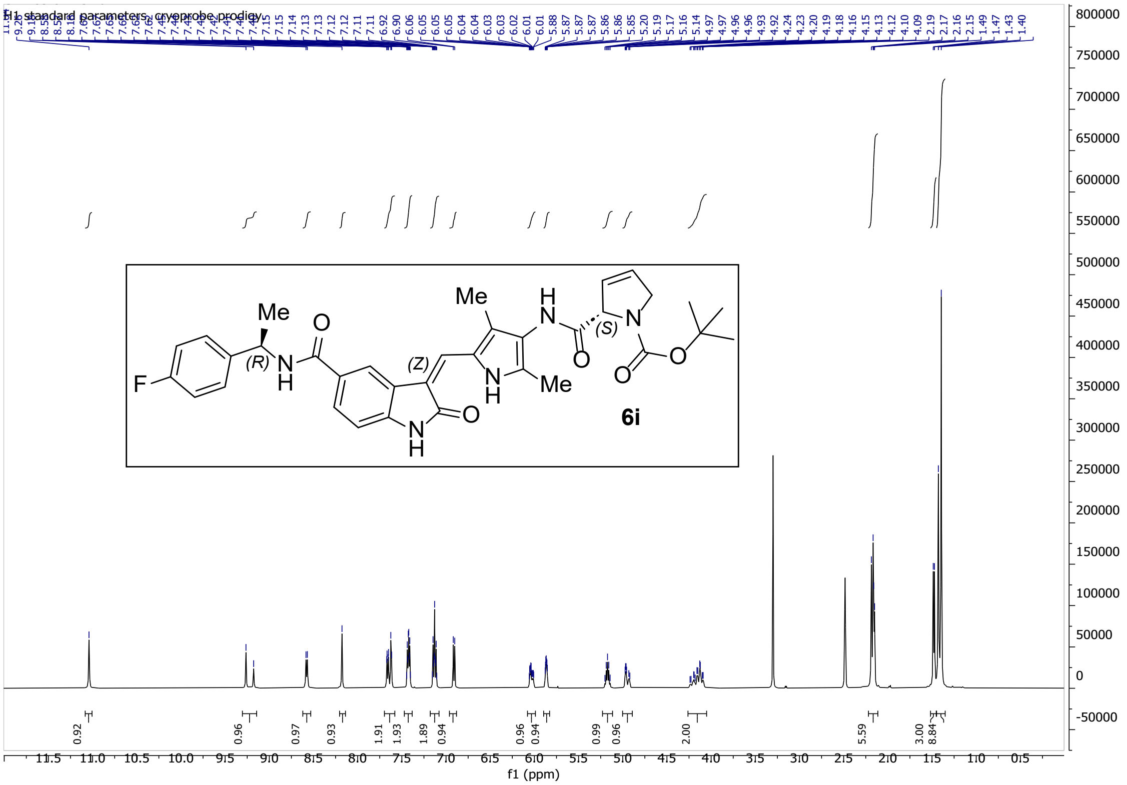

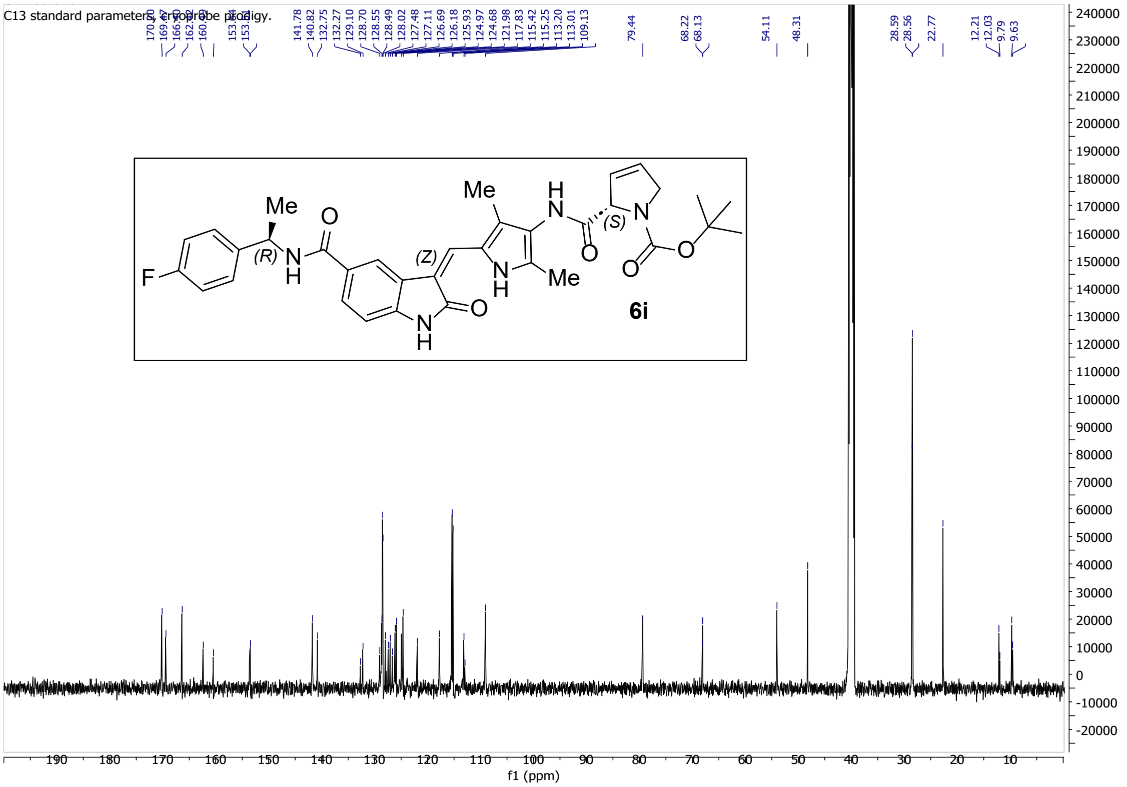

^{1}H & ^{13}C NMR Spectra of Compound 6i (DMSO-d_6)

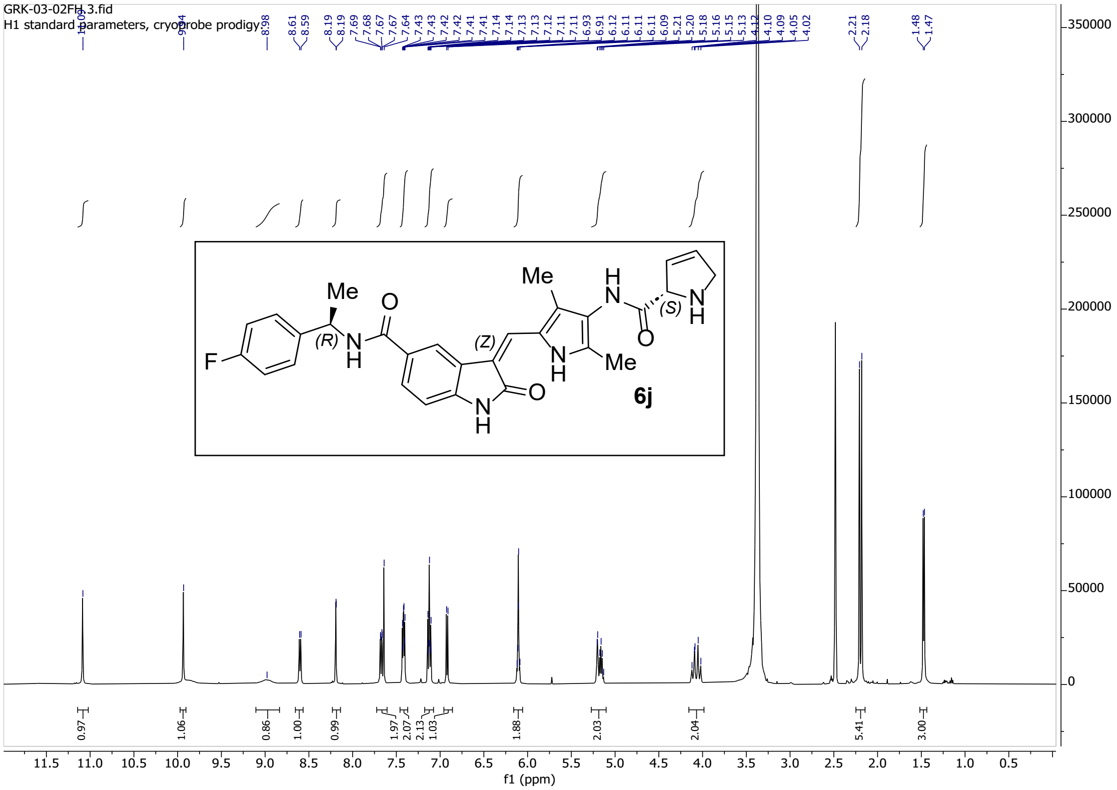

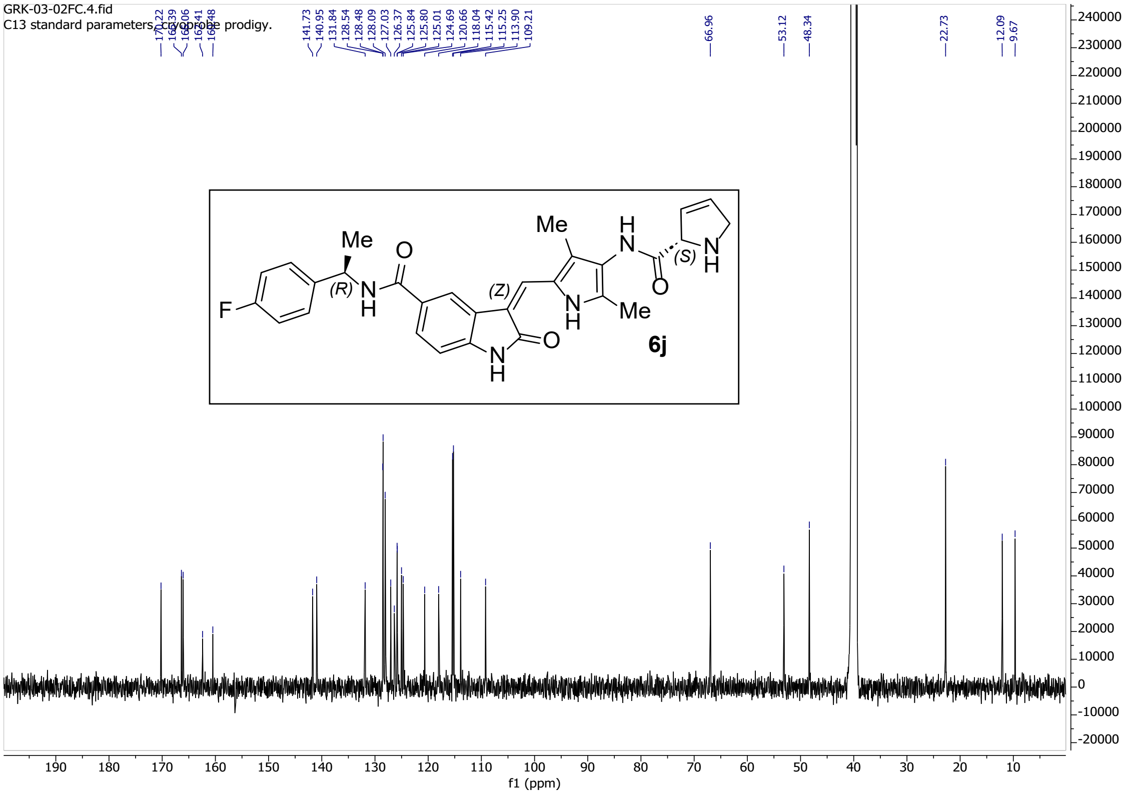

^{1}H & ^{13}C NMR Spectra of Compound 6j (DMSO-d_6)

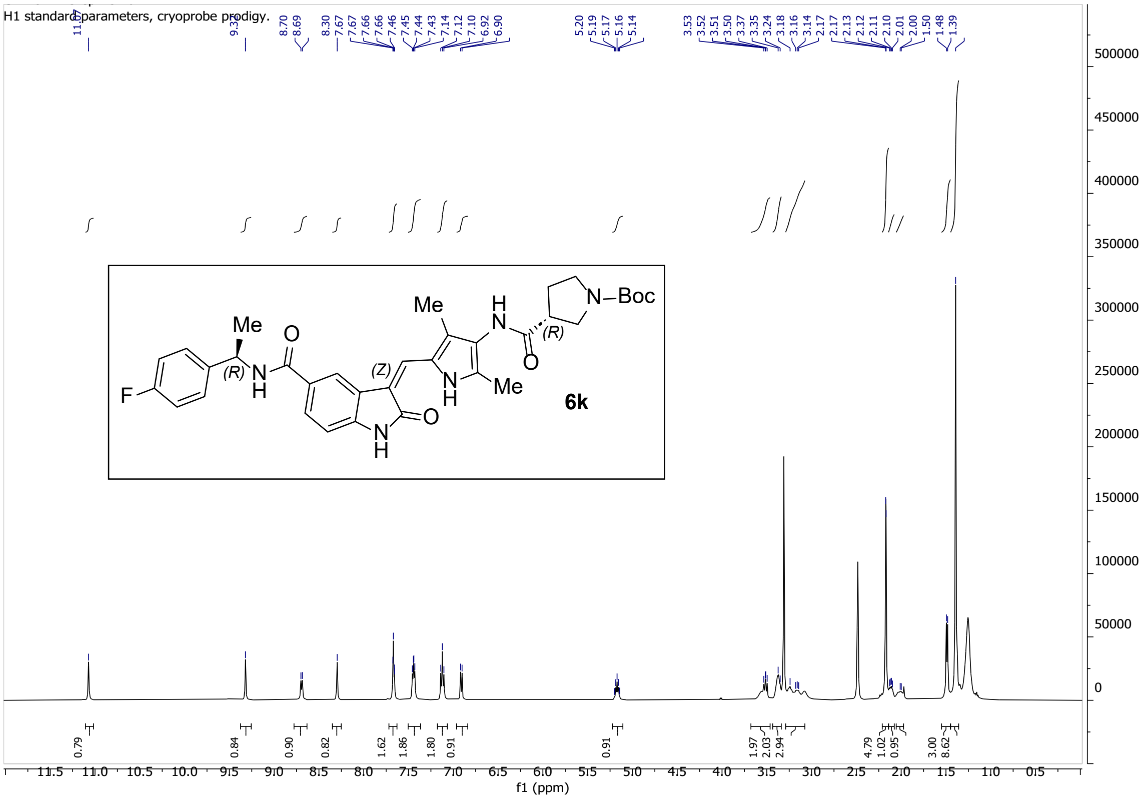

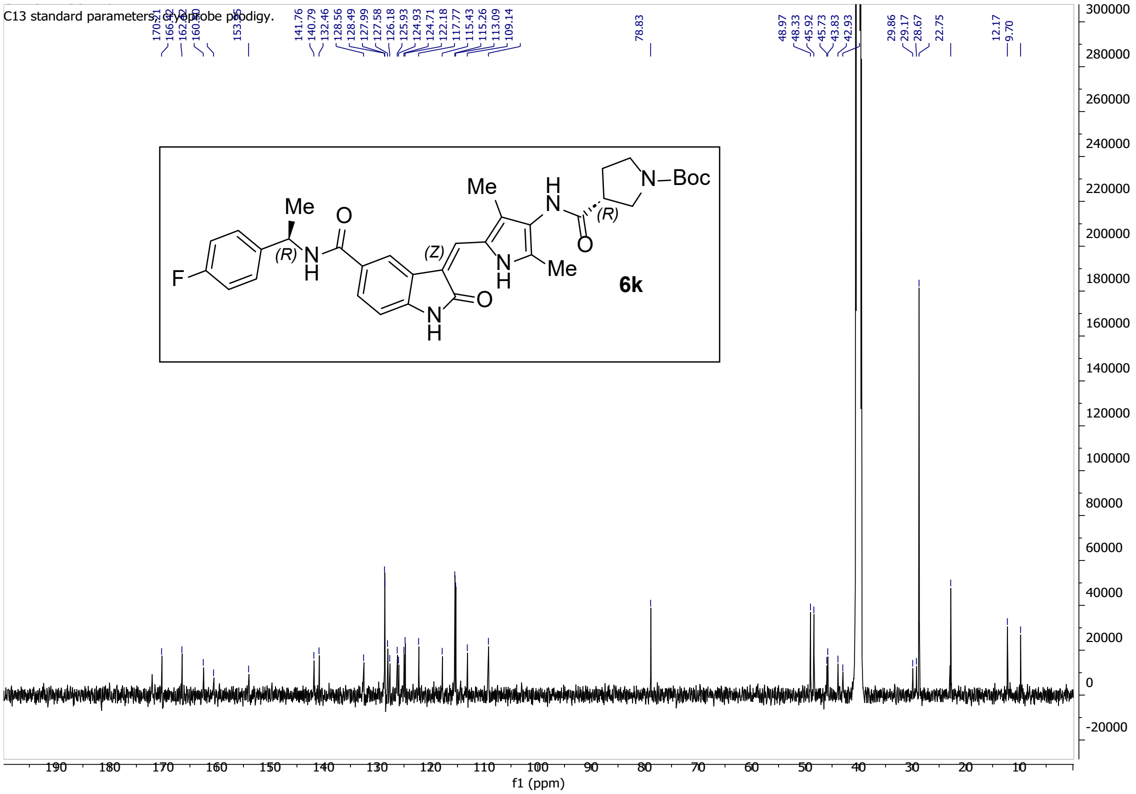

^{1}H & ^{13}C NMR Spectra of Compound 6k (DMSO-d$_6$)

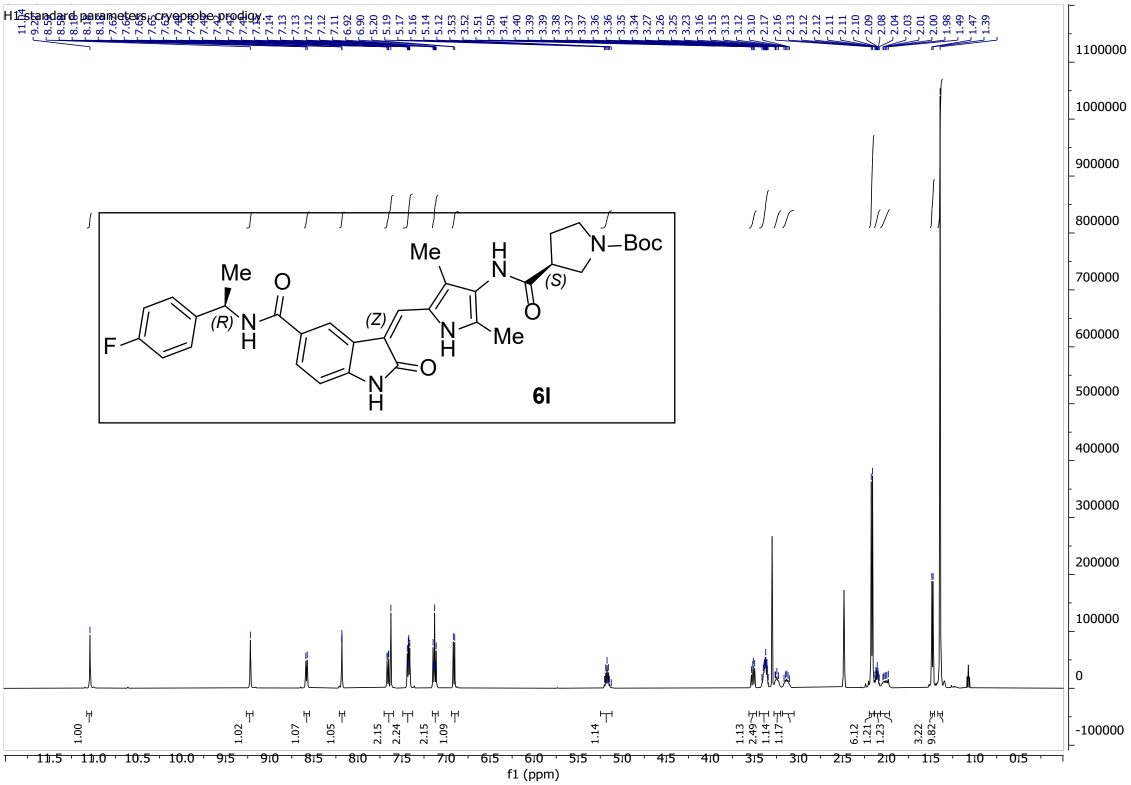

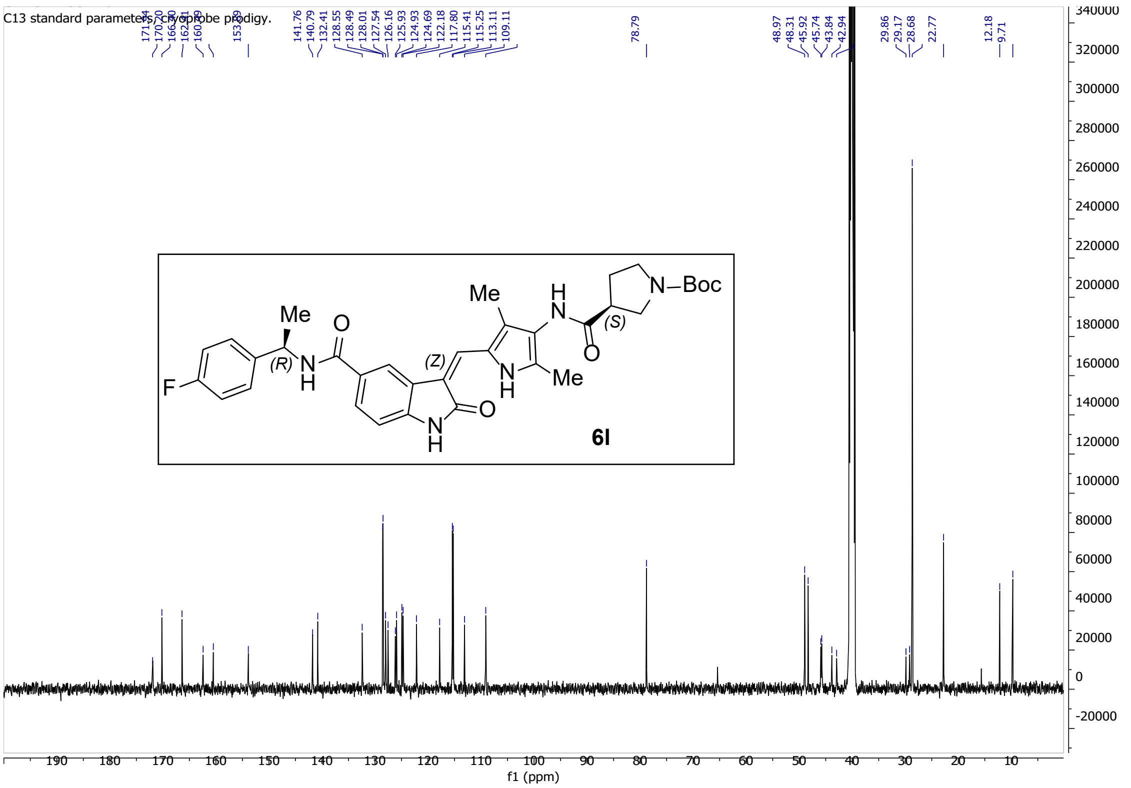

^{1}H & ^{13}C NMR Spectra of Compound 6l (DMSO-d_6)

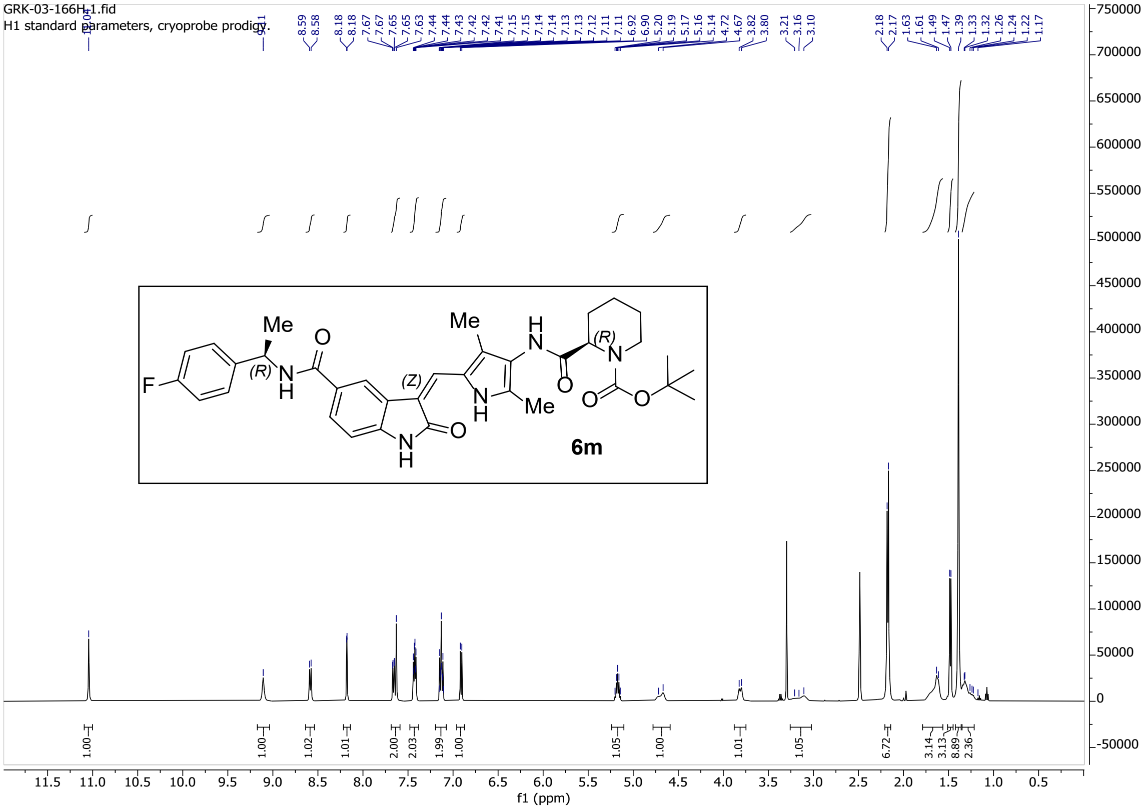

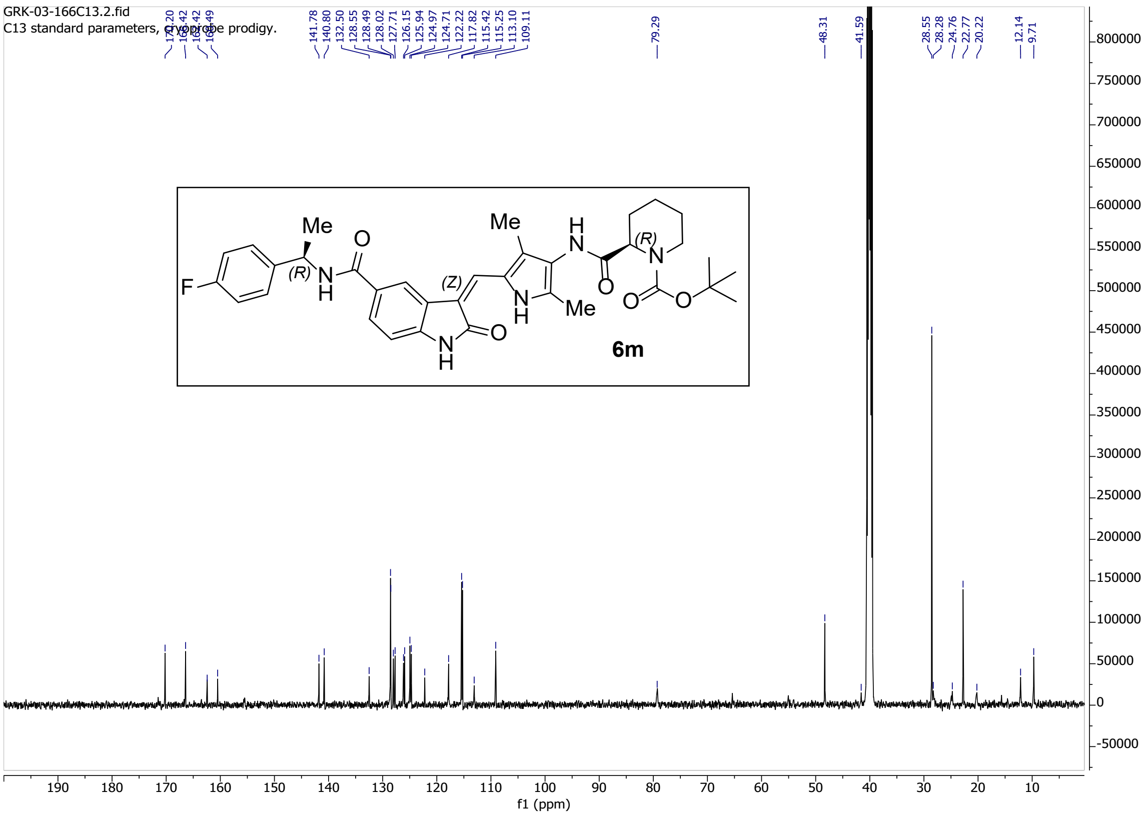

^{1}H & ^{13}C NMR Spectra of Compound 6m (DMSO-d_6)

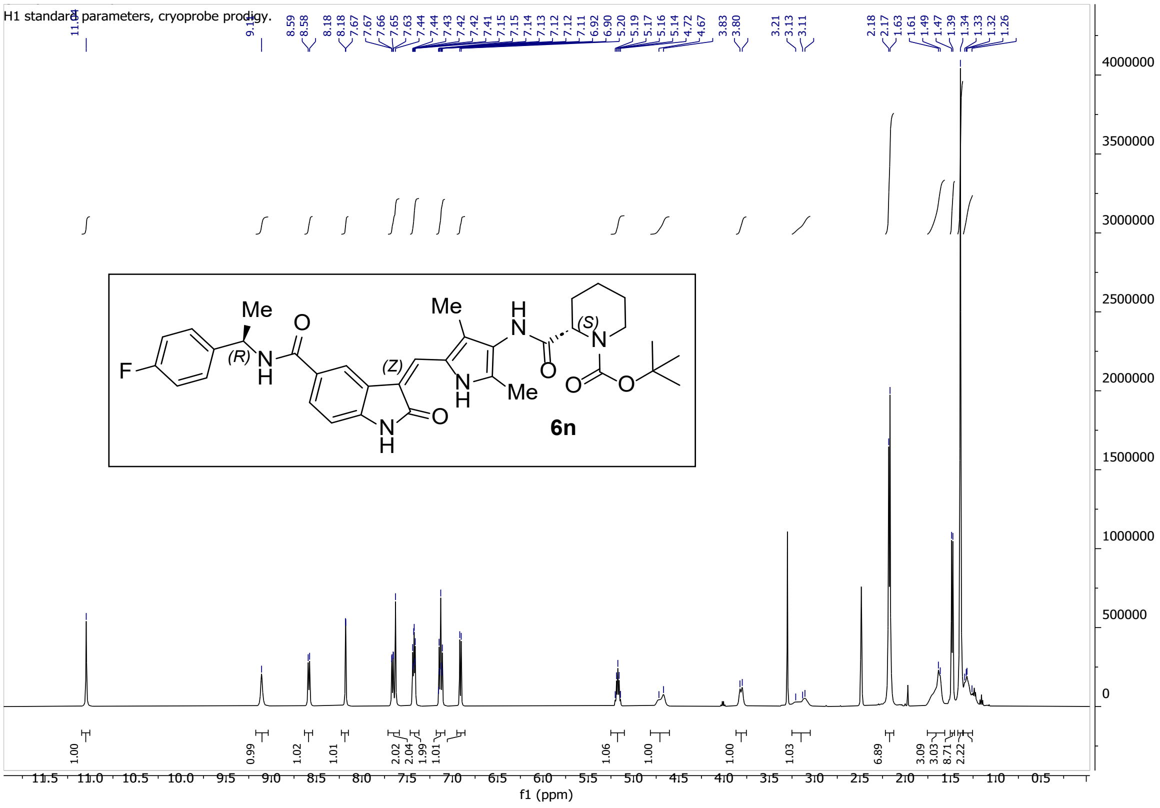

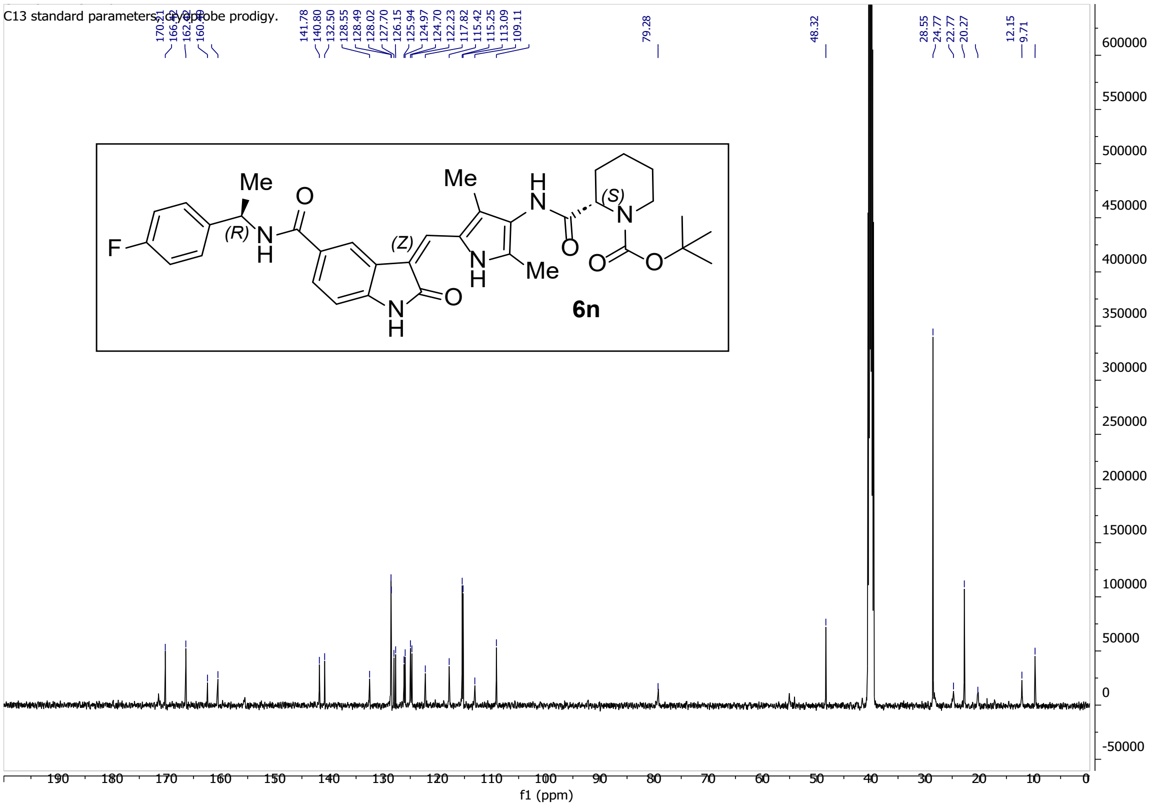

^{1}H & ^{13}C NMR Spectra of Compound 6n (DMSO-d_6)

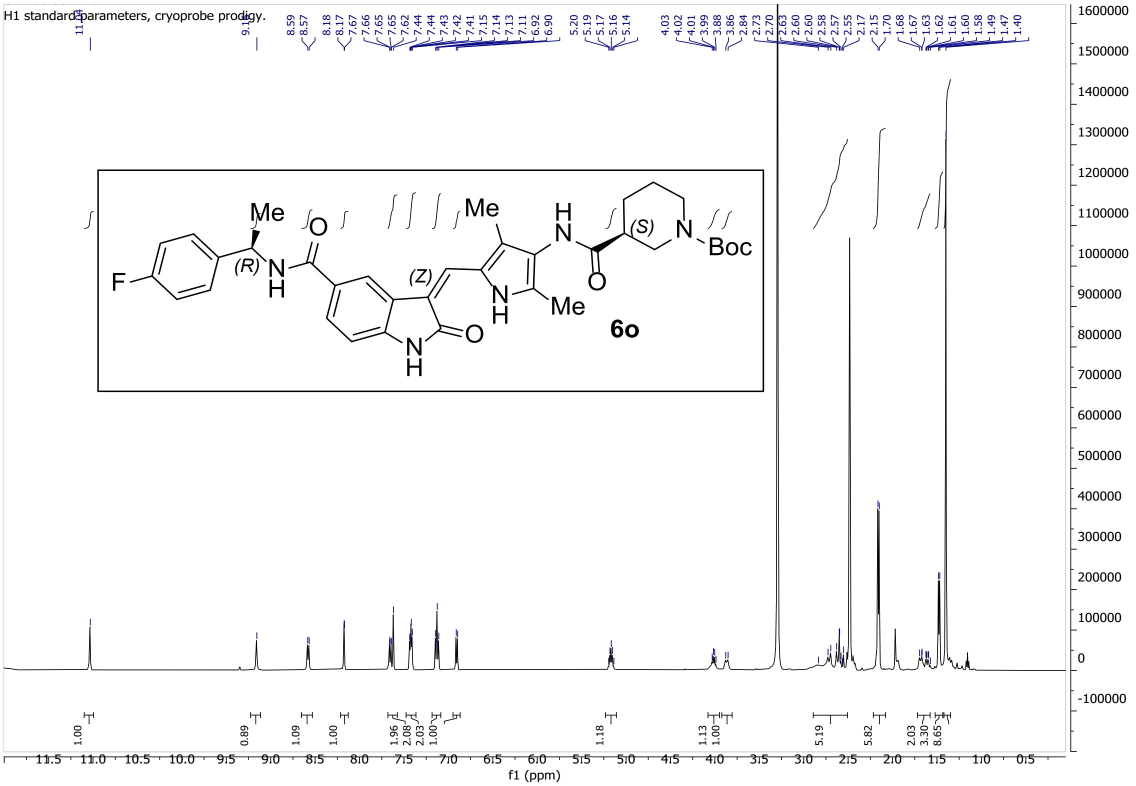

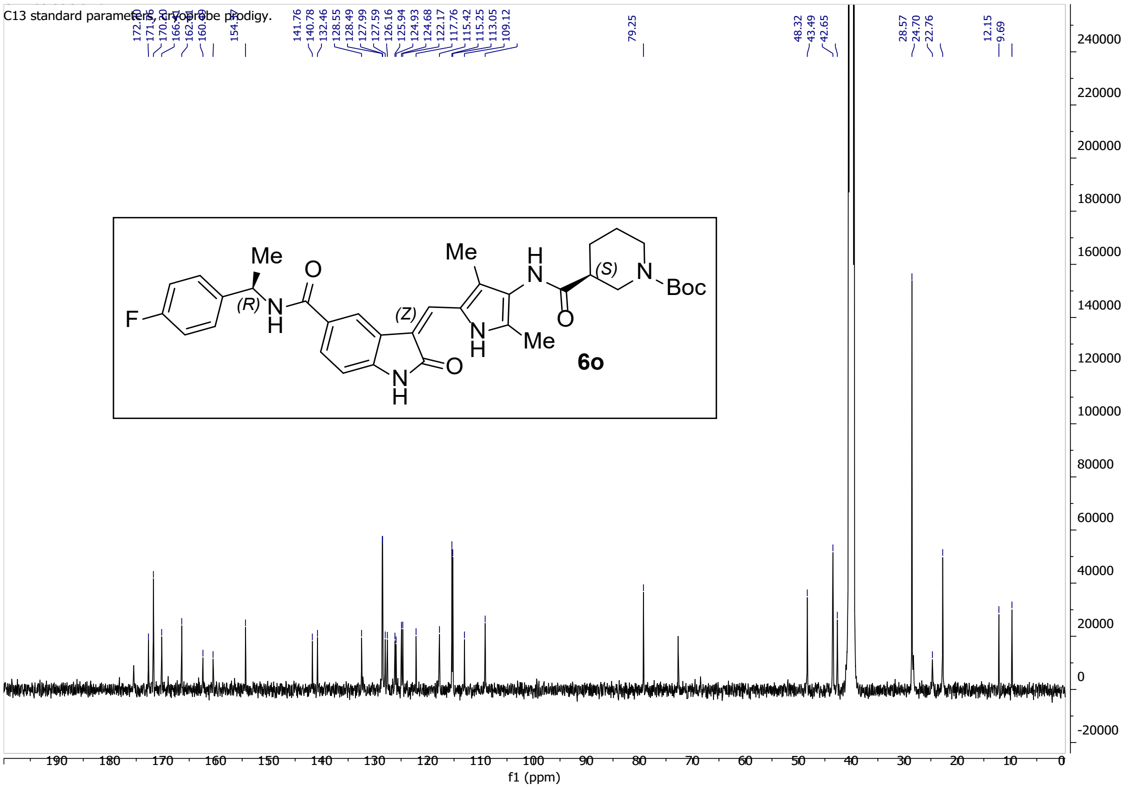

^{1}H & ^{13}C NMR Spectra of Compound 6o (DMSO-d_6)

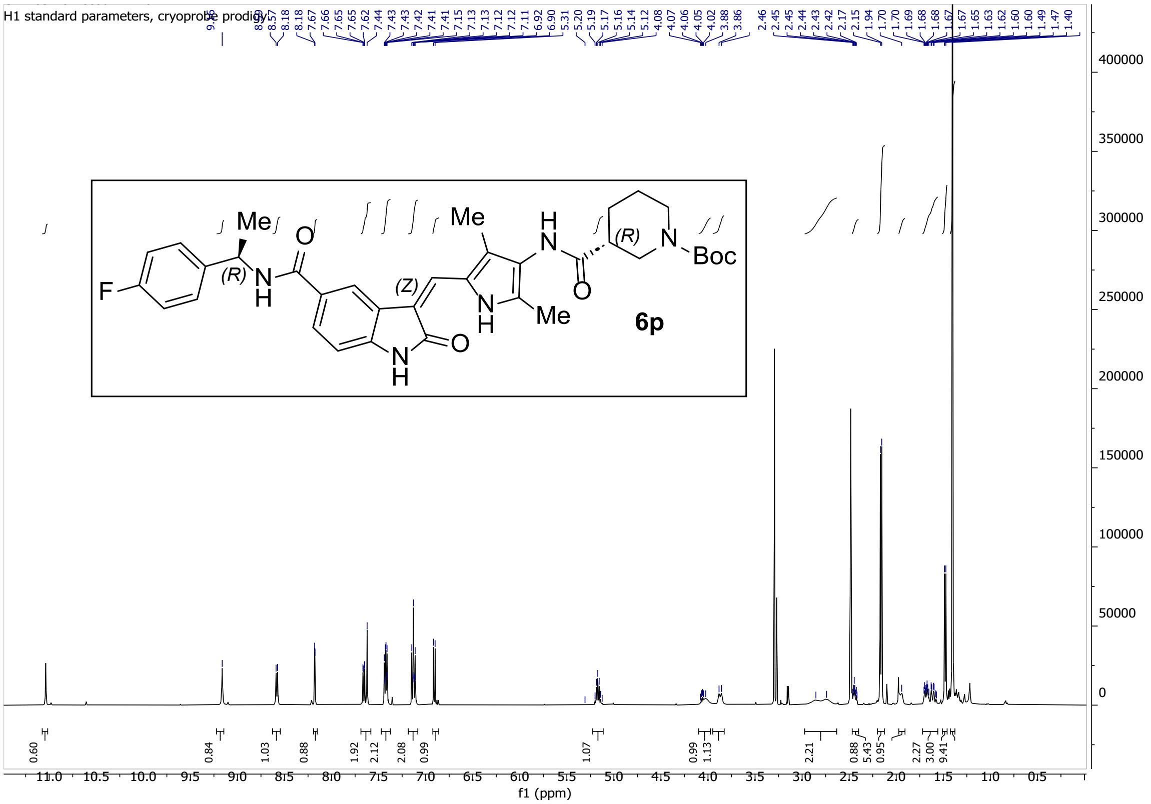

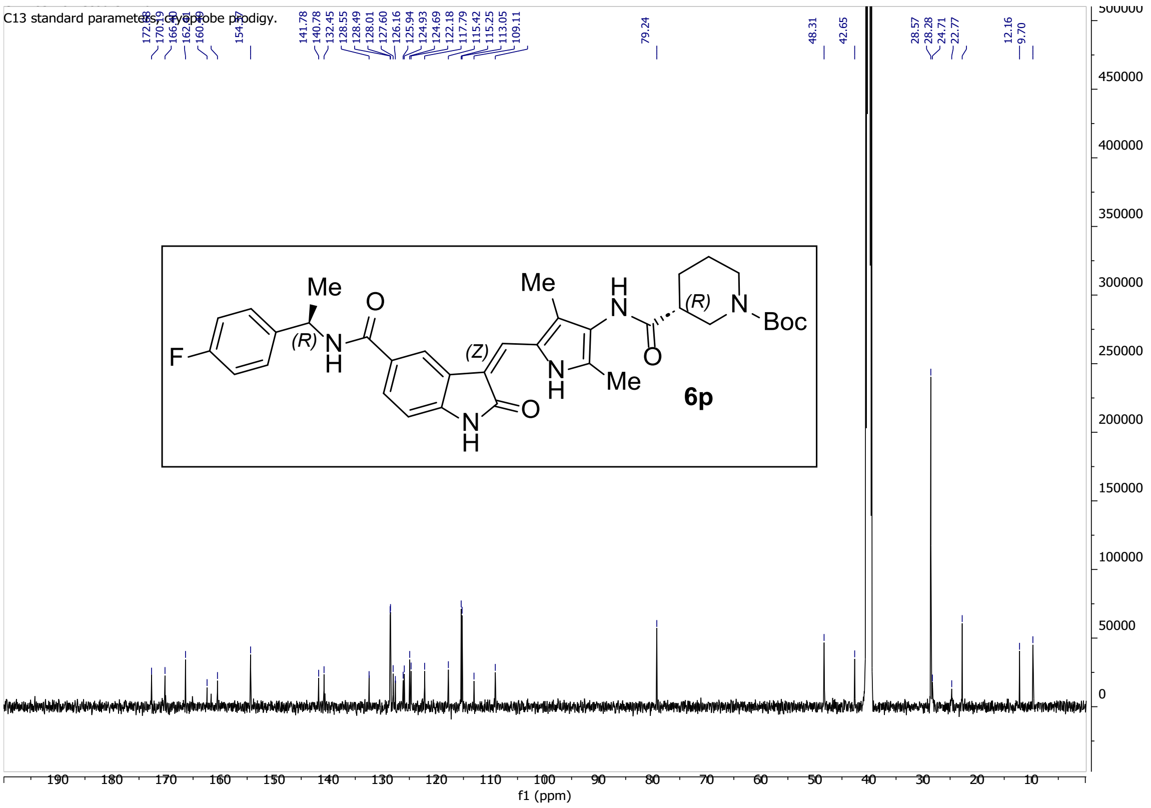

^{1}H & ^{13}C NMR Spectra of Compound 6p (DMSO-d$_6$)

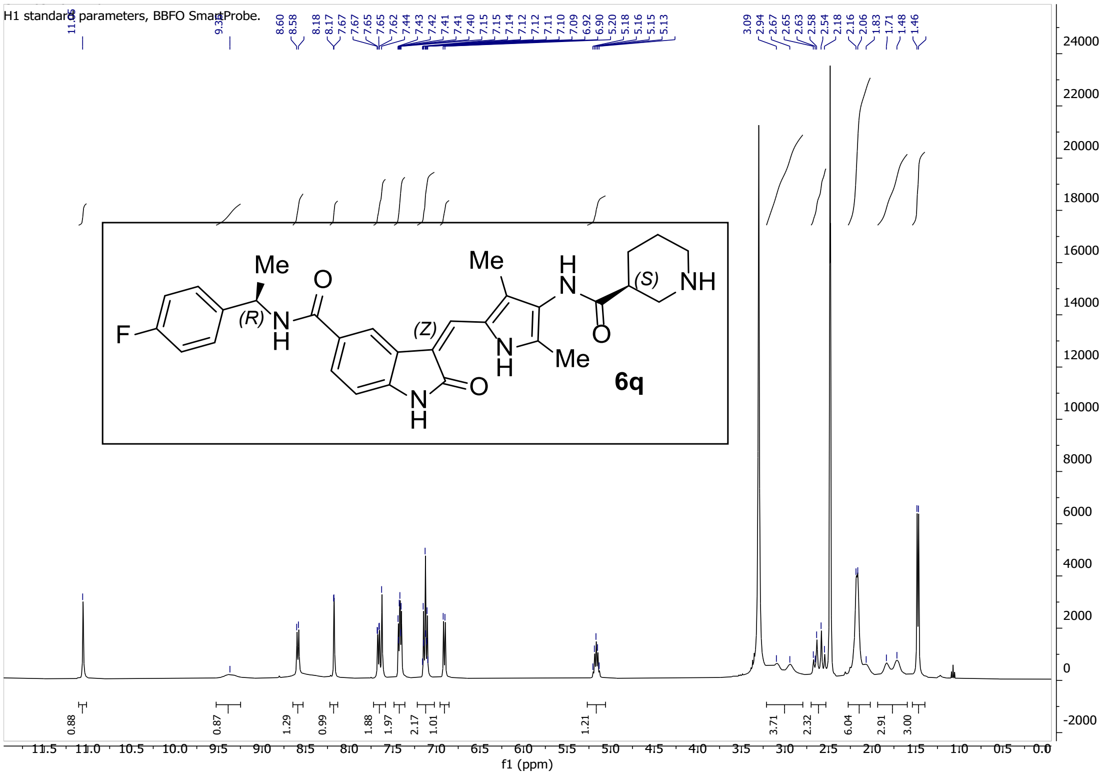

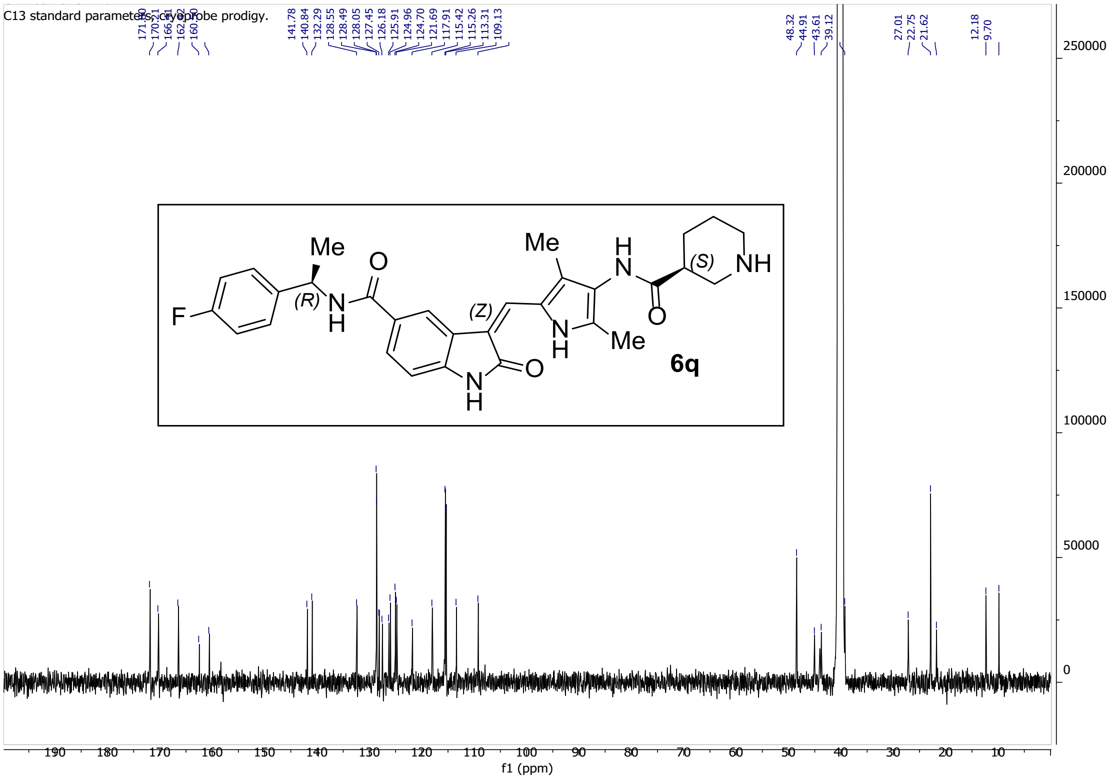

^{1}H & ^{13}C NMR Spectra of Compound 6q (DMSO- d_6)

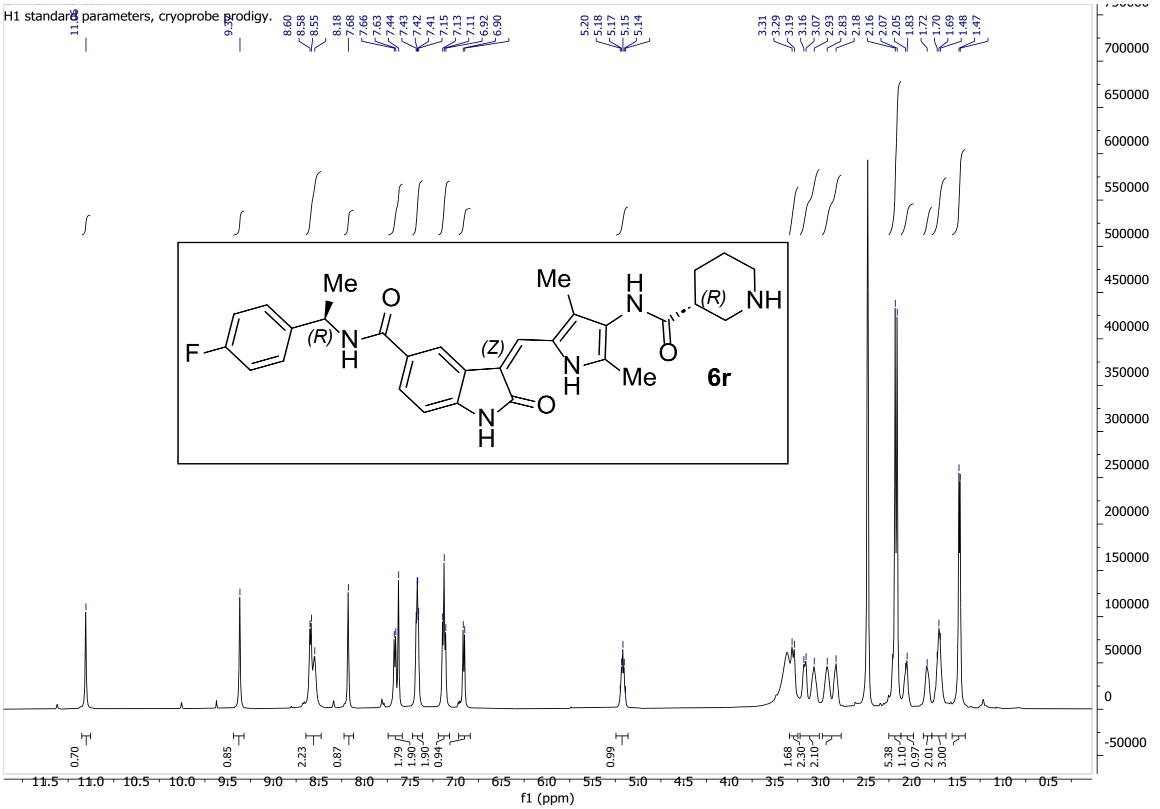

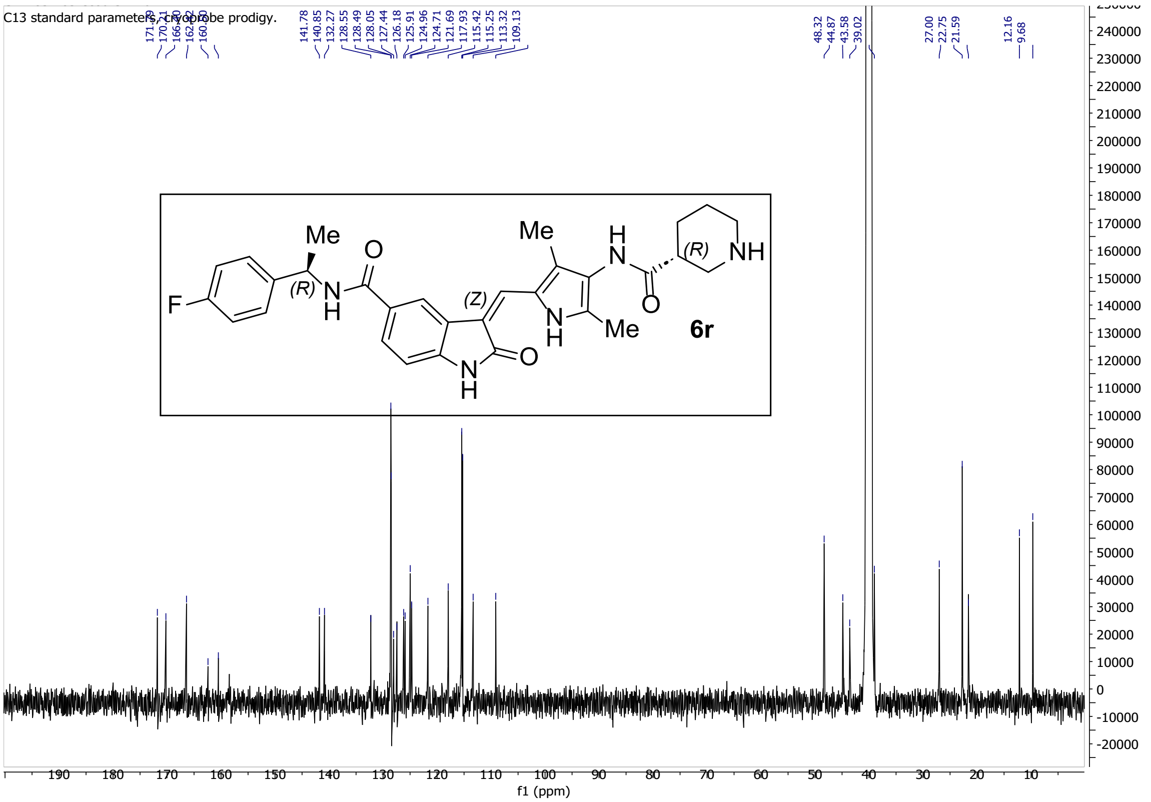

^{1}H & ^{13}C NMR Spectra of Compound 6r (DMSO-d_6)

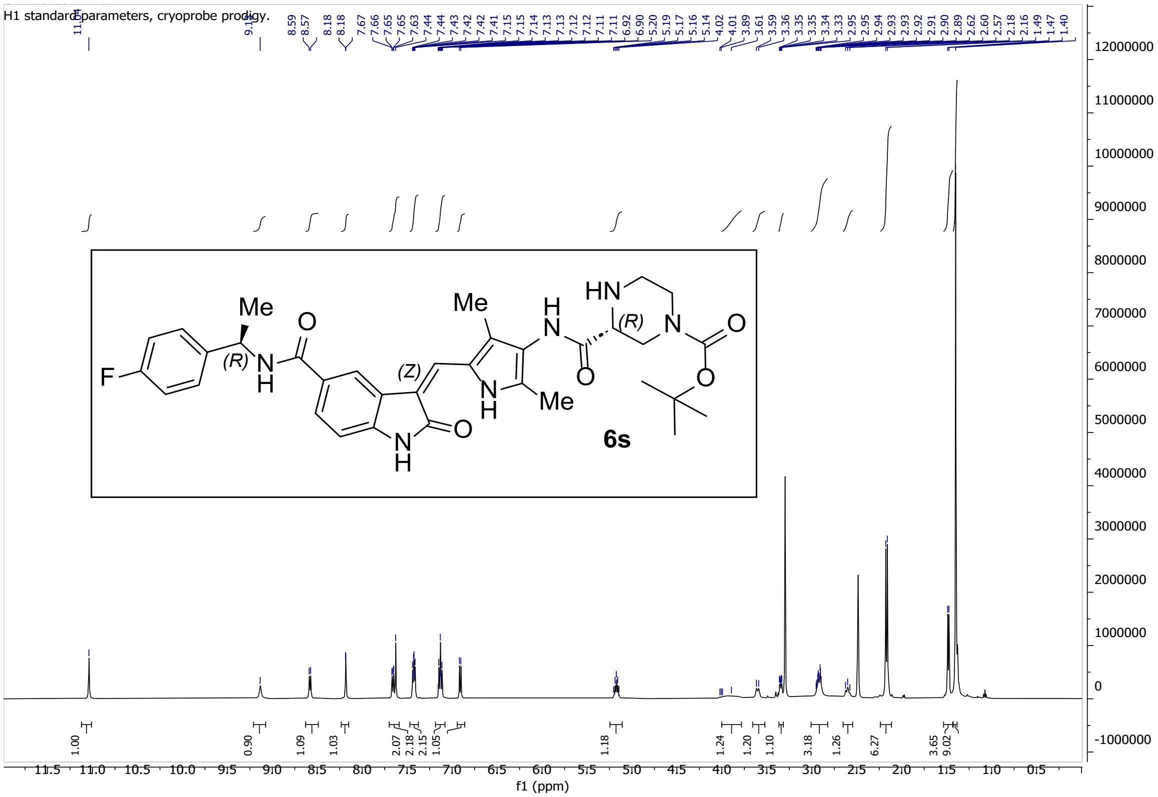

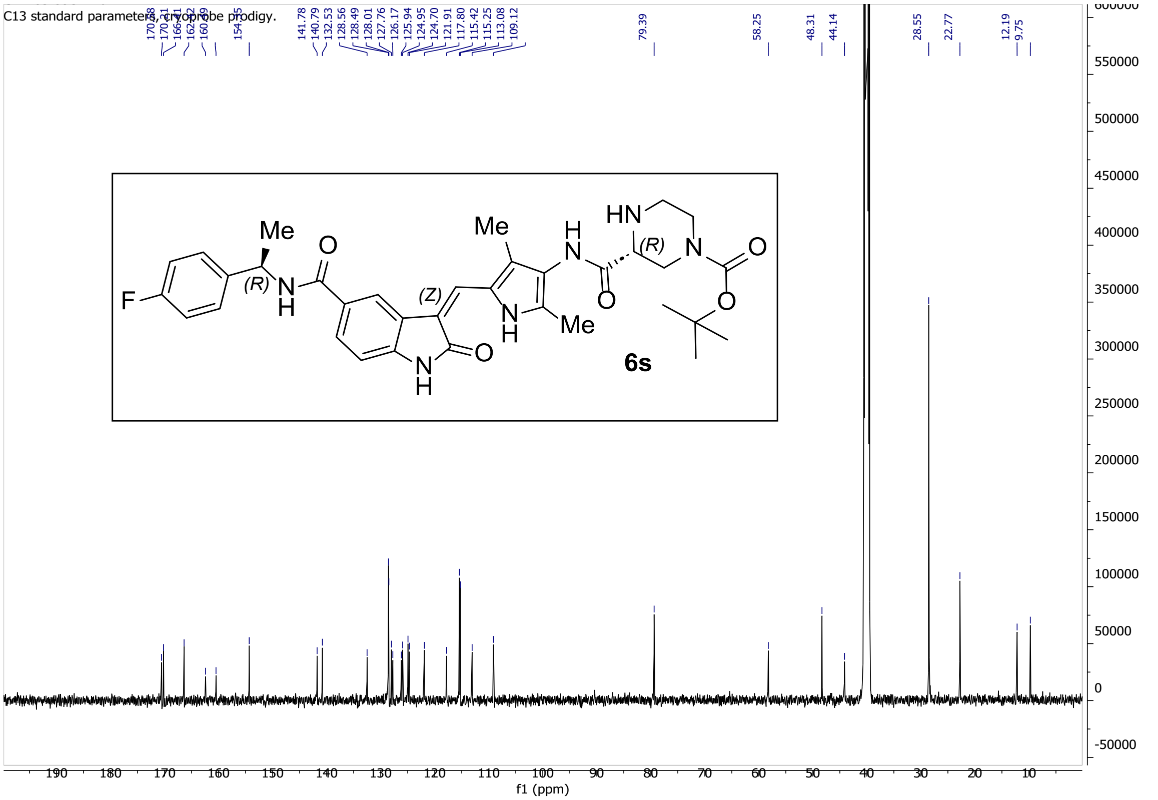

^{1}H & ^{13}C NMR Spectra of Compound 6s (DMSO-d_6)

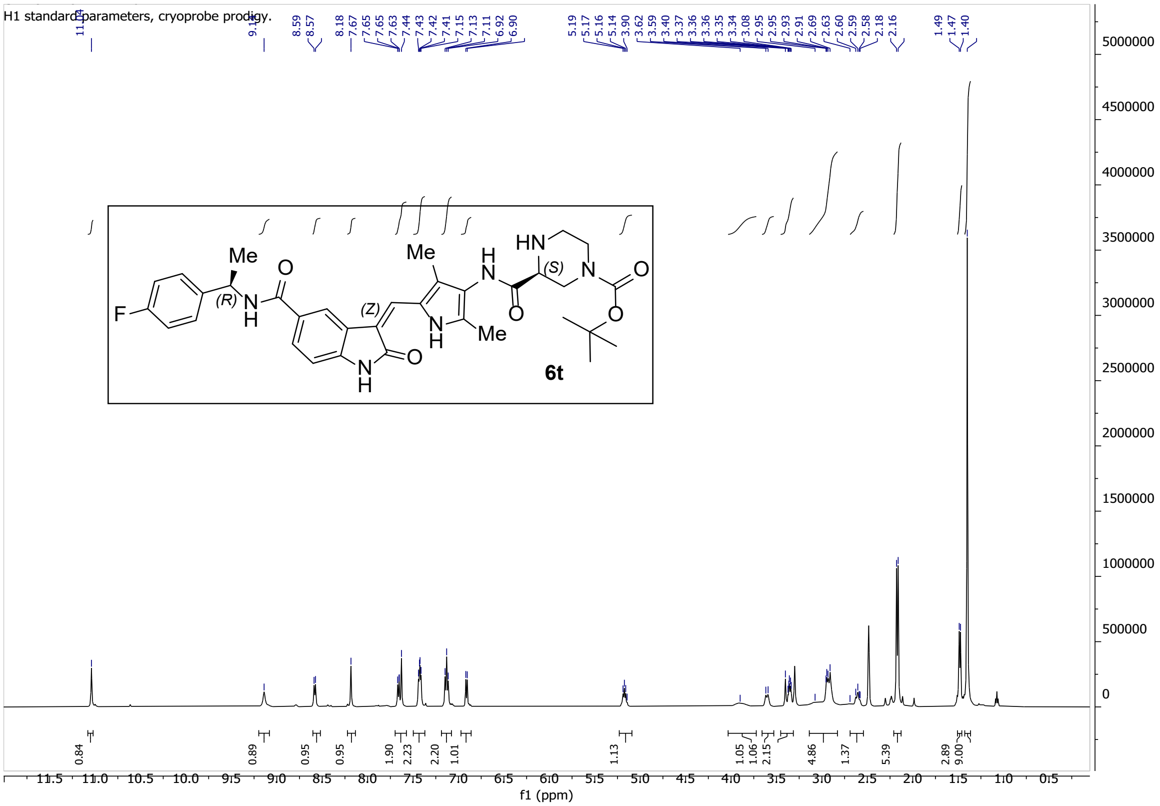

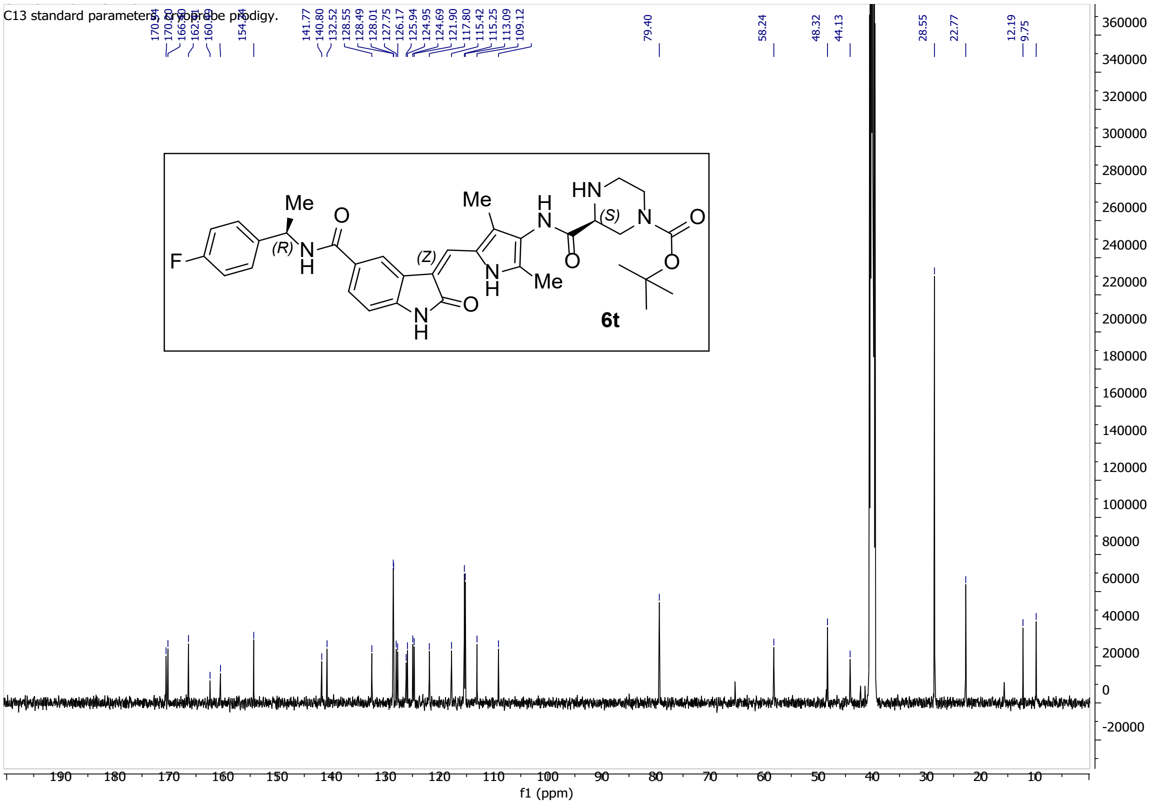

^{1}H & ^{13}C NMR Spectra of Compound 6t (DMSO-d_6)

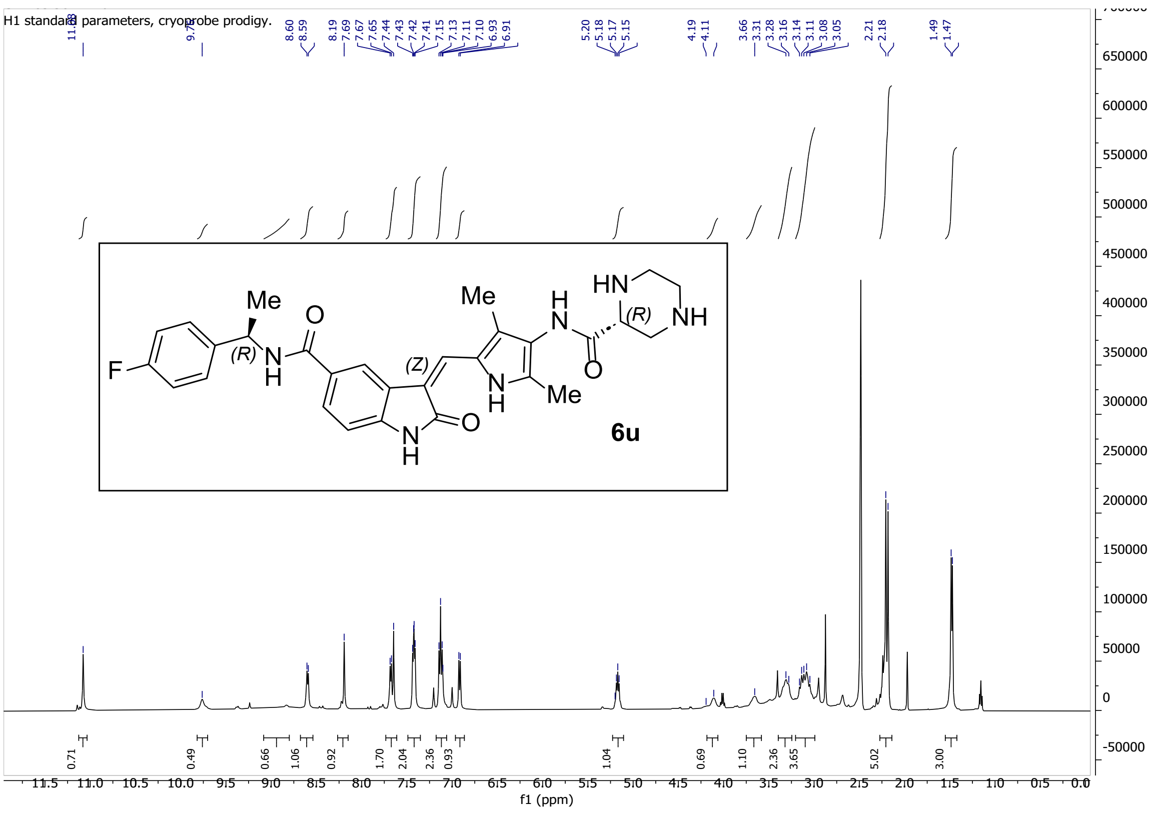

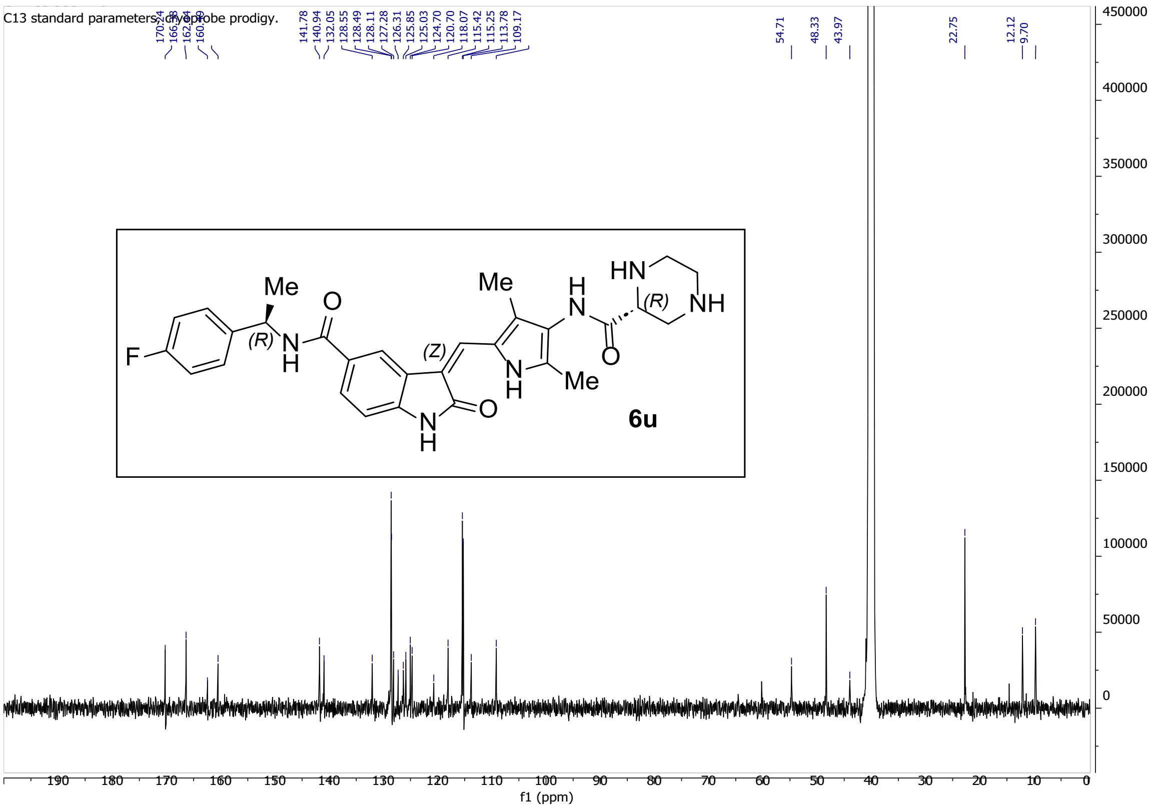

^{1}H & ^{13}C NMR Spectra of Compound 6u (DMSO-d$_6$)

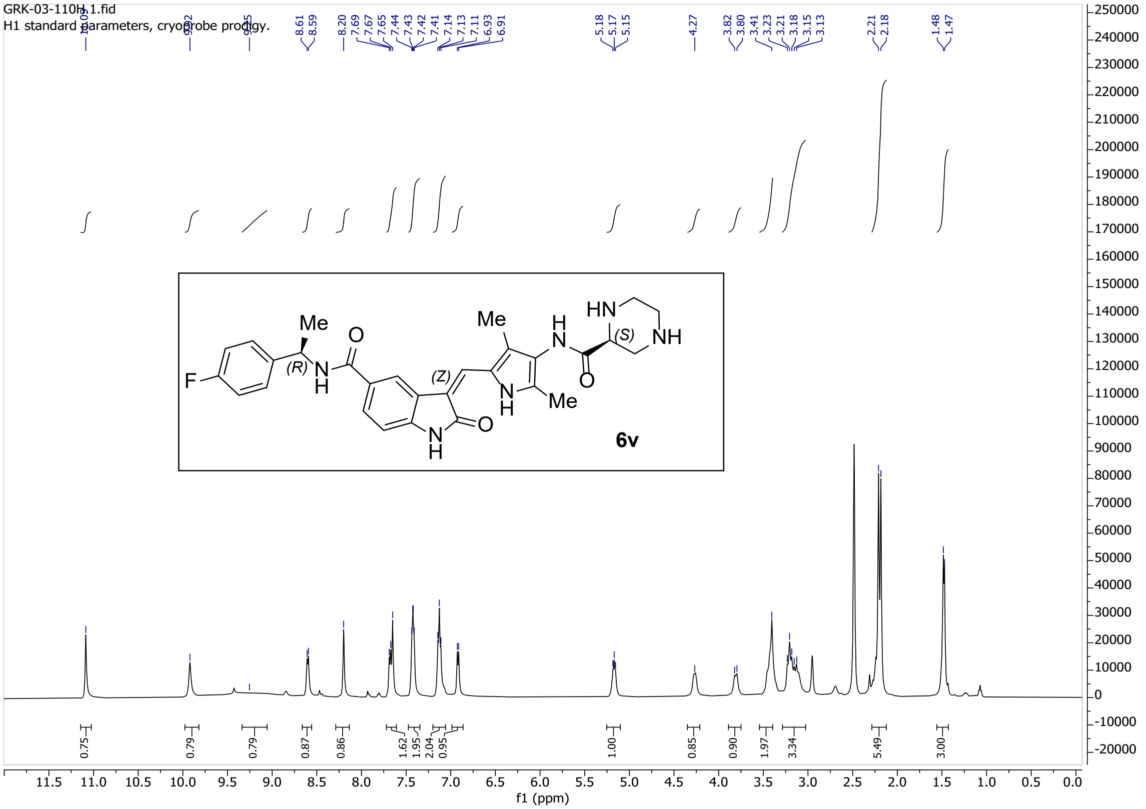

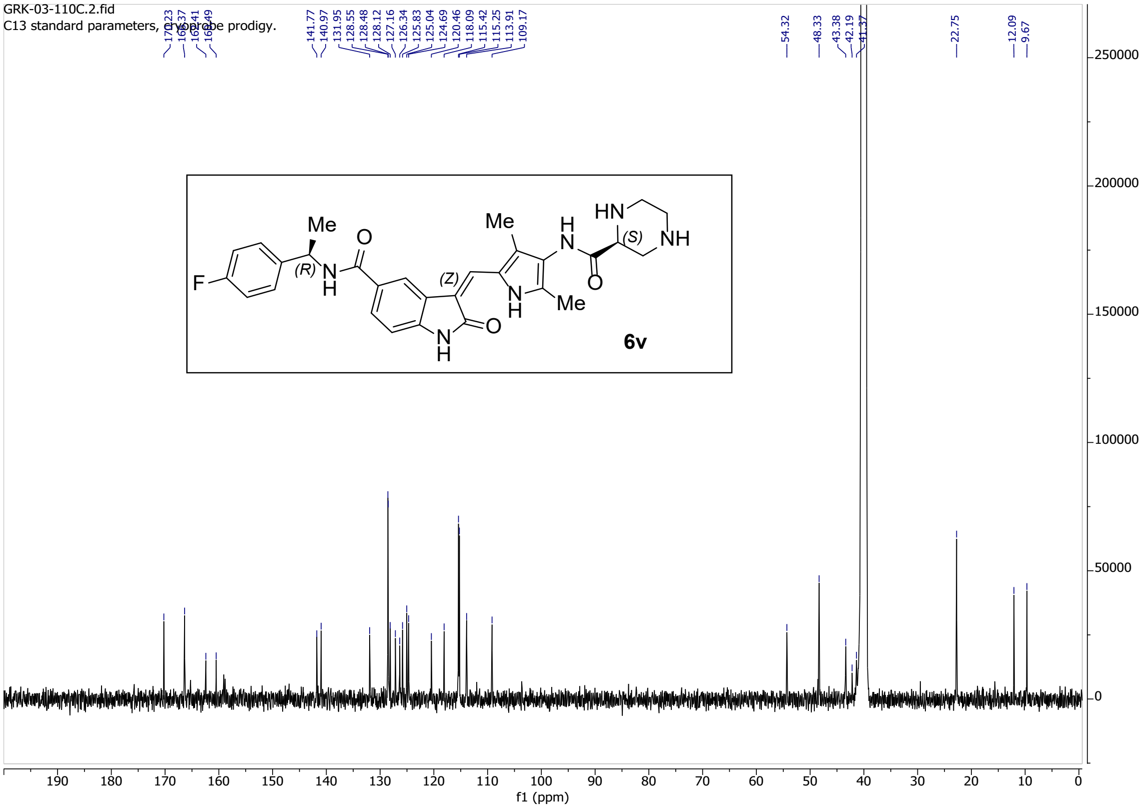

^{1}H & ^{13}C NMR Spectra of Compound 6v (DMSO-d_6)
